# Supplementary material for: Interest without uptake: A mixed-methods analysis of methadone utilization in Kyrgyz prisons
Source: PLoS One. 2022 Oct 25;17(10):e0276723. doi: 10.1371/journal.pone.0276723 (PMC9595522; doi:10.1371/journal.pone.0276723)
Supplement: S1 File — Full list of survey questions asked of all participants and codebook for reading data. (PDF) [file pone.0276723.s001.pdf]

MATLINK All PID 1265

Codebook

Data Dictionary Codebook

11/03/2020 10:53am

Collapse all instruments

	#	Variable / Field Name	Field Label<br>*Field Note*	Field Attributes (Field Type, Validation, Choices, Calculations, etc.)
Instrument: **Form 2** (form_2) Collapse				
	1	redcap_id	Redcap ID	text
	2	language	Language	radio<br>2 Russian<br>3 Romanian
	3	interviewer_id	Interviewer ID<br>U: int_id_ru<br>M: int_id_ru int_id_om<br>K: int_id_ru	text (number, Min: 1, Max: 60)
	4	country	Country<br>U: not present, coded from pid_ru<br>M:coded from pid_ru. pid_om<br>K: coded from pid_ru	radio<br>3 Ukraine<br>1 Moldova<br>2 Kyrgyzstan
	5	screen_date	Today's Date (Date of Screening)<br>U: scr_dt_ru<br>M: scr_dt_ru, scr_dt_om<br>K: scr_dt_ru	text (date_mdy), Identifier
	6	probation_prison	Probation or Prison<br>U: propriru<br>M: propriru, propriom<br>K: propriru	radio<br>1 Probation<br>2 Prison
	7	matlink_id	MATLINK ID:<br><br>U:pid_ru<br>M:pid_ru, pid_om<br>K: pid_ru<br><br>(11 symbols):<br>Country code (Ukraine -3)<br>Facility number (- 01,02, 03, 04, 05, 06)<br>Gender (1 - male, 2 - female)<br>Study phase = 1<br>Last number of the day of birth<br>Last number of the month of birth<br>Last number of the year of birth<br>First letter of the client's first name<br>First letter of the client's last name<br>First letter of the mother's first name	text, Identifier
	8	syrex<br><br>Show the field ONLY if:<br>[country] = '3'	Participant SyrEx code (8 symbols):<br>U:syr_ru<br><br>First letter of the client's name<br>First letter of the mother's name,<br>First letter of the father's name,<br>Day of birth (two numbers),<br>Two last numbers of year of birth<br>Client's gender: ? of F	text, Identifier

	9	kyr_id_code<br><br>Show the field ONLY if:<br>[country] = '2'	Kyrgyzstan National ID Code:<br><br>First two letters of mother's name<br>First two letters of father's name<br>Gender(1-male, 2-female)<br>Last two digits of the birth year of the participant<br>U: N/A<br>M: N/A<br>K: idkyrru	text, Identifier
	10	mat_syr	?	text, Identifier
	11	rods_label	Section Header: *Part 1 RODS/screening*<br><br>1. Have you used any of the following drugs in the past 12 months?	descriptive
	12	heroin	Section Header: *1. Have you used any of the following drugs in the past 12 months?*<br>a. Heroin U: f2_1a_ru, M: f2_1a_ru, K: f2_1a_ru	radio (Matrix)<br>1 Yes<br>0 No
	13	illegal_methadone<br><br>Show the field ONLY if:<br>[country]<>'1'	b. Illegal Methadone U: f2_1b_ru M: N/A, K: f2_1c_ru	radio (Matrix)<br>1 Yes<br>0 No
	14	illegal_bup<br><br>Show the field ONLY if:<br>[country] <> '2'	c. Illegal Buprenorphine/Adnok U: f2_1c_ru M:f2_1b_ru, f2_1b_om, K: N/A	radio (Matrix)<br>1 Yes<br>0 No
	15	morphine	d. Morphine U: f2_1d_ru M:f2_1c_ru, f2_1c_om, K: f2_1d_ru	radio (Matrix)<br>1 Yes<br>0 No
	16	poppy	e. Poppy extract U: f2_1e_ru M: f2_1d_ru, f2_1d_om, K: f2_1e_ru	radio (Matrix)<br>1 Yes<br>0 No
	17	omnopon<br><br>Show the field ONLY if:<br>[country] <> '1'	f. Omnopon U: f2_1f_ru, M: N/A, K: f2_1f_ru	radio (Matrix)<br>1 Yes<br>0 No
	18	codeine<br><br>Show the field ONLY if:<br>[country] <> '1'	g. Codeine U: f2_1g_ru, M: N/A, K: f2_1g_ru	radio (Matrix)<br>1 Yes<br>0 No
	19	promedol<br><br>Show the field ONLY if:<br>[country] <> '1'	h. Promedol U: f2_1h_ru, M: N/A, K:f2_1h_ru	radio (Matrix)<br>1 Yes<br>0 No
	20	fentanyl<br><br>Show the field ONLY if:<br>[country] <> '1'	i. Fentanyl U: f2_1i_ru, M: N/A, K: f2_1i_ru	radio (Matrix)<br>1 Yes<br>0 No
	21	tramadol<br><br>Show the field ONLY if:<br>[country] <> '1'	j. Tramadol U: f2_1j_ru, M: N/A, K: f2_1j_ru	radio (Matrix)<br>1 Yes<br>0 No
	22	other_opioids<br><br>Show the field ONLY if:<br>[country] <> '1'	k. Other opioid analgesics U: f2_1k_ru, M: N/A, K: f2_1j_ru	radio (Matrix)<br>1 Yes<br>0 No
	23	stimulant<br><br>Show the field ONLY if:<br>[country] = '1'	Stimulant, ????? Jef U: Not present M; f2_1e_ru f21e_om	radio (Matrix)<br>1 Yes<br>0 No

	24	khimka<br>Show the field ONLY if: [country] = '1'	Made from marijuana, ?????, 'Khimka' U: not present, M: f2_1f_ru, f2_1f_om	radio (Matrix)<br>1 Yes<br>0 No
	25	dirty_heroin<br>Show the field ONLY if: [country] = '1'	Dirty Heroin, ?????, ?irca U: not present, M: f2_1g_ru, f2_1f_om	radio (Matrix)<br>1 Yes<br>0 No
	26	other_drugs_moldova<br>Show the field ONLY if: [country] = '1'	Other Moldova- Omnopon, Codeine, Promedol U: not present, M: f2_1h_ru, f2_1h_om	radio (Matrix)<br>1 Yes<br>0 No
	27	drug_abuse_score	Part 1 drugs abuse score<br>U: pt1_score_ru<br>M: pt1_score_ru, pt1_score_om<br>K: pt1_score_ru<br>*Count of drugs used in 1a-1k.*	calc<br>Calculation: sum([heroin], [illegal_methadone], [illegal_bup],[morphine], [poppy], [omnopon], [codeine], [promedol], [fentanyl], [tramadol], [other_opioids], [stimulant], [khimka], [dirty_heroin], [other_drugs_moldova])
	28	opioid_tolerance	2. Did you ever have to take more opioids, to get the same effect as you did the first time?<br>U: f2_2_ru<br>M: f2_r_ru, f2_2_om<br>K: f2_2_ru	yesno<br>1 Yes<br>0 No
	29	op_skip_anxiety	3. Did you ever feel anxious at the thought of skipping a dose of opioids?<br>U: f2_3_ru<br>M: f2_3_ru, f2_3_om<br>K: f2_3_ru	yesno<br>1 Yes<br>0 No
	30	opioid_withdrawal	4. Did you ever take opioids in the morning to avoid withdrawal, or did you ever experience withdrawal?<br>U: f2_4_ru<br>M: f2_4_ru, f2_4_om<br>K: f2_4_ru	yesno<br>1 Yes<br>0 No
	31	worry_op_use	5. Do you ever worry about using opioids?<br>U: f2_5_ru<br>M: f2_5_ru, f2_5_om<br>K: f2_5_ru	yesno<br>1 Yes<br>0 No
	32	op_quit_difficulty	6. Was it difficult for you to stop using opioids?<br>U: f2_6_ru<br>M: f2_6_ru, f2_6_om<br>K: f2_6_ru	yesno<br>1 Yes<br>0 No
	33	op_time	7. Did you ever have to spend a lot of time/effort looking for opioids or on recovering after using?<br>U: f2_7_ru<br>M: f2_7_ru, f2_7_om<br>K: f2_7_ru	yesno<br>1 Yes<br>0 No
	34	op_time_wasted	8. Did you ever lose time from important activities , such as visits to the doctor , family / social events or other due to opioids ?<br>U: f2_8_ru<br>M: f2_8_ru, f2_8_om<br>K: f2_8_ru	yesno<br>1 Yes<br>0 No
	35	op_dependence_score	Opioids Dependence Score<br>U: od_score_ru<br>M: od_score_ru, od_score_om<br>K: od_score_ru	calc<br>Calculation: sum([opioid_tolerance], [op_skip_anxiety], [opioid_withdrawal], [worry_op_use], [op_quit_difficulty], [op_time], [op_time_wasted])
	36	elig	Part 2: Eligibility criteria	descriptive
	37	age18	1. 18 and older<br>U: elig1_ru<br>M: elig1_ru, elig1_om<br>K: elig1_ru	yesno<br>1 Yes<br>0 No

	38	received_opioids<br><br>Show the field ONLY if:<br>[country] <> '2'	2. Have you received methadone or burprenorphine in the last 15 days on a gratuitous basis?<br>U: elig2_ru<br>M: elig2_ru, elig2_om<br>K: N/A	radio<br>0 Yes<br>1 No
	39	close_to_facility	3. Lives at most 30 km/60 min from the research facility<br>U: elig3_ru<br>M: elig3_ru, elig3_om<br>K: elig3_ru	radio<br>1 Yes<br>0 No
	40	release_time<br><br>Show the field ONLY if:<br>[country] <> '3'	Time of planned release:<br><br>for men (not less than 7 days to 180 days)<br>for women (at least 7 days to 270 days)<br>U: N/A<br>M: elig4_ru, elig4_om<br>K: elig4_ru	yesno<br>1 Yes<br>0 No
	41	travel_to_site<br><br>Show the field ONLY if:<br>[country] <> '3'	5. Ready to travel to a specific project site after release?<br>U:N/A<br>M: elig5_ru, elig5_om<br>K: elig5_ru	yesno<br>1 Yes<br>0 No
	42	eligibility_score	Eligibility Score<br>U: elig_score_ru eligible if = 3 (doesn't include release_time or travel_to_site)<br>M: elig_score_ru, elig_score_om<br>*Count of criteria met.*	calc<br>Calculation: sum([age18], [received_opioids], [close_to_facility], [release_time], [travel_to_site])
	43	track_marks	Is there evidence of track marks or facility with injection practices?<br><br>U: track_ru	radio<br>1 Yes<br>0 No
	44	index_partner	Is this an Index or Partner participant?	radio<br>1 Index<br>2 Partner
	45	index1<br><br>Show the field ONLY if:<br>[index_partner] = '1'	Is there a person in your network who injects opioids and who may be interested in an opportunity to start OST?	yesno<br>1 Yes<br>0 No
	46	index2<br><br>Show the field ONLY if:<br>[index_partner] = '1'	How many such people do you know?	text (number)
	47	index3a<br><br>Show the field ONLY if:<br>[index_partner] = '1'	Indicate the name/names of opioid dependent person(s)	text, Identifier
	48	index3b<br><br>Show the field ONLY if:<br>[index_partner] = '1'	Indicate the name/names of opioid dependent person(s)	text, Identifier
	49	index3c<br><br>Show the field ONLY if:<br>[index_partner] = '1'	Indicate the name/names of opioid dependent person(s)	text, Identifier
	50	index3d<br><br>Show the field ONLY if:<br>[index_partner] = '1'	Indicate the name/names of opioid dependent person(s)	text, Identifier
	51	index3e<br><br>Show the field ONLY if:<br>[index_partner] = '1'	Indicate the name/names of opioid dependent person(s)	text, Identifier

	52	index4a<br>Show the field ONLY if:<br>[index_partner] = '1'	What is your relationship to [index3a]?	radio<br>1 Spouse<br>2 Sexual Partner<br>3 Mother<br>4 Father<br>5 Sibling<br>6 Other Relative<br>7 Friend<br>8 Other
	53	index4a_other<br>Show the field ONLY if:<br>[index4a] = '8'	Other:	text
	54	index4b<br>Show the field ONLY if:<br>[index_partner] = '1'	What is your relationship to [index3b]?	radio<br>1 Spouse<br>2 Sexual Partner<br>3 Mother<br>4 Father<br>5 Sibling<br>6 Other Relative<br>7 Friend<br>8 Other
	55	index4b_other<br>Show the field ONLY if:<br>[index4b] = '8'	Other:	text
	56	index4c<br>Show the field ONLY if:<br>[index_partner] = '1'	What is your relationship to [index3c]?	radio<br>1 Spouse<br>2 Sexual Partner<br>3 Mother<br>4 Father<br>5 Sibling<br>6 Other Relative<br>7 Friend<br>8 Other
	57	index4c_other<br>Show the field ONLY if:<br>[index4c] = '8'	Other:	text
	58	index4d<br>Show the field ONLY if:<br>[index_partner] = '1'	What is your relationship to [index3d]?	radio<br>1 Spouse<br>2 Sexual Partner<br>3 Mother<br>4 Father<br>5 Sibling<br>6 Other Relative<br>7 Friend<br>8 Other
	59	index4d_other<br>Show the field ONLY if:<br>[index4d] = '8'	Other:	text

	60	index4e<br>Show the field ONLY if:<br>[index_partner] = '1'	What is your relationship to [index3e]?	radio<br>1 Spouse<br>2 Sexual Partner<br>3 Mother<br>4 Father<br>5 Sibling<br>6 Other Relative<br>7 Friend<br>8 Other
	61	index4e_other<br>Show the field ONLY if:<br>[index4e] = '8'	Other:	text
	62	partner1<br>Show the field ONLY if:<br>[index_partner] = '2'	Please indicate the name of the person who told you about this study:	text, Identifier
	63	partner2<br>Show the field ONLY if:<br>[index_partner] = '2'	Indicate the date of birth of the person who told you about the study:	text (date_ymd)
	64	partner3<br>Show the field ONLY if:<br>[index_partner] = '2'	What is the relationship of the person who told you about the study to you?	radio<br>1 Spouse<br>2 Sexual Partner<br>3 Mother<br>4 Father<br>5 Sibling<br>6 Other Relative<br>7 Friend<br>8 Other
	65	form_2_complete	Section Header: *Form Status*<br>Complete?	dropdown<br>0 Incomplete<br>1 Unverified<br>2 Complete
Instrument: **Event** (event)				Collapse
	66	event	Which Event is this?	radio<br>0 Screening<br>1 Day A<br>2 Day B<br>3 Follow-up 1<br>4 Follow-up 2<br>5 Follow-up 3
	67	event_complete	Section Header: *Form Status*<br>Complete?	dropdown<br>0 Incomplete<br>1 Unverified<br>2 Complete
Instrument: **Consent(Kyrgyzstan)** (consentkyrgyzstan)				Collapse
	68	consent<br>Show the field ONLY if:<br>[country] = '2'	Did this participant give consent?<br>K: consent_ru	yesno<br>1 Yes<br>0 No

	69	consentkyrgyzstan_complete	Section Header: *Form Status*<br>Complete?	dropdown<br>0 Incomplete<br>1 Unverified<br>2 Complete
Instrument: **Form 10 And 11** (form_10_and_11) Collapse				
	70	form1011_date	Interview date:<br>U, K: oat_intdt_ru<br>M: oat_intdt_ru, ot_intdt_om	text (date_mdy)
	71	current_on_oat	Is the participant currently on OAT?<br>U, K: onoat_ru<br>M: onoat_ru, onoat_om	radio<br>1 Yes<br>0 No
	72	satisfied_treat_length<br><br>Show the field ONLY if:<br>[current_on_oat] = '1'	1. Are you satisfied with the length of your treatment? (0 = not satisfied, 10 = very satisfied) U, K: f101ru, M: f101ru, f101om	radio (Matrix)<br>0 0<br>1 1<br>2 2<br>3 3<br>4 4<br>5 5<br>6 6<br>7 7<br>8 8<br>9 9<br>10 10
	73	satisfied_dose<br><br>Show the field ONLY if:<br>[current_on_oat] = '1'	2. Are you satisfied with the dose you are receiving? (0 = not satisfied, 10 = very satisfied) U, K: f102ru, M: f102ru, f102om	radio (Matrix)<br>0 0<br>1 1<br>2 2<br>3 3<br>4 4<br>5 5<br>6 6<br>7 7<br>8 8<br>9 9<br>10 10
	74	oat_side_effects<br><br>Show the field ONLY if:<br>[current_on_oat] = '1'	3. How often did you experience side effects from the dose you were taking? (0 = very seldom, 10 = very often) U, K: f103ru, M: f103ru, f103om	radio (Matrix)<br>0 0<br>1 1<br>2 2<br>3 3<br>4 4<br>5 5<br>6 6<br>7 7<br>8 8<br>9 9<br>10 10

	75	oat_effectiveness<br><br>Show the field ONLY if:<br>[current_on_oat] = '1'	4. How effectively does taking methadone or buprenorphine help you reduce other opioid use? (0 = not effective at all, 10 = extremely effective) U, K:f104ru M: f104ru, f104om	radio (Matrix)<br>0 0<br>1 1<br>2 2<br>3 3<br>4 4<br>5 5<br>6 6<br>7 7<br>8 8<br>9 9<br>10 10
	76	oat_qol_increase<br><br>Show the field ONLY if:<br>[current_on_oat] = '1'	5. How much does treatment with methadone/buprenorphine increase your quality of life? (0 = doesn't help at all, 10 = helps a lot) U, K: f105ru, M: f105ru, f105om	radio (Matrix)<br>0 0<br>1 1<br>2 2<br>3 3<br>4 4<br>5 5<br>6 6<br>7 7<br>8 8<br>9 9<br>10 10
	77	oat_relations_improve<br><br>Show the field ONLY if:<br>[current_on_oat] = '1'	6. How much does treatment with methadone or buprenorphine affect your relationships with friends and family? (0 = doesn't help at all, 10 = helps a lot) U, K:f106ru, M: f106ru, f106om	radio (Matrix)<br>0 0<br>1 1<br>2 2<br>3 3<br>4 4<br>5 5<br>6 6<br>7 7<br>8 8<br>9 9<br>10 10
	78	interest_in_oat<br><br>Show the field ONLY if:<br>[current_on_oat] = '0'	1. How interested are you in receiving OAT? (0 = not interested at all, 10 = very interested) U, K:f11_1_ru, M: f11_1_ru, f11_1_om	radio (Matrix)<br>0 0<br>1 1<br>2 2<br>3 3<br>4 4<br>5 5<br>6 6<br>7 7<br>8 8<br>9 9<br>10 10

	79	difficulty_start_oat<br><br>Show the field ONLY if:<br>[current_on_oat] = '0'	2. You said you were interested in receiving OAT. How do difficult do you think it would be for you to start treatment? (0 = not difficult at all, 10 = very difficult) U, K: f11_2_ru, M: f11_2_ru, f11_2_om	radio (Matrix)<br>0 0<br>1 1<br>2 2<br>3 3<br>4 4<br>5 5<br>6 6<br>7 7<br>8 8<br>9 9<br>10 10
	80	importance_receive_oat<br><br>Show the field ONLY if:<br>[current_on_oat] = '0'	3. How important is it for you to receive OAT? (0 = not important at all, 10 = very important) U, K:f11_3_ru, M: f11_3_ru, f11_3_om	radio (Matrix)<br>0 0<br>1 1<br>2 2<br>3 3<br>4 4<br>5 5<br>6 6<br>7 7<br>8 8<br>9 9<br>10 10
	81	so1<br><br>Show the field ONLY if:<br>[current_on_oat] = '0'	Section Header: *Motivation Socrates 8D Please read the following statements carefully. Each one describes a way that you might (or might not) feel about your drug use. For each statement, select one number from 1 to 5, to indicate how much you agree or disagree with it right now. Please select one and only one number for every statement. U, K: so1ru-so19ru, M: so1ru-so19ru, so1om-so19om*<br><br>1. I really want to make changes in my use of drugs.	radio (Matrix)<br>1 1 Absolutely do not agree<br>2 2<br>3 3<br>4 4<br>5 5 Absolutely agree
	82	so2<br><br>Show the field ONLY if:<br>[current_on_oat] = '0'	2. Sometimes I wonder if I am an addict.	radio (Matrix)<br>1 1 Absolutely do not agree<br>2 2<br>3 3<br>4 4<br>5 5 Absolutely agree
	83	so3<br><br>Show the field ONLY if:<br>[current_on_oat] = '0'	3. If I don't change my drug use soon, my problems are going to get worse.	radio (Matrix)<br>1 1 Absolutely do not agree<br>2 2<br>3 3<br>4 4<br>5 5 Absolutely agree
	84	so4<br><br>Show the field ONLY if:<br>[current_on_oat] = '0'	4. I have already started making some changes in my use of drugs.	radio (Matrix)<br>1 1 Absolutely do not agree<br>2 2<br>3 3<br>4 4<br>5 5 Absolutely agree

	85	so5<br><br>Show the field ONLY if:<br>[current_on_oat] = '0'	5. I was using drugs too much at one time, but I've managed to change that.	radio (Matrix)<br>1 | 1 Absolutely do not agree<br>2 | 2<br>3 | 3<br>4 | 4<br>5 | 5 Absolutely agree
	86	so6<br><br>Show the field ONLY if:<br>[current_on_oat] = '0'	6. Sometimes I wonder if my drug use is hurting other people.	radio (Matrix)<br>1 | 1 Absolutely do not agree<br>2 | 2<br>3 | 3<br>4 | 4<br>5 | 5 Absolutely agree
	87	so7<br><br>Show the field ONLY if:<br>[current_on_oat] = '0'	7. I have a drug problem.	radio (Matrix)<br>1 | 1 Absolutely do not agree<br>2 | 2<br>3 | 3<br>4 | 4<br>5 | 5 Absolutely agree
	88	so8<br><br>Show the field ONLY if:<br>[current_on_oat] = '0'	8. I'm not just thinking about changing my drug use, I'm already doing something about it.	radio (Matrix)<br>1 | 1 Absolutely do not agree<br>2 | 2<br>3 | 3<br>4 | 4<br>5 | 5 Absolutely agree
	89	so9<br><br>Show the field ONLY if:<br>[current_on_oat] = '0'	9. I have already changed my drug use, and I am looking for ways to keep from slipping back to my old pattern.	radio (Matrix)<br>1 | 1 Absolutely do not agree<br>2 | 2<br>3 | 3<br>4 | 4<br>5 | 5 Absolutely agree
	90	so10<br><br>Show the field ONLY if:<br>[current_on_oat] = '0'	10. I have serious problems with drugs.	radio (Matrix)<br>1 | 1 Absolutely do not agree<br>2 | 2<br>3 | 3<br>4 | 4<br>5 | 5 Absolutely agree
	91	so11<br><br>Show the field ONLY if:<br>[current_on_oat] = '0'	11. Sometimes I wonder if I am in control of my drug use.	radio (Matrix)<br>1 | 1 Absolutely do not agree<br>2 | 2<br>3 | 3<br>4 | 4<br>5 | 5 Absolutely agree
	92	so12<br><br>Show the field ONLY if:<br>[current_on_oat] = '0'	12. My drug use is causing a lot of harm.	radio (Matrix)<br>1 | 1 Absolutely do not agree<br>2 | 2<br>3 | 3<br>4 | 4<br>5 | 5 Absolutely agree

	93	so13<br><br>Show the field ONLY if:<br>[current_on_oat] = '0'	13. I am actively doing things now to cut down or stop my use of drugs.	radio (Matrix)<br>1 1 Absolutely do not agree<br>2 2<br>3 3<br>4 4<br>5 5 Absolutely agree
	94	so14<br><br>Show the field ONLY if:<br>[current_on_oat] = '0'	14. I want help to keep from going back to the drug problems that I had before.	radio (Matrix)<br>1 1 Absolutely do not agree<br>2 2<br>3 3<br>4 4<br>5 5 Absolutely agree
	95	so15<br><br>Show the field ONLY if:<br>[current_on_oat] = '0'	15. I know that I have a drug problem.	radio (Matrix)<br>1 1 Absolutely do not agree<br>2 2<br>3 3<br>4 4<br>5 5 Absolutely agree
	96	so16<br><br>Show the field ONLY if:<br>[current_on_oat] = '0'	16. There are times when I wonder if I use drugs too much.	radio (Matrix)<br>1 1 Absolutely do not agree<br>2 2<br>3 3<br>4 4<br>5 5 Absolutely agree
	97	so17<br><br>Show the field ONLY if:<br>[current_on_oat] = '0'	17. I am a drug addict.	radio (Matrix)<br>1 1 Absolutely do not agree<br>2 2<br>3 3<br>4 4<br>5 5 Absolutely agree
	98	so18<br><br>Show the field ONLY if:<br>[current_on_oat] = '0'	18. I am working hard to change my drug use.	radio (Matrix)<br>1 1 Absolutely do not agree<br>2 2<br>3 3<br>4 4<br>5 5 Absolutely agree
	99	so19<br><br>Show the field ONLY if:<br>[current_on_oat] = '0'	19. I have made some changes in my drug use, and I want some help to keep from going back to the way I used before.	radio (Matrix)<br>1 1 Absolutely do not agree<br>2 2<br>3 3<br>4 4<br>5 5 Absolutely agree
	100	form_10_and_11_complete	Section Header: *Form Status*<br><br>Complete?	dropdown<br>0 Incomplete<br>1 Unverified<br>2 Complete

Instrument: **Demographics** (demographics) Collapse

	101	interviewer_id_demo	Interviewer ID<br>U: dm_intidru, dm_intidruf1<br>K: dm_intru<br>M: N/A	text
	102	date_demo	Demographics Interview Date<br>not in U<br>K: dm_dtru<br>M: N/A	text
	103	gender<br><br>Show the field ONLY if:<br>[event] = '1'	1. How would you best identify your gender<br>U, K: dm1ru<br>M: dm1ru, dm1om	radio<br>1 Male<br>2 Female
	104	dob<br><br>Show the field ONLY if:<br>[event] = '1'	2. Please, provide your date birth:<br>U, K: dm2ru<br>M: dm2ru, dm2om<br>*Use 01 for day. Enter mm/01/yyyy for example 11/01/1961*	text (date_mdy, Min: 1917-01-01, Max: 1999-01-01), Identifier
	105	ethnicity<br><br>Show the field ONLY if:<br>[event] = '1'	3. Ethnicity<br>U, K: dm3ru<br>M: dm3ru, dm3om	radio<br>1 Kyrgyz<br>2 Moldovan<br>3 Romanian<br>4 Romani<br>5 Russian<br>6 Ukrainian<br>7 Uzbek<br>8 Jewish<br>9 Gagauz<br>10 Other - specify below
	106	ethnicity_other<br><br>Show the field ONLY if:<br>[ethnicity] = '10'	3a. Other Ethnicity<br>U, K: dm3aru<br>M: dm3aru, dm3aom	text
	107	religion_importance<br><br>Show the field ONLY if:<br>[event] = '1'	5. On a scale from 0 to 10, how important is the role of your religion in your life?<br>U, K: dm5ru<br>M: dm5ru, dm5om	radio<br>0 0- not important at all<br>1 1<br>2 2<br>3 3<br>4 4<br>5 5<br>6 6<br>7 7<br>8 8<br>9 9<br>10 10 - very important
	108	marital_status<br><br>Show the field ONLY if:<br>[event] = '1'	6. How would you describe your current marital/relationship status: [choose only one]<br>U, K: dm6ru<br>M: dm6ru, dm6om	radio<br>1 Single, never married<br>2 Married<br>3 Not married but in a long term relationship<br>4 Separated /Divorced<br>5 Widow/widower
	109	children<br><br>Show the field ONLY if:<br>[event] = '1'	7. How many total children do you have?<br>U, K: dm7ru<br>M: dm7ru, : dm7om	text (number, Min: 0, Max: 20)<br>Custom alignment: LH

	110	young_children<br>Show the field ONLY if:<br>[event] = '1' AND [children] > 0	8. How many of these children are younger than 18 years old?<br>U, K: dm8ru<br>M: dm8ru, dm8om	text (number, Min: 0, Max: 20)<br>Custom alignment: LH
	111	education<br>Show the field ONLY if:<br>[event] = '1'	9. Highest level of education completed:<br>U,K: dm9ru<br>M: dm9ru, dm9om	radio<br>1 No education<br>2 Completed primary school (grades 1-4)<br>3 Incomplete high school education (grades 5-9)<br>4 Completed high school education (grades 10-12)<br>5 Completed high school-vocational education (technical college)<br>6 Incomplete university education (completed third year)<br>7 Completed university
	112	housing	10. U: Which is the best description of your living situation (where do you live) ? K, M: Where are you going to live?<br>U, K: dm10ru, dm10ruf1<br>M: dm10ru, dm10om	radio<br>1 My own place that I own<br>2 Apartment/house that I rent<br>3 My parents/relatives place<br>4 My friend's place (include boyfriend/girlfriend)<br>5 Dormitory/hotel (temporary housing)<br>6 Homeless shelter<br>7 Homeless<br>8 Don't know<br>9 Other (Specify other place where you live below)
	113	housing_other<br>Show the field ONLY if:<br>[housing] = '9'	10a. Other (Specify other place where you live):<br>U,K: dm10aru, dm10aruf1<br>M: dm10aru, dm10aom	text
	114	housing_security	11. How sure are you that you will be able to remain at this place to live (scale from 1: very unsure to 5: very sure)?<br>U, K: dm11ru, dm11ruf1<br>M: dm11ru, dm11om	radio<br>1 1 (Very unsure)<br>2 2<br>3 3<br>4 4<br>5 5 (Very sure)
	115	housing_alone	12. U: Do you currently live alone? K, M: Are you going to live alone?<br>U, K: dm12ru, dm12ruf1<br>M: dm12ru, dm12om	radio<br>1 Yes<br>0 No
	116	housing_with<br>Show the field ONLY if:<br>[housing_alone] = '0'	13. Who currently lives in there?<br>U, K: dm13ru, dm13ruf1<br>M: dm13ru, dm13om	checkbox<br>a housing_with___a Spouse<br>b housing_with___b Children<br>c housing_with___c Unmarried sexual partner<br>d housing_with___d Mother<br>e housing_with___e Father<br>f housing_with___f Sibling<br>g housing_with___g Extended family member<br>h housing_with___h Spouse's family<br>i housing_with___i Friends<br>j housing_with___j Other, specify below
	117	housing_with_other<br>Show the field ONLY if:<br>[housing_with(j)] = '1'	13a. Other person in household specify<br>U, K: dm13aru, dm13aruf1<br>M: dm13aru, dm13aom	text

	118	housing_time<br>Show the field ONLY if:<br>[event] = '1'	14. How long have you lived in this place?<br><br>U: dm14ru<br>K, M: N/A	radio<br>1 My entire life<br>2 Most of my life<br>3 A few years<br>4 A few months<br>5 A few days
	119	employment	15. How would you describe your CURRENT employment situation?<br>U: dm15ru, dm15ruf1<br>K, M: N/A	radio<br>1 Full time<br>2 Part time<br>3 Temporary / Seasonal<br>4 Day laborer<br>5 Not employed
	120	employment_2	15. How would you describe your CURRENT employment situation?<br>K : dm15f1___1: dm15f1___5	checkbox<br>1 employment_2___1 Full time<br>2 employment_2___2 Part time<br>3 employment_2___3 Temporary / Seasonal<br>4 employment_2___4 Day laborer<br>5 employment_2___5 Not employed
	121	income	16. What is your total monthly income from all the sources (official and unofficial) in American Dollars?<br>U, K: dm16ru, dm16ruf1<br>M: dm16ru, dm16om	text (number, Min: 0, Max: 25000)
	122	income_leasing	Section Header: *DM17. Do any of the following additional sources contribute to your "TOTAL" monthly income?*<br>Leasing (apartment, car, etc.) U, K:dm17a1ru dm17a1ruf1, M: dm17a1ru, dm17aom	radio (Matrix)<br>1 Yes<br>0 No
	123	income_social_pay	Social payments (pension, allowance, etc.) U, K:dm17bru dm17bruf1 M: dm17bru, dm17bom	radio (Matrix)<br>1 Yes<br>0 No
	124	income_goods_money	Exchange goods for money U, K: dm17cru dm17cruf1, M: dm17cru, dm17com	radio (Matrix)<br>1 Yes<br>0 No
	125	official_pris_work	Official work in prison U: N/A, K: dm17dru, M: dm17dru, dm17dom	radio (Matrix)<br>1 Yes<br>0 No
	126	unofficial_pris_work	Unofficial work in prison U: N/A, K: dm17eru, M: dm17eru, dm17eom	radio (Matrix)<br>1 Yes<br>0 No
	127	income_goods_goods	Exchange goods for goods U, K: dm17fru dm17fruf1, M: dm17fru, dm17fom	radio (Matrix)<br>1 Yes<br>0 No
	128	income_relatives	Relatives support U, K: dm17gru dm17gruf1, M: dm17gru, dm17gom	radio (Matrix)<br>1 Yes<br>0 No
	129	income_other	Other U, K: dm17hru dm17hruf1, M: dm17hru, dm17hom	radio (Matrix)<br>1 Yes<br>0 No
	130	income_other_describe	17 Other<br>U, K: dm17othru dm17othruf1<br>M: dm17othru, dm17othom	text

	131	passport_yn	18b. Do you have a passport?<br>U, K: dm18bru dm18bruf1<br>M: dm18bru, dm18bom	radio<br>1 Yes<br>0 No<br>7 Other
	132	passport_other<br><br>Show the field ONLY if:<br>[passport_yn] = '7'	Describe your passport situation<br>U: dm18bothru<br>K: N/A	text
	133	id_card_yn	19. Do you have an ID number/card<br>U, K: dm19ru dm19ruf1<br>M: dm19ru, dm19om	radio<br>1 Yes<br>0 No
	134	demographics_complete	Section Header: *Form Status*<br>Complete?	dropdown<br>0 Incomplete<br>1 Unverified<br>2 Complete
Instrument: **Criminal Justice History** (criminal_justice_history)				Collapse
	135	cjhlabel	The next series of questions are going to ask you about history with law enforcement. This information will be used only by the research team, please, be honest as possible.	descriptive
	136	cjh_interviewer	interviewer ID<br>K: cjh_intru<br>M: N/A	text
	137	cjh_interview_date	Interview Date<br>K: cjh_dtru<br>M: N/A	text (date_mdy)
	138	cjh1	1. Have you ever been in pre-trial detention centers (SIZO)?<br>U, K: cjh1ru<br>M: cjh1ru, cjh1om	yesno<br>1 Yes<br>0 No
	139	cjh2<br><br>Show the field ONLY if:<br>[cjh1] = '1'	2. How many total times have you been in pre-trial detention centers (SIZO)?<br><br>U, K:cjh2ru<br>M: cjh2ru, cjh2om<br>*times*	text (number)
	140	cjh3y<br><br>Show the field ONLY if:<br>[cjh1] = '1'	3. How many years, months, and days did you spend in a pre-trial detention center (SIZO) last time you were in SIZO? Enter "0" for zero months or days.<br>Years<br>U, K:cjh3yru<br>M:cjh3yru, cjh3yom<br>*Years*	text (integer)
	141	cjh3m<br><br>Show the field ONLY if:<br>[cjh1] = '1'	3. Months<br>U, K:cjh3mru<br>M: cjh3mru, cjh3mom<br>*Months*	text (integer, Min: 0, Max: 12)
	142	cjh3d<br><br>Show the field ONLY if:<br>[cjh1] = '1'	3. Days<br>U, K: cjh3dru<br>M: cjh3dru, cjh3dom<br>*Days*	text (integer, Min: 0, Max: 30)
	143	cjh4	4. During the LATEST time in SIZO, did you experience symptoms of abstinence syndrome?<br><br>U; cjh4ru<br>K, M: N/A	yesno<br>1 Yes<br>0 No
	144	cjh4a	4a.Have you ever been in prison (NOT including SIZO)?<br><br>U;cjh4a_proru<br>K, M: N/A	yesno<br>1 Yes<br>0 No

	145	cjh5<br><br>Show the field ONLY if:<br>[cjh4a] = '1'	5. How old were you at the time of your first imprisonment (NOT including SIZO)?<br>U, K:cjh5ru<br>M: cjh5ru, cjh5om	text (number, Min: 14, Max: 90)
	146	cjh6<br><br>Show the field ONLY if:<br>[cjh4a] = '1'	6. Approximately how many times have you been in prison (not including SIZO)?<br>U, K:cjh6ru<br>M: cjh6ru. cjh6om	text (number, Min: 0)
	147	cjh7y<br><br>Show the field ONLY if:<br>[cjh4a] = '1'	7. How many total years, months have you spent in prison (not including SIZO) throughout your life?<br>Years<br>U, K:cjh7yru<br>M: cjh7yom<br>*Years*	text (number)
	148	cjh7m<br><br>Show the field ONLY if:<br>[cjh4a] = '1'	7. Months<br>U, K:cjh7mru<br>M cjh7mru, cjh7mom<br>*Months*	text (number, Min: 0, Max: 12)
	149	cjh7d	7. Days<br>U: N/A<br>K: cjh7dru<br>M: cjh7dru, cjh7dom	text (number, Min: 0, Max: 30)
	150	cjh7ay	7a. When did you enter prison this time? Year<br><br>U: N/A<br>K: cjh7aru<br>M: cjh7aom	text (number, Min: 1970, Max: 2018)
	151	cjh7am	7a. Month<br><br>U: N/A<br>K: cjh7amru<br>M: cjh7amom	text (number, Min: 0, Max: 12)
	152	cjh8	8. Type of offence(s) for the last incarceration (check all that apply):<br>U, K:cjh8ru<br>M: cjh8om	checkbox<br>1 cjh8___1 Homicide<br>2 cjh8___2 Violent-related crime<br>3 cjh8___3 Property-related crime<br>4 cjh8___4 Substance-related offence<br>5 cjh8___5 Sexual-related crime<br>6 cjh8___6 Fraud<br>7 cjh8___7 Other
	153	cjh8oth<br><br>Show the field ONLY if:<br>[cjh8(7)] = '1'	8. Other<br>U, K:cjh8othru<br>M: cjh8othom	text
	154	cjh9<br><br>Show the field ONLY if:<br>[screen_arm_1][country] = '2'	9. Which caste within prison do people of your group pertain to?<br>U: N/A<br>K: cjh9ru<br>M: cjh9ru. cjh9om	checkbox<br>1 cjh9___1 ???????/blatnye<br>2 cjh9___2 ??????????/poriadochnye<br>3 cjh9___3 ????/gady<br>4 cjh9___4 ?????????/obizehnnye<br>5 cjh9___5 ??????/ other
	155	cjh9a	9a. Other;<br>U: N/A<br>K: cjh9othru<br>M: cjh9othru, cjh9othom	text
	156	criminal_justice_history_complete	Section Header: *Form Status*<br>Complete?	dropdown<br>0 Incomplete<br>1 Unverified<br>2 Complete

Instrument: **Form 12 Lab Results** (form_12_lab_results)

Collapse

	157	test_date	Date of lab testing	text (date_mdy), Required
	158	log_date	Form Submission date	text (date_mdy)
	159	f12_interviewer_id	Form 12 interviewer ID	text
	160	pre_test_done	Section Header: *HIV Infection*<br>HIV pre-test consultation was done:	yesno, Required<br>1 Yes<br>0 No
	161	pre_test_reason<br>Show the field ONLY if:<br>[pre_test_done] = '2'	If the pre-test consultation was not done, write the reason here;	text
	162	date_res_given	Date results were given:	text (date_mdy)
	163	post_test	Was the post-test consulatation done?	yesno, Required<br>1 Yes<br>0 No
	164	post_test_reason<br>Show the field ONLY if:<br>[post_test] = '2'	Give the reason why the post-test consultation was not done.	text
	165	result_hiv	Section Header: *Test Results*<br>HIV	radio<br>1 Preliminary positive<br>0 Preliminary negative
	166	hiv_pos<br>Show the field ONLY if:<br>[result_hiv] = '1'	Recommended - to take confirmatory test:	yesno<br>1 Yes<br>0 No
	167	hiv_neg<br>Show the field ONLY if:<br>[result_hiv] = '2'	Recommended - in case of risky behaviors and taken into account the "window" repeat the test after 3 months:	yesno<br>1 Yes<br>0 No
	168	result_hbv	HBV Result:	radio, Required<br>1 Preliminary positive<br>0 Preliminary negative
	169	hbv_pos<br>Show the field ONLY if:<br>[result_hbv] = '1'	HBV Preliminary positive-Recommended - to take confirmatory test	yesno<br>1 Yes<br>0 No
	170	result_hcv	HCV test result	radio, Required<br>1 Preliminary positive<br>0 Preliminary negative
	171	hcv_pos<br>Show the field ONLY if:<br>[result_hbv] = '1'	Preiliminary positive HCV: Recommended - to take confirmatory test:	yesno<br>1 Yes<br>0 No
	172	result_syphilis	Syphilis Test Result	radio, Required<br>1 Preliminary positive<br>0 Preliminary negative
	173	syph_pos<br>Show the field ONLY if:<br>[result_hbv] = '1'	Preliminary positive for syphilis, recommended - to take confirmatory test	yesno<br>1 Yes<br>0 No
	174	form_12_lab_results_complet e	Section Header: *Form Status*<br>Complete?	dropdown<br>0 Incomplete<br>1 Unverified<br>2 Complete

Instrument: **Age At Drug Initiation** (age_at_drug_initiation)

	175	adi_interviewer_id	Interviewer ID<br>U: N/A<br>K: adintru	text
	176	adi_date	Interview Date<br>U: N/A<br>K:addtru	text (date_mdy)
	177	drug_start_age	1. How old were you the first time that you used drugs, but did NOT inject them?<br><br>Ukraine: Hidden ad1ru<br>K: N/A<br>*Put 0 if you have never used non injecting drugs*	text (number, Min: 0, Max: 99)
	178	inject_drug_start_age	2. How old were you the first time you injected drugs, that is put a needle in your arm?<br>U,K: ad2ru<br>*Put 0 if you have never injected drugs*	text (number, Min: 0, Max: 99)
	179	inject_opioid_start_age	3. How old were you the first time you injected any kind of opioid (shirka, heroin, desomorphine, adnok/buprenorphine, methadone, extract of poppy straw, acetylated extract of poppy straw, chernaya, medical opiates: morphine, opium, omnopon, promedol)<br><br>U, K: ad3ru<br>*Put 0 if you have never injected opioid drugs*	text (number, Min: 0, Max: 99)
	180	age_at_drug_initiation_complete	Section Header: *Form Status*<br>Complete?	dropdown<br>0 Incomplete<br>1 Unverified<br>2 Complete

Instrument: **Dast10** (dast10)

	181	dast_interviewer_id	Interviewer ID	text
	182	dast_date	Interview Date	text (date_mdy)
	183	dast	The next ten questions have to do with your use of drugs. Please answer Yes or No to each of these questions.<br><br>In the 12 months before this incarceration:	descriptive
	184	dast1	1. Have you used drugs other than those required for medical reasons?	radio (Matrix)<br>1 Yes<br>0 No
	185	dast2	2. Do you abuse more than one drug at a time?	radio (Matrix)<br>1 Yes<br>0 No
	186	dast4	3. Have you ever had "blackouts" or "flashbacks" as a result of drug use?	radio (Matrix)<br>1 Yes<br>0 No
	187	dast5	4. Do you ever feel bad or guilty about your drug use?	radio (Matrix)<br>1 Yes<br>0 No
	188	dast6	5. Does your spouse (or parent) ever complain about your involvement with drugs?	radio (Matrix)<br>1 Yes<br>0 No
	189	dast7	6. Have you neglected your family because of your use of drugs?	radio (Matrix)<br>1 Yes<br>0 No

	190	dast8	7. Have you engaged in illegal activities in order to obtain drugs?	radio (Matrix)<br>1 Yes<br>0 No
	191	dast9	8. Have you ever experienced withdrawal symptoms (i.e. felt sick) when you stopped taking drugs?	radio (Matrix)<br>1 Yes<br>0 No
	192	dast10	9. Have you had medical problems as a result of your drug use (e.g., memory loss, hepatitis, convulsions, bleeding etc.)?	radio (Matrix)<br>1 Yes<br>0 No
	193	dast3	10. Are you unable to stop using drugs when you want to?	radio<br>0 Yes<br>1 No<br><br>Custom alignment: RH
	194	dast_sum	Score 1 point for each question answered, "YES", except for question (3) for which a "NO" answer receives 1 point and a "YES" answer receives 0 points.	calc<br>Calculation: ([dast1] + [dast2] + [dast3] + [dast4] + [dast5] + [dast6] + [dast7] + [dast8] + [dast9] + [dast10])
	195	dast_score	DAST-10 Degree of Problem Suggested Score Related to Drug Abuse Action	calc<br>Calculation: if([dast_sum]=0, 1, if([dast_sum]=1, 2, if([dast_sum]=2, 2, if([dast_sum]=3, 3, if([dast_sum]=4, 3, if([dast_sum]=5, 3, if([dast_sum]=6, 4, if([dast_sum]=7, 4, if([dast_sum]=8, 4, if([dast_sum]=9, 5, if([dast_sum]=10, 5, ''))))))))))
	196	dast10_complete	Section Header: *Form Status*<br>Complete?	dropdown<br>0 Incomplete<br>1 Unverified<br>2 Complete
Instrument: **Drug Risk Behaviors** (drug_risk_behaviors)				Collapse
	197	rbd_interviewer_id	Interviewer ID	text
	198	rbd_date	Interview Date	text (date_mdy)
	199	rbd1	1. Have you ever injected drugs even once?<br>K:rbd1ru	radio<br>1 Yes<br>0 No
	200	rbd1a<br><br>Show the field ONLY if:<br>[rbd1] = '1'	1a. Have you ever injected drugs within prison or sizo?<br>K:rbd1aru	radio<br>1 Yes<br>0 No
	201	rbd1b<br><br>Show the field ONLY if:<br>[rbd1] = '1'	1b. Your first experience injecting was in:<br>K:rbd1bru	radio<br>1 Community<br>2 Prison<br>3 Sizo
	202	rbd1c<br><br>Show the field ONLY if:<br>[rbd1b] = '2' or [rbd1b] = '3'	1c. How old were you the FIRST time you injected drugs in prison or sizo?<br>K:rbd1cru	text (integer, Min: 12, Max: 98)
	203	rbd1d<br><br>Show the field ONLY if:<br>[rbd1b] = '2' or [rbd1b] = '3'	1d. How old were you the LAST time you injected drugs in prison or sizo? K:rbd1dru	text (integer, Min: 12, Max: 98)
	204	rbd1e<br><br>Show the field ONLY if:<br>[rbd1b] = '2' and [rbd1b] = '3'	1e. When you injected drugs in prison or sizo, how many people usually shared the syringe or needle you used (provide best estimate)? K:rbd1eru	text (integer, Min: 0, Max: 20)

	205	rbdp3<br><br>Show the field ONLY if:<br>[screen_arm_1][country]= '2'	p3. Have you ever used heroin that was given out during razgon? K: rbdp3_kyr	yesno<br>1 Yes<br>0 No
	206	rbdp4<br><br>Show the field ONLY if:<br>[screen_arm_1][country]= '2'	p4. How often did you use heroin that was given out during razgon in the past six months? K: rbdp3_kyr	text (number, Min: 0, Max: 300)
	207	rbd13a<br><br>Show the field ONLY if:<br>[rbd1] = '1'	13a. Thinking of the times you did not use a clean needle in the past month, how much trouble would it have been to get a new one? Would it have been... K:rbd13a	radio<br>0 Very Difficult<br>1 Difficult<br>2 Somewhat Difficult<br>3 Easy<br>4 Very Easy<br>8 Refuse to Answer<br>9 Not Applicable
	208	rbd14<br><br>Show the field ONLY if:<br>[rbd1] = '1'	14. Have you received needles or syringes from needle exchange points?	radio<br>1 Yes<br>0 No
	209	rbd14a<br><br>Show the field ONLY if:<br>[rbd1] = '1' and [screen_arm_1][country] = '2'	14a. Have you received needles or syringes from the needle exchange point in a prison facility during this incarceration?<br><br>U: N/A K: rbd14aa_ky	yesno<br>1 Yes<br>0 No
	210	rbdp18<br><br>Show the field ONLY if:<br>[rbd14a] = '0'	p18. Why did you not use the services of the needle/syringe exchange point during your incarceration? K: rbdp18<br><br>U: N/A	checkbox<br>1 rbdp18___1 I don't need them<br>2 rbdp18___2 I did not want to disclose I am injecting<br>3 rbdp18___3 I could get syringes from a friend<br>4 rbdp18___4 I am not registered at the syringe exchange point<br>5 rbdp18___5 Medical staff did not give them to me<br>6 rbdp18___6 I already had a syringe<br>7 rbdp18___7 Other:_________
	211	rbdp18_other<br><br>Show the field ONLY if:<br>[rbdp18(7)] = '1'	p18 Other:________<br><br>U: N/A	text
	212	rbd19<br><br>Show the field ONLY if:<br>[rbd1] = '1'	19. In your lifetime, have you ever split the drug solution in a syringe with other drug users?	radio<br>1 Yes<br>0 No
	213	rbdp21<br><br>Show the field ONLY if:<br>[rbd1] = '1'	p21. How many times in the last 30 days did you front or backload (split the drug solution in a syringe with others)?<br>*888 Refuse to Answer*	text (number, Min: 0, Max: 888)
	214	drug_risk_behaviors_complete	Section Header: *Form Status*<br>Complete?	dropdown<br>0 Incomplete<br>1 Unverified<br>2 Complete
Instrument: **Inventory Of Drug Use** (inventory_of_drug_use)				Collapse
	215	idu_interviewer_id	Interviewer ID:<br><br>K:diintru	text

	216	idu_date	Interview Date:<br><br>K:didtru	text (date_mdy)
	217	di2a<br><br>Show the field ONLY if:<br>[event] = '1'	2a. Have you ever used opioids (i.e. liquid or powered heroin, illegal/street methadone, medical opioids, extract of poppy straw ', shirka', 'hanka', 'himka')<br>U, K:di2aru<br>M: di2aru, di2aom<br><br>CODE NO TO '0'	radio<br>1 Yes<br>0 No
	218	di2c<br><br>Show the field ONLY if:<br>[event] = '1' and [di2a] = '1'	2c. In the last 30 days, how many days did you inject opioids(in the form of liquid or powder, including heroin, street methadone, medical opioids, extract of poppy straw', shirka', 'hanka', 'himka')?<br><br>U:di2cru, M:di2druf1	text (integer, Min: 0, Max: 30)
	219	di2c_abbrev<br><br>Show the field ONLY if:<br>[event] = '3' or [event] = '4' or [event] = '5' and [country] = '3'	2c. In the last 30 days, how many days did you inject heroin?<br><br>U:di2cruf1	text (integer, Min: 0, Max: 30)
	220	di2d<br><br>Show the field ONLY if:<br>[di2a] = '1' and [screen_arm_1][country] <> '3'	2d. In the 30 days JUST BEFORE THIS INCARCERATION, how many days did you inject opioids?<br><br>U: N/A<br>K: di2dru<br>M: di2dru, di2dom	text (integer, Min: 0, Max: 30)
	221	di3c<br><br>Show the field ONLY if:<br>[event] = '3' or [event] = '4' or [event] = '5' and [country] = '3'	3c. In the last 30 days, how many days did you use illegal/ street methadone?<br><br>U:di3cruf1	text (integer, Min: 0, Max: 30)
	222	di3d<br><br>Show the field ONLY if:<br>[event] = '3' or [event] = '4' or [event] = '5' and [country] = '3'	3d. How many days have you used opioids (medical opioids, poppy straw extract, 'shirka', 'hanka' , 'himka')?<br><br>U:di3druf1	text (integer, Min: 0, Max: 30)
	223	di6c<br><br>Show the field ONLY if:<br>[event] = '3' or [event] = '4' or [event] = '5' and [country] = '3'	6c. In the last 30 days, how many days did you use opioids(medical opioids, extract of poppy straw', shirka', 'hanka', 'himka')?<br><br>U:di6cruf1	text (integer, Min: 0, Max: 30)
	224	di6d<br><br>Show the field ONLY if:<br>[event] = '3' or [event] = '4' or [event] = '5' and [country] = '3'	6d. In the last 30 days, how many days did you inject opioids(medical opioids, extract of poppy straw', shirka', 'hanka', 'himka')?<br><br>U:di6druf1	text (integer, Min: 0, Max: 30)
	225	di6aa<br><br>Show the field ONLY if:<br>[event] = '1'	6aa. Have you ever used DIMEDROL or SOMNOL concurrently with OPIOIDS (heroin, street/illegal methadone, medical opioids, or homemade opioids)?<br><br>U, K: di6aaru<br>M: di6aaru, di26aaom<br><br>Recode 'no' from 2 to 0	radio<br>1 Yes<br>0 No
	226	di6bb<br><br>Show the field ONLY if:<br>[event] = '1' AND [di6bb] = '1'	6bb. Where have you used DIMEDROL or SOMNOL concurrently with OPIOIDS (heroin, street/illegal methadone, medical opioids, or homemade opioids)? (check everything that applies)<br><br>U, K: di6bbru<br>M: di6bbru, di6bbom	checkbox<br>1 di6bb___1 in prison<br>2 di6bb___2 in SIZO<br>3 di6bb___3 in the community

	227	di6dd<br><br>Show the field ONLY if:<br>[event] = '3' or [event] = '4' or [event] = '5' or ([di6bb] = '1' and [screen_arm_1][country] < > '3')	6dd. How many days out of the last 30 days have you used DIMEDROL or SOMNOL concurrently with OPIOIDS (heroin, street/illegal methadone, medical opioids, or homemade opioids)?<br><br>U:di6ddru, di6ddruf1 M: di6eeruf1<br>*Days*	text (integer, Min: 0, Max: 30)
	228	di6ee<br><br>Show the field ONLY if:<br>[di6bb] = '1' and [event] = '1'	6ee. In the 30 DAYS BEFORE THIS INCARCERATON, how many days did you use DIMEDROL or SOMNOL concurrently with OPIOIDS (heroin, street/illegal methadone, medical opioids, or homemade opioids)?<br>U: N/A<br>K:di6eeru<br>M. M: di6eeru, di6eeom<br>*Days*	text (integer, Min: 0, Max: 30)
	229	di7a<br><br>Show the field ONLY if:<br>[event] = '1'	7a. Have you ever used DESOMORPHINE (krokodil, electroshirka)<br><br>U, K:di7aru<br>M: di7aru, di7aom<br><br>Recode 'no' from 2 to 0	radio<br>1 Yes<br>0 No
	230	di7c<br><br>Show the field ONLY if:<br>[di7a] = '1' or [event] = '3' or [event] = '4' or [event] = '5'	7c. In the last 30 days, how many days did you use DESOMORPHINE (krokodil, electroshirka)<br><br>U: di7dru, di7cruf1<br>*Days*	text (integer, Min: 0, Max: 30)
	231	di7d<br><br>Show the field ONLY if:<br>[country] = '3' and ([event] = '3' or [event] = '4' or [event] = '5')	7d. In the last 30 days how many days did you inject DESOMORPHINE (krokodil, electroshirka)?<br>U: di7druf1<br>*Days*	text (integer, Min: 0, Max: 30)
	232	di7e<br><br>Show the field ONLY if:<br>[di7a] = '1' and [screen_arm_1][country] <> '3' and [event] = '1'	7e. In the last 30 days BEFORE THIS INCARCERATION, how many days did you inject DESOMORPHINE (krokodil, electroshirka)?<br>U: N/A<br>K: di7eru<br>M: di7eru, di7eom<br>*Days*	text (integer, Min: 0, Max: 30)
	233	di8c<br><br>Show the field ONLY if:<br>[country] = '3' and ([event] = '3' or [event] = '4' or [event] = '5')	8c. In the last 30 days how many days did you use Benzodiazapine or TRANQUILIZERS (seduxen, sibazone, valium, xanax, sonapaks, phenazepam, nitrazepam, clonazepam, diazepam, relanium)??<br>U: di8cruf1<br>*Days*	text (integer, Min: 0, Max: 30)
	234	di8d<br><br>Show the field ONLY if:<br>[country] = '3' and ([event] = '3' or [event] = '4' or [event] = '5')	8d. In the last 30 days how many days did you inject Benzodiazapine or TRANQUILIZERS (seduxen, sibazone, valium, xanax, sonapaks, phenazepam, nitrazepam, clonazepam, diazepam, relanium)??<br>U: di8druf1<br>*Days*	text (integer, Min: 0, Max: 30)
	235	di9a<br><br>Show the field ONLY if:<br>[event] = '1'	9a. Have you ever used STIMULANTS ('boltushka', 'vint', amphetamines, methamphetamines, 'efedra', 'jeff')<br><br>U, K:di9aru<br>M: di9aru, di9aom<br><br>recode 'no' from 2 to 0	radio<br>1 Yes<br>0 No
	236	di9c<br><br>Show the field ONLY if:<br>[country] = '3' and ([event] = '3' or [event] = '4' or [event] = '5')	9c. In the last 30 days how many days did you use STIMULANTS ('boltushka', 'vint', amphetamines, methamphetamines, 'efedra', 'jeff')<br><br>U: di9cruf1<br>*Days*	text (integer, Min: 0, Max: 30)

	237	di9d<br><br>Show the field ONLY if:<br>[di9a] = '1'	9d. In the last 30 days, how many days did you inject STIMULANTS ('boltushka', 'vint', amphetamines, methamphetamines, 'efedra', 'jeff'))?<br><br>u: di9dru, di9druf1<br>M: di9cruf1, di9comf1<br>*Days*	text (integer, Max: 30)
	238	di9e<br><br>Show the field ONLY if:<br>[di9a] = '1' and [screen_arm_1][country] <> '3' and [event] = '1'	9e. In the last 30 days BEFORE THIS INCARCERATION, how many days did you use STIMULANTS (' boltushka', 'vint', amphetamines, methamphetamines, 'efedra', 'jeff')?<br>U: N/A<br>K: di9eru<br>M: di9eru, di9eom<br>*Days*	text (integer, Max: 30)
	239	di15a<br><br>Show the field ONLY if:<br>[event] = '1'	15a. Have you ever used more than one substance in same day (INCLUDING alcohol)<br><br>U:di15aru<br>M: di15aru, di15aom<br><br>recode 'no' from 2 to 0	radio<br>1 Yes<br>0 No
	240	di15b<br><br>Show the field ONLY if:<br>[di15a] = '1'	15c. In the last 30 days, how many days did you use more than one substance in same day (INCLUDING alcohol)<br>U: di15bruf1<br>M: di15bruf1, di15bomf1<br><br>*Days*	text (integer, Min: 0, Max: 30)
	241	di15c<br><br>Show the field ONLY if:<br>[di15a] = '1'	15c. In the last 30 days, how many days did you inject more than one substance in same day (INCLUDING alcohol)<br><br>U: di15cru, di15cruf1<br><br>*Days*	text (integer, Min: 0, Max: 30)
	242	di15e<br><br>Show the field ONLY if:<br>[di15a] = '1' and [screen_arm_1][country] <> '3' and [event] = '1'	15e. In the last 30 days BEFORE THIS INCARCERATION how many days did you use more than one substance in same day (INCLUDING alcohol)<br>U: N/A<br>K: di15eru<br>M: di15eru, di15eom<br><br>*Days*	text (integer, Min: 0, Max: 30)
	243	di15f<br><br>Show the field ONLY if:<br>[di15a] = '1' and [screen_arm_1][country] <> '3' and [event] = '1'	15f. In the last 30 days BEFORE THIS INCARCERATION, how many days did you inject more than one substance in same day (INCLUDING alcohol)?<br>U: N/A<br>K: di15fru<br>M: di15fru, di15fom<br>*Days*	text (integer, Min: 0, Max: 30)
	244	di18	18. At this moment, how much do you crave opioids (including heroin and pain killers) on a scale of 0 to 10? (Select a point on the line with 0 being no craving whatsoever and 10 being I think about it all the time)<br><br>U,K:di18ru, di18ruf1<br>M: di18ru, di18om, di18ruf1, di18omf1<br>*0 - No craving whatsoever; 10 - I think about it all the time*	radio<br>0 0<br>1 1<br>2 2<br>3 3<br>4 4<br>5 5<br>6 6<br>7 7<br>8 8<br>9 9<br>10 10<br><br>Custom alignment: LH

	245	di19	19. In the last 30 days, how many days did you inject any kind of drug?<br><br>U,K:di19ru, di19ruf1<br>M: di19ru, di19om, di19ruf1, di19omf1<br>*Days*	text (integer, Min: 0, Max: 30)
	246	di20<br><br>Show the field ONLY if:<br>[di19] > 0	20. On days that you injected, on average, how many times did you inject per day?<br><br>U, K:di20ru, di20ruf1<br>M: M: di20ru, di20om, di20ruf1, di20omf1<br>*Times per day*	text (integer, Min: 0, Max: 30)
	247	inject_freq	Injection frequency	calc<br>Calculation: [di19]*[di20]<br>Custom alignment: RH
	248	di21	21. Based on your response, you injected [inject_freq] times. Of these [inject_freq] times you injected, Please, move the adjustable bar to estimate approximately about how many times you shared (gave/received, lent, bought/sold) a needle or a syringe to another person after you USED it to inject in the past 30 days.<br><br>U, K:di21ru, di21ruf1<br>M: di21ru, di21om, di21ruf1, di21omf1	slider<br>Slider labels: 0% None of the time, 50% About half of the time, 100% All of the time<br>Custom alignment: RV
	249	di22	22. Of these [inject_freq] times, please, move the adjustable bar to estimate approximately about how many times did you "share" common equipment for preparing your drug to inject in the last 30 days.<br><br>U,K:di22ru, di22ruf1<br>M: di22ru, di22om , di22ruf1, di22omf1	slider<br>Slider labels: 0% None of the time, 50% About half of the time, 100% All of the time<br>Custom alignment: RV
	250	di23	23.Please, move the adjustable bar to estimate approximately about how many times did you receive/ buy an already filled syringe that you later used to inject (i.e. you did not see how it was filled)?<br><br>U:di23ruf1	slider<br>Slider labels: 0% None of the time, 50% About half of the time, 100% All of the time<br>Custom alignment: RV
	251	di24	24. Please, move the adjustable bar to estimate approximately about how many times did you use a syringe, filled by someone from his / her already used syringe (direct or reverse filling, several doses in one syringe)<br><br>U:di24ruf1	slider<br>Slider labels: 0% None of the time, 50% About half of the time, 100% All of the time<br>Custom alignment: RV
	252	inventory_of_drug_use_complete	Section Header: *Form Status*<br>Complete?	dropdown<br>0 Incomplete<br>1 Unverified<br>2 Complete
Instrument: **Overdose** (overdose)				Collapse
	253	od_intro	The next few questions will be about the drug overdose. By overdose we mean occurrence of such symptoms as very slow or shallow breathing or not breathing at all, very slow, faint pulse or no pulse at all, falling unconscious, black out, fainting, unconscious condition, blue lips, fingernails or toenails, pinpoint pupils, eyes rolled back, no response to shaking, calling your name, pain (deep sleep). These effects occur from using too much opioids. We are not asking about effects from using too much of other drugs like stimulants or alcohol (also you might also be using other drugs when it happens). We also are not asking about a "heavy nod", which is not an overdose. Based on this understanding:	descriptive

	254	od2<br><br>Show the field ONLY if:<br>[event] = '1'	OD2. How many times have you experienced drug overdose during the last 6 months?<br>U, K: od2ru<br>*times*	text (integer, Min: 0, Max: 20)
	255	od2_followup<br><br>Show the field ONLY if:<br>[event] = '3' or [event] = '4' or [event] = '5'	2. How many times have you experienced drug overdose during the last 30 days?<br><br>U: od2ruf<br>M: od2ru1, od2om1	text (integer, Min: 0, Max: 10)
	256	od3<br><br>Show the field ONLY if:<br>[event] = '1'	OD3. How many times did you witness someone experience a drug overdose during the last 6 months?<br><br>U, K:od3ru<br>*times*	text (integer, Min: 0, Max: 20)
	257	od3_followup<br><br>Show the field ONLY if:<br>[event] = '3' or [event] = '4' or [event] = '5'	3. How many times did you witness someone experience a drug overdose during the last 30 days?<br><br>U: od3ruf<br>M: od3ru1, od3om1	text (integer, Min: 0, Max: 20)
	258	od4<br><br>Show the field ONLY if:<br>[screen_arm_1][country] <> '3'	4. Specify how you got the drugs that lead to overdose during the last 6 months? Did you receive the drugs from within prison or in another way? U: N/A K: od4_kyrru, 1 and 3 only (except there was somebody with 2 selected???)	radio<br>1 From within the prison<br>2 Inside the colony, but from another source (not from the obshchak)<br>3 From elsewhere
	259	od4a<br><br>Show the field ONLY if:<br>[od4] = '3'	4a. Specify where U: N/A, K: od4aru	text
	260	od5a<br><br>Show the field ONLY if:<br>[screen_arm_1][country] <> '3'	5. How safe is it to use drugs received from within prison U: N/A, K:od5aru	radio<br>1 1 Very Unsafe<br>2 2<br>3 3<br>4 4<br>5 5 Completely Safe
	261	od5_kyr<br><br>Show the field ONLY if:<br>[screen_arm_1][country] = '2'	5. How safe is it to use drugs received from Obshchak U: N/A, K:od5akyrru	radio<br>1 1 Very Unsafe<br>2 2<br>3 3<br>4 4<br>5 5 Completely Safe
	262	od5b<br><br>Show the field ONLY if:<br>[screen_arm_1][country] = '2'	5b. How dangerous is it to take drugs obtained in a different way in the colony (not from the obshchak)? U: N/A, K:od5bkyrru	radio<br>1 1 Very Unsafe<br>2 2<br>3 3<br>4 4<br>5 5 Completely Safe
	263	od5c<br><br>Show the field ONLY if:<br>[screen_arm_1][country] <> '3'	5. How safe is it to use drugs obtained in the community? U: N/A, K:od5cru	radio<br>1 1 Very Unsafe<br>2 2<br>3 3<br>4 4<br>5 5 Completely Safe

	264	overdose_complete	Section Header: *Form Status*<br>Complete?	dropdown<br>0 Incomplete<br>1 Unverified<br>2 Complete
Instrument: **Registration At State Narcology Service** (registration_at_state_narcology_service)				Collapse
	265	reg_info	The next question will be about the registration at Narcology services. By registration we mean that you have officially been diagnosed with opioid dependence and registered at narcological dispensary. We need this information only for studying barriers and facilitators to OST entry and retention. This information is strictly confidential and will be used only for the research purposes.	descriptive
	266	rg_interviewer_id<br>Show the field ONLY if:<br>[screen_arm_1][country] <> '3'	Interviewer ID: K: regintru	text
	267	rg_interview_date<br>Show the field ONLY if:<br>[screen_arm_1][country] <> '3'	Date of interview K: regdtru	text (date_mdy)
	268	rg1	Are you currently an "OFFICIALLY" registered patient at Narcology Service?<br>U, K: rg1ru	radio<br>1 Yes, I voluntarily got registered to help my addiction<br>2 Yes, but I got registered only because the police forced me to<br>3 No, I am definitely not registered<br>4 Do not remember
	269	registration_at_state_narcology_service_complete	Section Header: *Form Status*<br>Complete?	dropdown<br>0 Incomplete<br>1 Unverified<br>2 Complete
Instrument: **Current Drug Dependency Treatment** (current_drug_dependency_treatment)				Collapse
	270	dtinfo_curr	This next series of questions are going to ask about your current treatment from opioid dependence. By treatment we mean any kind of intervention (session, taking medications, and counseling) that you willingly or unwillingly is participating in. All information is strictly confidential and will be used only for scientific purposes.	descriptive
	271	dt12	DT12. Are you currently receiving any kind of treatment for your opioid dependence?<br>RECODE 2 to 0<br>U, K: dt12ru	radio<br>1 Yes<br>0 No

	272	dt13<br><br>Show the field ONLY if:<br>[dt12]='1'	13. Which treatment for opioid dependence are you currently receiving? [check all that apply]<br>U, K:dt13ru	checkbox<br>1 dt13___1 Detoxification [detox]<br>2 dt13___2 Rehabilitation (counseling)<br>3 dt13___3 Abstinence therapeutic community (for example: work camp, religious camp)<br>4 dt13___4 Psychotherapy and counseling (no medications provided)<br>5 dt13___5 Treatment in psychiatric hospital with psychotropic medications provided<br>6 dt13___6 Harm reduction program (excluding OST)<br>7 dt13___7 Other (enter) ______________________________<br>8 dt13___8 Opioid substitution treatment (with buprenorphine or methadone)(Kyrg)
	273	dt13o<br><br>Show the field ONLY if:<br>[dt13(7)] = '1'	Specify Other:<br>U, K: dt13oru	text
	274	dt14yrs<br><br>Show the field ONLY if:<br>[dt12]='1'	14. How long have you been receiving your current treatment (if you receive several treatments, please indicate time regarding the most important type of treatment for you)?<br>U, K: dt14yrsru<br>*Years*	text (integer)
	275	dt14mos<br><br>Show the field ONLY if:<br>[dt12] = '1'	DT14 Months:<br>U, K: dt14mosru<br>*Months*	text (integer)
	276	dt14days<br><br>Show the field ONLY if:<br>[dt12] = '1' and [screen_arm_1][country]='2'	DT14 Days:<br>U: N/A, K: dt14daysru<br>*Months*	text (integer)
	277	dt14a<br><br>Show the field ONLY if:<br>[dt12] = '1'	14a. Indicate the type of treatment<br>U, K: dt14aru	text
	278	current_drug_dependency_treatment_complete	Section Header: *Form Status*<br>Complete?	dropdown<br>0 Incomplete<br>1 Unverified<br>2 Complete
Instrument: **Treatment Effectiveness Assessment** (treatment_effectiveness_assessment) Collapse				
	279	tealabel	The TEA asks you to express the extent of changes for the better from your involvement in the program to this point (or how things are if it's your first TEA or baseline). For each area, think about how things have become better and circle the results on the scale below: the more you have improved, the higher the number - from 1 (not better at all) to 10 (very much better).	descriptive

	280	tea_su<br>Show the field ONLY if:<br>[current_on_oat] = 1	Substance use: How much better are you with drug and alcohol use? Consider the frequency and amount of use, money spent on drugs, amount of drug craving, time spent being loaded, being sick, in trouble and in other drug-using activities, etc. U, K: tea_suru	radio (Matrix)<br>1 — 1 None or not much<br>2 — 2<br>3 — 3<br>4 — 4<br>5 — 5 Better<br>6 — 6<br>7 — 7<br>8 — 8<br>9 — 9<br>10 — 10 Much better
	281	tea_health<br>Show the field ONLY if:<br>[current_on_oat] = 1	Health: Has your health improved? In what way and how much? Think about your physical and mental health: Are you eating and sleeping properly, exercising, taking care of health problems or dental problems, feeling better about yourself, etc? U, K: tea_healthru	radio (Matrix)<br>1 — 1 None or not much<br>2 — 2<br>3 — 3<br>4 — 4<br>5 — 5 Better<br>6 — 6<br>7 — 7<br>8 — 8<br>9 — 9<br>10 — 10 Much better
	282	tea_life<br>Show the field ONLY if:<br>[current_on_oat] = 1	Lifestyle: How much better are you in taking care of personal responsibilities? Think about your living conditions, family situation, employment, relationships: Are you paying your bills? Following through with your personal or professional commitments? U: tea_liferu	radio (Matrix)<br>1 — 1 None or not much<br>2 — 2<br>3 — 3<br>4 — 4<br>5 — 5 Better<br>6 — 6<br>7 — 7<br>8 — 8<br>9 — 9<br>10 — 10 Much better
	283	tea_com<br>Show the field ONLY if:<br>[current_on_oat] = 1	Community: Are you a better member of the community? Think about things like obeying laws and meeting your responsibilities to society: Do your actions have positive or negative impacts on other people? U, K: tea_comru	radio (Matrix)<br>1 — 1 None or not much<br>2 — 2<br>3 — 3<br>4 — 4<br>5 — 5 Better<br>6 — 6<br>7 — 7<br>8 — 8<br>9 — 9<br>10 — 10 Much better
	284	tea_notes	Comments/Notes K: N/A	notes

	285	treatment_effectiveness_assessment_complete	Section Header: *Form Status*<br>Complete?	dropdown<br>0 Incomplete<br>1 Unverified<br>2 Complete
Instrument: **Attitudes Toward And Knowledge About Ost** (attitudes_toward_and_knowledge_about_ost)				Collapse
	286	caslabel	To what extent do you agree or disagree with the following statements? Check the box that best corresponds.	descriptive
	287	ost1	1. OST services should be available in the community so that all people who suffer from opioid addiction and OST can receive it. U, K: ost1ru	radio (Matrix)<br>1 1 Strongly disagree<br>2 2<br>3 3<br>4 4<br>5 5 Strongly agree
	288	ost2	2. OST services should be introduced into prisons so that all inmates who suffer from opioid addiction and want opioid substitution therapy can receive it. U, K: ost2ru	radio (Matrix)<br>1 1 Strongly disagree<br>2 2<br>3 3<br>4 4<br>5 5 Strongly agree
	289	ost3	3. For people who have opioid addiction, it would be much better to treat them with buprenorphine rather than methadone. U, K: ost3ru	radio (Matrix)<br>1 1 Strongly disagree<br>2 2<br>3 3<br>4 4<br>5 5 Strongly agree
	290	ost4	4. OST reduces opioid dependent individuals' consumption of illicit opiates. U, K: ost4ru	radio (Matrix)<br>1 1 Strongly disagree<br>2 2<br>3 3<br>4 4<br>5 5 Strongly agree
	291	ost5	5. OST reduces opioid dependent individuals' risk of acquiring or transmitting HIV.U, K: ost5ru	radio (Matrix)<br>1 1 Strongly disagree<br>2 2<br>3 3<br>4 4<br>5 5 Strongly agree
	292	ost6	6. OST improves adherence to HIV medications in HIV-infected opioid dependent individuals. U, K: ost6tru	radio (Matrix)<br>1 1 Strongly disagree<br>2 2<br>3 3<br>4 4<br>5 5 Strongly agree

	293	ost7	7. OST increases opioid dependent patients' adherence to tuberculosis medication U, K: ost7ru	radio (Matrix)<br>1 1 Strongly disagree<br>2 2<br>3 3<br>4 4<br>5 5 Strongly agree
	294	ost8	8. OST decreases opioid dependent individuals' risk of dying from overdose. U, K: ost8ru	radio (Matrix)<br>1 1 Strongly disagree<br>2 2<br>3 3<br>4 4<br>5 5 Strongly agree
	295	ost9	9. OST reduces an addict's criminal activities. U, K: ost9ru	radio (Matrix)<br>1 1 Strongly disagree<br>2 2<br>3 3<br>4 4<br>5 5 Strongly agree
	296	attitudes_toward_and_knowledge_about_ost_complete	Section Header: *Form Status*<br>Complete?	dropdown<br>0 Incomplete<br>1 Unverified<br>2 Complete

Instrument: **Perceived Treatment Effectiveness** (perceived_treatment_effectiveness) Collapse

	297	e01	1. Religion U: e01_ru K: e1_ru	radio (Matrix)<br>1 1 not at all effective<br>2 2<br>3 3<br>4 4<br>5 5 very effective
	298	e02	2. Incarceration U: e02_ru, K: e2_ru	radio (Matrix)<br>1 1 not at all effective<br>2 2<br>3 3<br>4 4<br>5 5 very effective
	299	e03	3. Opioid Substitution therapy U: e03_ru, K: e3_ru	radio (Matrix)<br>1 1 not at all effective<br>2 2<br>3 3<br>4 4<br>5 5 very effective
	300	e04	4. Folk medicine U: e04_ru, K: e4_ru	radio (Matrix)<br>1 1 not at all effective<br>2 2<br>3 3<br>4 4<br>5 5 very effective

	301	e05	5. Employment U: e05_ru, K: e5_ru	radio (Matrix)<br>1 1 not at all effective<br>2 2<br>3 3<br>4 4<br>5 5 very effective
	302	e06	6. Family support U: e06_ru, , K: e6_ru	radio (Matrix)<br>1 1 not at all effective<br>2 2<br>3 3<br>4 4<br>5 5 very effective
	303	e07	7. New environment U: e07_ru, K: e7_ru	radio (Matrix)<br>1 1 not at all effective<br>2 2<br>3 3<br>4 4<br>5 5 very effective
	304	e08	8. Treatment with medication U: e08_ru	radio (Matrix)<br>1 1 not at all effective<br>2 2<br>3 3<br>4 4<br>5 5 very effective
	305	e09	9. Detox U: e09_ru, K: e9_ru	radio (Matrix)<br>1 1 not at all effective<br>2 2<br>3 3<br>4 4<br>5 5 very effective
	306	e10	10. Rehabilitation U, K: e10_ru	radio (Matrix)<br>1 1 not at all effective<br>2 2<br>3 3<br>4 4<br>5 5 very effective
	307	e11	11. Atlantis U: N/A, , K: e11_ru	radio (Matrix)<br>1 1 not at all effective<br>2 2<br>3 3<br>4 4<br>5 5 very effective
	308	e12	12. Clean Zone U: N/A, K: , K: e12_ru	radio (Matrix)<br>1 1 not at all effective<br>2 2<br>3 3<br>4 4<br>5 5 very effective

	309	e13	13. Other(specify below) U, K: e13_ru	radio (Matrix)<br>1 | 1 not at all effective<br>2 | 2<br>3 | 3<br>4 | 4<br>5 | 5 very effective
	310	e13oth	Specify Other U, K: , K: e13othru	text
	311	perceived_treatment_effectiveness_complete	Section Header: *Form Status*<br>Complete?	dropdown<br>0 | Incomplete<br>1 | Unverified<br>2 | Complete
Instrument: **Ost** (ost)				Collapse
	312	ost_label	Section Header: *OST:Entry Attempts*<br>The next series of questions are going to ask you about your interest and experience with OST (methadone/ Buprenorphine treatment). By OST program, I mean receiving methadone/ Buprenorphine in a medical facility on a regular basis and taking it or	descriptive
	313	ma1	1. Have you ever tried to enter the OST program? U, K:ma1ru	yesno<br>1 | Yes<br>0 | No
	314	ma3	How many attempts have you made at entering an OST program?	text
	315	ma4<br>Show the field ONLY if:<br>[ma1] = '1'	4. In what month was your last attempt? U, K:ma4ru	text (number, Min: 1, Max: 12)
	316	ma4yr<br>Show the field ONLY if:<br>[ma1] = '1'	4. In what year was your last attempt?<br>U, K:ma4yr_ru	text (number, Min: 1970, Max: 2018)
	317	ma5<br>Show the field ONLY if:<br>[ma1] = '1'	5. If you have not entered the program during the most recent attempt, what were the MAIN reasons for not entering the program during your most recent attempt? (check 3) U, K:ma5ru	checkbox<br>1 | ma5___1 | Does not apply (have entered the program)<br>4 | ma5___4 | Didn't want to get registered<br>5 | ma5___5 | I did not like the rules of being on the program<br>6 | ma5___6 | The hours were not convenient<br>7 | ma5___7 | The location was not convenient<br>8 | ma5___8 | I was treated poorly by the staff<br>9 | ma5___9 | I was told I was not eligible to enter the program<br>10 | ma5___10 | My family did not want me to enter the program<br>11 | ma5___11 | There was no availability for registration<br>12 | ma5___12 | don't want to be blamed for cooperation with administration (Kyr Only)<br>13 | ma5___13 | Don't want to lose my status among peers(kyr Only)
	318	ma6<br>Show the field ONLY if:<br>[ma1] = '1'	6. How many times overall did you officially enter onto OST substitution program? U:ma6ru<br>*times*	text (number, Min: 0, Max: 10)

	319	ma1a<br><br>Show the field ONLY if:<br>[ma1] = '0'	Section Header: *OST: Attitudes(Never OST)*<br><br>1a. You said you have never been in a OST program, are you interested in getting OST? U, K:ma1aru. CHANGE NO from 2 to 0	radio<br>1 Yes<br>0 No
	320	ma1a1<br><br>Show the field ONLY if:<br>[ma1a] ='0'	We would like to know why you are NOT interested in getting methadone treatment. Please, read the different statements and indicate if any of these statements describe a reason why you do not want to start treatment. You may have more than one reason. U, K:ma1a1ru	checkbox<br>1 ma1a1___1 Didn't know it existed<br>2 ma1a1___2 I do not like the rules of being on the program (hours/location)<br>3 ma1a1___3 I was treated poorly by the staff<br>4 ma1a1___4 I was told I was not eligible to enter the program<br>5 ma1a1___5 Didn't want to get registered (medical record)<br>6 ma1a1___6 Methadone will destroy my health<br>7 ma1a1___7 I will have to take Methadone all my life<br>8 ma1a1___8 My family and friends have a negative feeling about OST<br>9 ma1a1___9 My friends had bad experience of OST (their health got worse)<br>10 ma1a1___10 Methadone is free and thus bad quality<br>11 ma1a1___11 I don't want my friends know I am in OST<br>12 ma1a1___12 I think Methadone is just another drug<br>15 ma1a1___15 Don't want to be blamed for cooperation with administration<br>16 ma1a1___16 Don't want to lose my status among peers
	321	ma1b<br><br>Show the field ONLY if:<br>[ma1a] ='1'	You said you were interested in treatment with methadone. How difficult will it be for you to initiate such treatment on a scale from 0 to 10 (0 = not difficult at all; 10 = very difficult)? U, K:ma1bru	radio<br>0 0 = Not difficult at all<br>1 1<br>2 2<br>3 3<br>4 4<br>5 5<br>6 6<br>7 7<br>8 8<br>9 9<br>10 10 = Very difficult
	322	melabel<br><br>Show the field ONLY if:<br>[ma6]>0	Section Header: *OST: Program Experience(Previously on OST)*<br><br>The next series of questions are going to ask you about your experiences with methadone or buprenorphine. By methadone/buprenorphine treatment program, we mean receiving methadone/buprenorphine in sites of OST on a regular basis and taking it orally to treat addiction. You may have different experience in treatment, both negative and positive. We value your honest answers. @HIDDEN	descriptive

	323	me1<br><br>Show the field ONLY if:<br>[ma6]>0	1. What were your reasons to begin methadone program [check all that apply] U, K:me1ru me1ruf1	checkbox<br>1	me1___1	I wanted to improve my health<br>2	me1___2	I wanted to make a change in the circles I was moving in<br>3	me1___3	I wanted to reduce my drug use because I was using too much<br>4	me1___4	I wanted to end my use of illegal opioids permanently<br>5	me1___5	I needed a break from my habit because my life was too chaotic<br>6	me1___6	Financing drug consumption was too expensive<br>7	me1___7	I wanted to stop committing crimes for my habit<br>8	me1___8	I wanted to take better care of my family<br>9	me1___9	I was afraid of losing my job<br>10	me1___10	I wanted to (be able to) work again<br>11	me1___11	I wanted to get clean so I could get my HIV treated<br>12	me1___12	I was concerned of being arrested again / imprisoned<br>13	me1___13	I was worried about getting an infection or contracting a disease<br>14	me1___14	I was afraid I might overdose<br>15	me1___15	Pregnancy<br>16	me1___16	My family wanted me to start methadone treatment<br>17	me1___17	Someone else wanted me to start methadone treatment (please specify who _________)<br>18	me1___18	Other (please specify) ___________________________<br><br>Custom alignment: LV
	324	me1q<br><br>Show the field ONLY if:<br>[me1(17)] = '1'	Someone else wanted me to start methadone treatment (please specify who) U, K:me1qru me1qruf1	text
	325	me1r<br><br>Show the field ONLY if:<br>[me1(18)] = '1'	Other (please specify) __ U:merru K: me1rru me1rruf1	text

	326	me2<br><br>Show the field ONLY if:<br>[ma6]>0	2. Where did you obtain information about OST program prior to your start of the program? (check all that apply. U, K:me2ru me2ruf1	checkbox<br>1 | me2___1 | A. Probation staff<br>2 | me2___2 | B. Probation doctor<br>3 | me2___3 | C. Doctors<br>4 | me2___4 | D. Friends<br>5 | me2___5 | E. Other OST patients<br>6 | me2___6 | F. People who use drugs<br>7 | me2___7 | G. Prisoners<br>8 | me2___8 | H. Psychologists<br>9 | me2___9 | I. Family members<br>10 | me2___10 | J. NGO staff<br>11 | me2___11 | K. Other (specify): ____________________<br><br>Custom alignment: LV
	327	me2_partner<br><br>Show the field ONLY if:<br>[ma6] > 0 and [index_partner] ='2'	ME2. Where did you obtain information about OST program prior to your start of the program? (check all that apply)	checkbox<br>3 | me2_partner___3 | A. Doctors<br>4 | me2_partner___4 | B. Friends<br>5 | me2_partner___5 | C. Other OST patients<br>6 | me2_partner___6 | D. People who use drugs<br>7 | me2_partner___7 | E. Prisoners<br>8 | me2_partner___8 | F. Psychologists<br>9 | me2_partner___9 | G. Family members<br>10 | me2_partner___10 | H. NGO staff<br>11 | me2_partner___11 | I. Other (specify): ____________________<br><br>Custom alignment: LV
	328	me2oth<br><br>Show the field ONLY if:<br>[me2(11)] = '1'	Other (specify): U, k:me2othru me2othruf1	text
	329	me2a<br><br>Show the field ONLY if:<br>[ma6]>0	2a. Which of the below mentioned medicines you take, being in the OST program? U: me2aru me2aruf1 K: N/A	checkbox<br>1 | me2a___1 | Methadone<br>2 | me2a___2 | Buprenorphine<br><br>Custom alignment: LV
	330	me3<br><br>Show the field ONLY if:<br>[ma6]>0	ME3. Were you ever told at any time that in order to stay on the OST program that you would have to pay someone money to keep receiving your OST? U, K:me3ru me3ruf1	radio<br>1 | Yes<br>0 | No
	331	me4<br><br>Show the field ONLY if:<br>[ma6]>0	4. Were you ever told at any time that in order to change your methadone/buprenorphine dose that you would have to pay someone money? U, K:me4ru me4ruf1	radio<br>1 | Yes<br>0 | No
	332	me4a<br><br>Show the field ONLY if:<br>[ma6]>0	ME4a. Have you been on OST today or in the last 10 days? U, K:me4aru me4aruf1	radio<br>1 | Yes<br>0 | No
	333	me5<br><br>Show the field ONLY if:<br>[ma6]>0 and [screen_arm_1][country] = '3' and [event] = '1'	5. On your way to or from the methadone program, were you EVER stopped by police and questioned? U:me5ru, K: N/A	radio<br>1 | Yes<br>0 | No

	334	me6<br><br>Show the field ONLY if:<br>[me5] = '1'	6. When you were stopped by the police on your way to or from the methadone program , were you ever asked to pay them money in order not to be arrested? U:me6ru, K: N/A	radio, Required<br>1 Yes<br>0 No
	335	me7<br><br>Show the field ONLY if:<br>[me5]='1'	7. When you were stopped by the police on your way to or from the methadone program, were you ever asked to cooperate with them (e.g. sell somebody out, have sex with them) in order not to be arrested? U:me7ru, K: N/A	radio, Required<br>1 Yes<br>0 No
	336	me8<br><br>Show the field ONLY if:<br>[me5]='1'	8. When you were stopped by the police on your way to or from the methadone program, were you ever arrested and detained? U:me8ru, K: N/A	radio, Required<br>1 Yes<br>0 No
	337	me9<br><br>Show the field ONLY if:<br>[me8]='1'	9. If YES (you were arrested and detained) did you experience abstinence syndrome (withdrawal)? U:me9ru, K: N/A	radio, Required<br>1 Yes<br>0 No
	338	me10<br><br>Show the field ONLY if:<br>[ma6] > 0 and [event] = '1'	10. Were you in a methadone treatment program in the last year? U:me10ru, K: N/A	radio, Required<br>1 Yes<br>0 No<br><br>Custom alignment: LV
	339	me11m<br><br>Show the field ONLY if:<br>[me4a] = '1' or [ma6] > 0	11m. When did you enter the OST Program? (Month) U:me11mruc me11mrucf1, K: me11mru	text (number, Min: 1, Max: 12)
	340	me11y<br><br>Show the field ONLY if:<br>[me4a] = '1' or [ma6] > 0	11y. When did you enter the OST Program? (Year) U: me11yruc me11yrucf1<br>*YYYY*	text (number, Min: 1980, Max: 2019)
	341	me12y<br><br>Show the field ONLY if:<br>[screen_arm_1][country] = '3' and [event] = '1' and ([me4a] = '1' or [ma6] > 0)	12y. What was the total length of time on the most recent methadone treatment program including short breaks? U;me12yruc K: N/A<br>*Years*	text (number, Min: 0, Max: 20)
	342	me12m<br><br>Show the field ONLY if:<br>[screen_arm_1][country] = '3' and [event] = '1' and ([me4a] = '1' or [ma6] > 0)	12m. What was the total length of time on the most recent methadone treatment program including short breaks? U:me12mruc<br>*Months*	text (number, Min: 0, Max: 11)
	343	me12md<br><br>Show the field ONLY if:<br>[screen_arm_1][country] = '3' and [event] = '1' and ([me4a] = '1' or [ma6] > 0)	12d. What was the total length of time on the most recent methadone treatment program including short breaks? (Days) U: me12mdruc, K: N/A<br>*Days*	text (number, Min: 0, Max: 30)
	344	me13<br><br>Show the field ONLY if:<br>[screen_arm_1][country] = '3' and [event] = '1' and ([me4a] = '1' or [ma6] > 0)	13. Where were you last on OST? U: me13ruc, K: N/A	radio<br>1 In prison<br>2 In SIZO<br>3 In the community
	345	me14<br><br>Show the field ONLY if:<br>[me4a] = '1' or [ma6] > 0	14. Are you satisfied with the length of your OST? U:me14ruc me14rucf1, K: me14ru	radio<br>1 Yes<br>0 No
	346	me15<br><br>Show the field ONLY if:<br>[me14] = '0'	15. If No, do you wish you had been treated for a shorter or longer period? U:me15ruc me15rucf1, K: me15ru	radio<br>1 Shorter<br>2 Longer
	347	me16<br><br>Show the field ONLY if:<br>[ma6] > 0	16. What was your dosage the day before the discharge U:me16ruc, K: What is your current dosage? U:Me16ru me16rucf1<br>*mg*	text (number, Min: 0)

	348	me16a<br><br>Show the field ONLY if:<br>[ma6] > 0	16a. I think my dose of methadone/buprenorphine should be.. U: N/A in baseline me16arucf1, K: me16aru	radio<br>1 Higher<br>2 Lower<br>3 Stay the same
	349	me17<br><br>Show the field ONLY if:<br>[ma6] > 0	17. On the scale from 1 to 5 how hard was it for you to INCREASE your prescribed dose of methadone/buprenorphine (1 - extremely easy, 5 - extremely hard)? U: me17ruc me17rucf1, K: me17ru	radio<br>1 1 Extremely easy<br>2 2 Somewhat easy<br>3 3 Neither hard nor easy<br>4 4 Somewhat hard<br>5 5 Extremely hard<br>6 I never asked to INCREASE methadone
	350	me18<br><br>Show the field ONLY if:<br>[ma6] > 0	18. On the scale from 1 to 5 how hard was it for you to DECREASE your prescribed dose of methadone/buprenorphine (1 - extremely easy, 5 - extremely hard)? U: me18ruc me18rucf1, K: me18ru	radio<br>1 1 Extremely easy<br>2 2 Somewhat easy<br>3 3 Neither hard nor easy<br>4 4 Somewhat hard<br>5 5 Extremely hard<br>6 I never asked to DECREASE methadone
	351	me19<br><br>Show the field ONLY if:<br>[ma6] > 0	19. Did you experience any side effects from your prescribed dose of methadone that somehow impacted your interest in continuing? U: me19ruc me19rucf1, K: me19ru	radio<br>1 Yes<br>0 No
	352	me20<br><br>Show the field ONLY if:<br>[ma6] > 0	20. Did you ever keep a part of your prescribed dose of methadone for yourself to take later? U, K: me20ruc me20rucf1	radio<br>1 Yes<br>0 No
	353	me21<br><br>Show the field ONLY if:<br>[me20]='1'	21. If Yes, for what reasons? [check all that apply] U: me21ruc me21rucf1, K: N/A	checkbox<br>1 me21___1 To save for myself for later that day<br>2 me21___2 For travelling purposes<br>3 me21___3 For a weekend<br>4 me21___4 Other (please specify)________________________
	354	me21oth<br><br>Show the field ONLY if:<br>[me21(4)] = '1'	Other(please specify) U: me21othruc me21othrucf1, K: N/A	text
	355	me22<br><br>Show the field ONLY if:<br>[ma6] > 0	22. Did you ever have short (e.g., less than 10 days) interruptions in your methadone treatment? U: me22ruc me22rucf1, K: me22ru	radio<br>1 Yes<br>0 No
	356	me23<br><br>Show the field ONLY if:<br>[me22] = '1'	23. For what reasons did you have interruptions in treatment? U: me23ruc me23rucf1, K: me23ru	notes<br>Custom alignment: RH
	357	me24<br><br>Show the field ONLY if:<br>[ma6] > 0	24. Did you tell anyone that you were on methadone program? U: me24ruc me24rucf1, K: me24ru	radio<br>1 Yes<br>0 No
	358	me24a<br><br>Show the field ONLY if:<br>[me4a]= '1'	How often do you take illegal drugs in addition to or instead of your methadone/buprenorphine medication? U: N/A in baseline me24arucf1 K: me24aru	radio<br>1 Daily<br>2 3-4 times per week<br>3 Once per week<br>4 1-2 per month<br>5 Never

	359	me25<br>Show the field ONLY if:<br>[ma6] > 0 and [event] = '1'	25. What was the primary reason for your leaving the program? [check all that apply]: U: me25ruc , K: N/A	checkbox<br>1 me25___1 Personal decision to complete MMT<br>2 me25___2 Expelled from the program due to misconduct/truancy<br>3 me25___3 I thought that recovered<br>4 me25___4 Putting in an insulator<br>5 me25___5 Moved to live elsewhere where there is no MMT<br>6 me25___6 I do not like the rules for program participants (place, time)<br>7 me25___7 Workers program badly I was treated<br>8 me25___8 I was told that I was not fit for the program
	360	me26<br>Show the field ONLY if:<br>[ma6] > 0	26. Do you use any of these substances concurrently with/instead of methadone/buprenorphine? If yes, which ones? U, K: me26ruc me26rucf1	checkbox<br>1 me26___1 Klonasepam<br>2 me26___2 Diasepam<br>3 me26___3 Fenasepam<br>4 me26___4 Somnol<br>5 me26___5 Dimedrol<br>6 me26___6 ALCOHOL<br>7 me26___7 None of those<br>8 me26___8 OTHER:
	361	me26oth<br>Show the field ONLY if:<br>[me26(8)] = '1'	Other: U: me26othruac, K: me26othrua me26othruacf1	text
	362	me27<br>Show the field ONLY if:<br>[ma6] > 0	27. Why did you use these concurrently with methadone/buprenorphine? U: me27ruc me27rucf1, K: me27ru	checkbox<br>1 me27___1 I need it when I miss visiting the site<br>2 me27___2 I need to do it when I go away to a place where there is no OST<br>3 me27___3 Methadone/buprenorphine does not take away my cravings<br>4 me27___4 Sometimes I want to feel high<br>5 me27___5 Other, specify ____________________
	363	me27oth<br>Show the field ONLY if:<br>[me27(5)] = '1'	Other (please specify) U: me27othruc me27othrucf1	text
	364	me27a<br>Show the field ONLY if:<br>[ma6] > 0 and [screen_arm_1][country]='2'	27a. How long do you want to continue taking therapy? U: me27arucf1 K:me27aru	checkbox<br>1 me27a___1 I don't want to be on the program any longer<br>2 me27a___2 Several Days<br>3 me27a___3 Several Months<br>4 me27a___4 Several Years<br>5 me27a___5 Forever<br>6 me27a___6 Until I have completed my term on probation
	365	me27b	27b. How long does it take to get to the OST site? U: me27brucf1	text
	366	me27c	27c. How do you get there (public transportation, private car?) U: me27crucf1	text

	367	me28<br><br>Show the field ONLY if:<br>[ma6] > 0	Section Header: *The next few questions will be about how methadone treatment has impacted your everyday life. Please indicate your answer on the scale from 1 to 5, where 1 - very well; 5 - not at all)*<br><br>28.How did methadone/buprenorphine work for you to reduce your opiate use when you were in a OST treatment program? U: me28ruc me28rucf1, K: me28ru	radio (Matrix)<br>1 Very well<br>2 Moderately well<br>3 Neither helped nor harmed<br>4 Not very well<br>5 Not at all
	368	me29<br><br>Show the field ONLY if:<br>[ma6] > 0	29. How well did methadone/buprenorphine treatment improve the quality of your life? U: me29ruc me29rucf1, K: me29ru	radio (Matrix)<br>1 Very well<br>2 Moderately well<br>3 Neither helped nor harmed<br>4 Not very well<br>5 Not at all
	369	me30<br><br>Show the field ONLY if:<br>[ma6] > 0	30. How well did methadone/buprenorphine treatment improve the quality of your relationships with your family and friends? U: me30ruc me30rucf1, K: me30ru	radio (Matrix)<br>1 Very well<br>2 Moderately well<br>3 Neither helped nor harmed<br>4 Not very well<br>5 Not at all
	370	me31<br><br>Show the field ONLY if:<br>[ma6] > 0	31. If you knew someone with an opioid abuse problem, would you refer him or her to methadone treatment? U: me31ruc me31rucf1, K: me31ru	radio<br>1 Yes<br>0 No
	371	me32<br><br>Show the field ONLY if:<br>[ma6] > 0	32. Overall, how satisfied were you with your OST therapy to date?U: me32ruc me32rucf1, K;me32ru	radio<br>1 Not satisfied at all<br>2 Not very satisfied<br>3 Neutral<br>4 Somewhat satisfied<br>5 Very satisfied
	372	me33<br><br>Show the field ONLY if:<br>[me4a] = '0'	33. Will you try to restart methadone treatment sometime?U, K: me33ru	radio<br>1 Yes<br>0 No
	373	me33a<br><br>Show the field ONLY if:<br>[me33] = '0'	33? We would like to know why you are not interested in restarting methadone treatment.<br>I DO NOT WANT TO RESTART METHADONE TREATMENT BECAUSE: U, K: me33aru	notes
	374	me33b<br><br>Show the field ONLY if:<br>[me33] = '1'	33b You said you will try to restart methadone treatment in future, do you anticipate it will be difficult to restart methadone treatment? How difficult will it be to initiate such treatment on a scale from 0 to 10<br>(0 = not difficult at all; 10 = very difficult)? ,U, K: me33bru	radio<br>0 0-not difficult at all<br>1 1<br>2 2<br>3 3<br>4 4<br>5 5<br>6 6<br>7 7<br>8 8<br>9 9<br>10 10 - very difficult

	375	ost_complete	Section Header: *Form Status*<br>Complete?	dropdown<br>0 Incomplete<br>1 Unverified<br>2 Complete
Instrument: **Health Related Quality Of Life** (health_related_quality_of_life) Collapse				
	376	qol1	1. Would you say that in general your health is?	radio<br>1 Excellent<br>2 Very good<br>3 Good<br>4 Fair<br>5 Poor
	377	qol2	2. Now thinking about your physical health, which includes physical illness and injury, for how many days during the past 30 days was your physical health not good?<br>*Number of days*	text (integer, Min: 0, Max: 30)
	378	qol3	3. Now thinking about your mental health, which includes stress, depression, and problems with emotions, for how many days during the past 30 days was your mental health not good?<br>*Number of days*	text (integer, Min: 0, Max: 30)
	379	qol4	4. During the past 30 days, for about how many days did poor physical or mental health keep you from doing your usual activities, such as self-care, work, or recreation?<br>*Number of days*	text (integer, Min: 0, Max: 30)
	380	hqollabel	These next questions are about physical, mental, or emotional problems or limitations you may have in your daily life.	descriptive
	381	qol5	5. Are you LIMITED in any way in any activities because of any impairment or health problem?	radio<br>1 Yes<br>0 No<br>8 Don't know/Unsure
	382	qol6	6. What is the MAJOR impairment or health problem that limits your activities?	radio<br>1 Arthritis/rheumatism<br>2 Back or neck problem<br>3 Fractures, bone/joint injury<br>4 Walking problem<br>5 Lung/breathing problem<br>6 Hearing problem<br>7 Eye/vision problem<br>8 Heart problem<br>9 Stroke problem<br>10 Hypertension/high blood pressure<br>11 Diabetes<br>12 Cancer<br>13 Depression/anxiety/emotional problem<br>14 Other impairment/problem
	383	qol6oth<br>Show the field ONLY if:<br>[qol6] = '14'	Other impairment/ problem	text
	384	qol7d	7. For HOW LONG have your activities been limited because of your major impairment or health problem?<br>Days	text (integer, Min: 0, Max: 30)
	385	qol7m	7. Months	text (integer, Min: 0, Max: 12)

	386	qol7y	Years	text (integer, Min: 0, Max: 70)
	387	qol8	8. Because of any impairment or health problem, do you need the help of other persons with your PERSONAL CARE needs, such as eating, bathing, dressing, or getting around the house?	yesno<br>1 Yes<br>0 No
	388	qol9	9. Because of any impairment or health problem, do you need the help of other persons in handling your ROUTINE needs, such as everyday household chores, doing necessary business, shopping, or getting around for other purposes?	yesno<br>1 Yes<br>0 No
	389	qol10	10. During the past 30 days, for about how many days did PAIN make it hard for you to do your usual activities, such as self-care, work, or recreation?<br>*Number of days*	text (integer, Min: 0, Max: 30)
	390	qol11	11. During the past 30 days, for about how many days have you felt SAD, BLUE, or DEPRESSED?<br>*Number of days*	text (integer, Min: 0, Max: 30)
	391	qol12	12. During the past 30 days, for about how many days have you felt WORRIED, TENSE, or ANXIOUS?<br>*Number of days*	text (integer, Min: 0, Max: 30)
	392	qol13	13. During the past 30 days, for about how many days have you felt you did NOT get ENOUGH REST or SLEEP?<br>*Number of days*	text (integer, Min: 0, Max: 30)
	393	qol14	14. During the past 30 days, for about how many days have you felt VERY HEALTHY AND FULL OF ENERGY?<br>*Number of days*	text (integer, Min: 0, Max: 30)
	394	health_related_quality_of_life_complete	Section Header: *Form Status*<br>Complete?	dropdown<br>0 Incomplete<br>1 Unverified<br>2 Complete

Instrument: **Opioid Use Stigma Scale** (opioid_use_stigma_scale) Collapse

	395	bdlabel	Interviewer read: This first set of statements asks about some of your experiences, feelings, and opinions about those who use heroin or other opiate drugs, and have a substance use disorder. Please do your best to answer each statement whether you: [Show CARD] 1=Strongly disagree; 2=disagree; 3=Agree; or 4=Strongly agree. [SELECT ONE RESPONSE FOR EACH LINE]	descriptive<br>Field Annotation: @HIDDEN
	396	bd1	BD1. People's attitudes make me feel worse about myself. U, K: bd1ru	radio (Matrix)<br>1 1 Strongly Disagree<br>2 2 Disagree<br>3 3 Agree<br>4 4 Strongly Agree
	397	bd2	BD2. People who use heroin or opiate drugs lose jobs when their employers find out. U, K:bd2ru	radio (Matrix)<br>1 1 Strongly Disagree<br>2 2 Disagree<br>3 3 Agree<br>4 4 Strongly Agree
	398	bd3	BD3. I feel I'm not as good a person as others because I use heroin or opiate drugs U, K: bd3ru	radio (Matrix)<br>1 1 Strongly Disagree<br>2 2 Disagree<br>3 3 Agree<br>4 4 Strongly Agree

	399	bd4	BD4. Most people think a person who uses heroin or opiate drugs is dirty U, K:bd4ru	radio (Matrix)<br>1 | 1 Strongly Disagree<br>2 | 2 Disagree<br>3 | 3 Agree<br>4 | 4 Strongly Agree
	400	bd5	BD5. Using heroin or opiate drugs makes me feel unclean U, K: bd5ru	radio (Matrix)<br>1 | 1 Strongly Disagree<br>2 | 2 Disagree<br>3 | 3 Agree<br>4 | 4 Strongly Agree
	401	bd6	BD6. Since learning I have a substance use disorder, I feel set apart and isolated from the rest of the world U, K:bd6ru	radio (Matrix)<br>1 | 1 Strongly Disagree<br>2 | 2 Disagree<br>3 | 3 Agree<br>4 | 4 Strongly Agree
	402	bd7	BD7. Most people think a person with a substance use disorder is disgusting U, K:bd7ru	radio (Matrix)<br>1 | 1 Strongly Disagree<br>2 | 2 Disagree<br>3 | 3 Agree<br>4 | 4 Strongly Agree
	403	bd8	BD8. Having a substance use disorder makes me feel like I'm a bad person U, K:bd8ru	radio (Matrix)<br>1 | 1 Strongly Disagree<br>2 | 2 Disagree<br>3 | 3 Agree<br>4 | 4 Strongly Agree
	404	bd9	BD9. Most people with a substance use disorder are rejected when others find out U, K:bd9ru	radio (Matrix)<br>1 | 1 Strongly Disagree<br>2 | 2 Disagree<br>3 | 3 Agree<br>4 | 4 Strongly Agree
	405	bd10	BD10. Some people who know I use heroin or opiate drugs have grown more distant U, K: bd10ru	radio (Matrix)<br>1 | 1 Strongly Disagree<br>2 | 2 Disagree<br>3 | 3 Agree<br>4 | 4 Strongly Agree
	406	bd11	BD11. I worry that people may judge me when they learn I use heroin or opiate drugs U, K:bd11ru	radio (Matrix)<br>1 | 1 Strongly Disagree<br>2 | 2 Disagree<br>3 | 3 Agree<br>4 | 4 Strongly Agree
	407	bd12	BD12. I have been hurt by how people have reacted when learning I use heroin or opiate drugs U, K:bd12ru	radio (Matrix)<br>1 | 1 Strongly Disagree<br>2 | 2 Disagree<br>3 | 3 Agree<br>4 | 4 Strongly Agree

	408	bd13	BD13. I worry people who know I use heroin or opiate drugs will tell others U, K: bd13ru	radio (Matrix)<br>1 1 Strongly Disagree<br>2 2 Disagree<br>3 3 Agree<br>4 4 Strongly Agree
	409	bd14	BD14. I regret having told some people that I used heroin or opiate drugs U, K:bd14ru	radio (Matrix)<br>1 1 Strongly Disagree<br>2 2 Disagree<br>3 3 Agree<br>4 4 Strongly Agree
	410	bd15	BD15. Some people avoid touching me if they know I use heroin or opiate drugs U, K:bd15ru	radio (Matrix)<br>1 1 Strongly Disagree<br>2 2 Disagree<br>3 3 Agree<br>4 4 Strongly Agree
	411	bd16	BD16. People I care about stopped calling after learning I use heroin or opiate drugs U, K: bd16ru	radio (Matrix)<br>1 1 Strongly Disagree<br>2 2 Disagree<br>3 3 Agree<br>4 4 Strongly Agree
	412	bd17	BD17. Some people close to me are afraid others will reject them if it becomes known that I use heroin or opiate drugs U, K:bd17ru	radio (Matrix)<br>1 1 Strongly Disagree<br>2 2 Disagree<br>3 3 Agree<br>4 4 Strongly Agree
	413	bd18	BD18. People don't want me around their children once they know I use heroin or opiate drugs U, K:bd18ru	radio (Matrix)<br>1 1 Strongly Disagree<br>2 2 Disagree<br>3 3 Agree<br>4 4 Strongly Agree
	414	bd19	BD19. People have physically backed away from me when they learned I use heroin or opiate drugs U, K:bd19ru	radio (Matrix)<br>1 1 Strongly Disagree<br>2 2 Disagree<br>3 3 Agree<br>4 4 Strongly Agree
	415	bd20	BD20. Some people act as though it's my fault I have a substance use disorder U, K:bd20ru	radio (Matrix)<br>1 1 Strongly Disagree<br>2 2 Disagree<br>3 3 Agree<br>4 4 Strongly Agree
	416	bd21	BD21. I have lost friends by telling them I use heroin or opiate drugs U, K:bd21ru	radio (Matrix)<br>1 1 Strongly Disagree<br>2 2 Disagree<br>3 3 Agree<br>4 4 Strongly Agree

	417	bd22	BD22. People who know I use heroin or opiate drugs tend to ignore my good points U, K:bd22ru	radio (Matrix)<br>1 1 Strongly Disagree<br>2 2 Disagree<br>3 3 Agree<br>4 4 Strongly Agree
	418	bd23	BD23. People seem afraid of me when they learn I use heroin or opiate drugs U, K: bd23ru	radio (Matrix)<br>1 1 Strongly Disagree<br>2 2 Disagree<br>3 3 Agree<br>4 4 Strongly Agree
	419	opioid_use_stigma_scale_complete	Section Header: *Form Status*<br>Complete?	dropdown<br>0 Incomplete<br>1 Unverified<br>2 Complete

Instrument: **CES-D 10** (cesd_10) Collapse

	420	script_cesd	For each of the following statements, please choose the option that best describes how often you felt or behaved this way during the past week.<br><br>Number of days in the past week:	descriptive
	421	cd1	1. You were bothered by things that usually don't bother you U, K:cd1ru	radio (Matrix)<br>0 Rarely or none of the time (less than 1 day)<br>1 Some or a little of the time (1-2 days)<br>2 Occasionally or a moderate amount of the time(3-4 days)<br>3 Most or all of the time (5-7 days)
	422	cd2	2. You had trouble keeping your mind on what you were doing U, K:cd2ru	radio (Matrix)<br>0 Rarely or none of the time (less than 1 day)<br>1 Some or a little of the time (1-2 days)<br>2 Occasionally or a moderate amount of the time(3-4 days)<br>3 Most or all of the time (5-7 days)
	423	cd3	3. You felt depressed U, K:cd3ru	radio (Matrix)<br>0 Rarely or none of the time (less than 1 day)<br>1 Some or a little of the time (1-2 days)<br>2 Occasionally or a moderate amount of the time(3-4 days)<br>3 Most or all of the time (5-7 days)
	424	cd4	4. You felt that everything you did was an effort U, K:cd4ru	radio (Matrix)<br>0 Rarely or none of the time (less than 1 day)<br>1 Some or a little of the time (1-2 days)<br>2 Occasionally or a moderate amount of the time(3-4 days)<br>3 Most or all of the time (5-7 days)
	425	cd5	5. You felt hopeful about the future U, K:cd5ru	radio (Matrix)<br>0 Rarely or none of the time (less than 1 day)<br>1 Some or a little of the time (1-2 days)<br>2 Occasionally or a moderate amount of the time(3-4 days)<br>3 Most or all of the time (5-7 days)

	426	cd6	6. You felt fearful U, K:cd6ru	radio (Matrix)<br>0 Rarely or none of the time (less than 1 day)<br>1 Some or a little of the time (1-2 days)<br>2 Occasionally or a moderate amount of the time(3-4 days)<br>3 Most or all of the time (5-7 days)
	427	cd7	7. Your sleep was restless U, K:cd7ru	radio (Matrix)<br>0 Rarely or none of the time (less than 1 day)<br>1 Some or a little of the time (1-2 days)<br>2 Occasionally or a moderate amount of the time(3-4 days)<br>3 Most or all of the time (5-7 days)
	428	cd8	8. You were happy U, K:cd8ru	radio (Matrix)<br>0 Rarely or none of the time (less than 1 day)<br>1 Some or a little of the time (1-2 days)<br>2 Occasionally or a moderate amount of the time(3-4 days)<br>3 Most or all of the time (5-7 days)
	429	cd9	9. You felt lonely U, K:cd9ru	radio (Matrix)<br>0 Rarely or none of the time (less than 1 day)<br>1 Some or a little of the time (1-2 days)<br>2 Occasionally or a moderate amount of the time(3-4 days)<br>3 Most or all of the time (5-7 days)
	430	cd10	10. You could not get "going" U, K:cd10ru	radio (Matrix)<br>0 Rarely or none of the time (less than 1 day)<br>1 Some or a little of the time (1-2 days)<br>2 Occasionally or a moderate amount of the time(3-4 days)<br>3 Most or all of the time (5-7 days)
	431	cesd_10_complete	Section Header: *Form Status*<br>Complete?	dropdown<br>0 Incomplete<br>1 Unverified<br>2 Complete

Instrument: **AUDIT** (audit) Collapse

	432	auditlabel	The next series of questions are going to ask you alcohol consumption related questions. By alcohol consumption we mean your use of wine, beer, vodka (or any other spirit, like cognac, braga). In case you do not drink specific beverage, choose the smallest portion. This information will be used only by the research team and will not be shared with any program staff.<br><br>One drink is equivalent to 40 ml of strong spirits, 140ml of wine, or 330ml of beer	descriptive<br>Field Annotation: @HIDDEN

	433	audit1	1. How often did you have a drink containing alcohol? U, K:audit1ru	radio<br>0 Never<br>1 Monthly or less<br>2 2 to 4 times a month<br>3 2 to 3 times a week<br>4 4 or more times a week<br><br>Field Annotation: @HIDDEN
	434	audit2	2. How many units of alcohol do you drink on a typical day when you are drinking? U, K:audit2ru	radio<br>0 1-2<br>1 3-4<br>2 5-6<br>3 7-8<br>4 10+<br><br>Field Annotation: @HIDDEN
	435	audit3	3. How often have you had 6 or more units if female, or 8 or more if male, on a single occasion in the last year? U, K:audit3ru	radio<br>0 Never<br>1 Less than monthly<br>2 Monthly<br>3 Weekly<br>4 Daily or almost daily<br><br>Field Annotation: @HIDDEN
	436	audit11	Audit Total Score	calc<br>Calculation: [audit1]+[audit2]+[audit3]<br>Field Annotation: @HIDDEN
	437	audit_complete	Section Header: *Form Status*<br>Complete?	dropdown<br>0 Incomplete<br>1 Unverified<br>2 Complete

Instrument: **Sex Risk Behavior** (sex_risk_behavior) Collapse

	438	sxlabel	This section is going to ask you sensitive questions about your sexual activity in the last 90 days (3 months). The information you provide is solely being used for research purposes by our research team and will not be linked to your name. This information is strictly confidential. Please be as honest as possible.	descriptive
	439	sx1	1. Have you EVER exchanged sex for any type of material reward? U, K:sx1ru	radio<br>1 Yes<br>0 No
	440	sx2	2. Have you EVER been forced to have sex against your will? U, K:sx2ru	radio<br>1 Yes<br>0 No
	441	sx3	3. Have you had sex (vaginal, anal) with anyone in the last 90 days? U, K:sx3ru	radio<br>1 Yes<br>0 No
	442	sxlabel2	The following questions ask with whom you have had sex in the past 90 days and whether you used condoms during sexual contact with these partners	descriptive

	443	sx4	4. In the past 90 days, have you exchanged sex for any type of material reward? U, K:sx4ru	radio<br>1 Yes<br>0 No
	444	sx5	5. In the past 90 days, how many sexual partners have you had either vaginal or anal sex with? U, K:sx5ru<br>*partners*	text (integer, Min: 1, Max: 30)
	445	sx6	6. Among these sexual partners over the past 90 days, about how many of them did you have vaginal or anal sex WITHOUT a condom? U, K:sx6ru<br>*partners*	text (integer, Min: 0, Max: 30)
	446	sx5_ctrl<br>Show the field ONLY if:<br>[sx6] > [sx5]	Warning: the value entered is not consistent with previous answers. Please check your answer.	descriptive
	447	sx7	7. Now I want you to think about the partners in the past 90 days that you had UNPROTECTED vaginal or anal sex (no condom), please check how you would describe any of these partners: U, K:sx7ru	checkbox<br>1 sx7___1 At least one of these partners was HIV+<br>2 sx7___2 At least one of these partners was someone with whom I exchanged money (paid for it or got paid for it)<br>3 sx7___3 Was my regular partner that I have been having sex with for a long time (not a paying partner)<br>4 sx7___4 Was a casual partner that I would not describe as a regular partner<br>5 sx7___5 Was a sexual partner that I also use drugs with
	448	sx8	8. During the last time you had sex (anal or vaginal) did you use condom? U, K:sx8ru	radio<br>1 Yes<br>0 No
	449	sex_risk_behavior_complete	Section Header: *Form Status*<br>Complete?	dropdown<br>0 Incomplete<br>1 Unverified<br>2 Complete

Instrument: **Hiv Comorbidities** (hiv_comorbidities) Collapse

	450	hv1a	1a. Have you ever had an HIV test? U, K:hv1aru, hv1aruf1	radio<br>1 Yes<br>0 No<br>8 Refused to Answer
	451	hv2am	2a. When were you last tested for HIV? Month U, K:hv2amru hv2amruf1<br>*Month*	text (integer, Min: 1, Max: 12)
	452	hv2ay	2a. Year U, K:hv1ayru, hv2ayruf1<br>*Year*	text (number, Min: 1985, Max: 2020)
	453	hv1	1. What was the result of your last HIV test? U, K:hv1ru, hv1ruf1	radio<br>1 I was told I was HIV positive (that I have HIV)<br>2 I was told I was HIV negative (that I do NOT have HIV)<br>3 I did not go back and get the results of my tests
	454	hv2	2. Have you undergone confirmatory testing in a medical institution/department where they drew your blood and confirmed your HIV test using second test? U, K:hv2ru, hv2ruf1	radio<br>1 Yes<br>0 No

	455	hv3	3. Did you go back to get the results? U, K:hv3ru, hv3ruf1	radio<br>1 Yes<br>0 No
	456	hv4	4. Are you a "registered" patient at the AIDS center U, , K:hv4ru, hv4ruf1	yesno<br>1 Yes<br>0 No
	457	hv5m	5. When was the last time you had your CD4 checked? Month U, K:hv5mru, hv5mruf1<br>*Month*	text (integer, Min: 1, Max: 12)
	458	hv5y	5. Year U, K:hv5yru, hv5yruf1<br>*Year*	text (integer, Min: 1985, Max: 2020)
	459	hv6	6. Which of the following responses represents your most recent CD4 count? U, K:hv6ru, hv6ruf1	radio<br>1 I never went back to get my results<br>2 I went back for the results and was told I need HIV medicines<br>3 I went back for results and was told I did not need HIV medicines<br>4 The doctors didn't comment on HIV medications<br>5 I don't remember
	460	hv7	7. What was the result of your last CD4 count? Approximate if needed.If you do not remember, enter 777. U:hv7ru hv7ruf1, K: N/A	text (number)
	461	hv8	8. Did you start taking ART medication? U, K:hv8ru, hv8ruf1	radio<br>1 Yes<br>0 No
	462	hv8a	8a. On a scale of 1-10 , how important is it for you to start HIV medications? U, K:hv8aru, hv8aruf1	radio<br>1 1-not important at all<br>2 2<br>3 3<br>4 4<br>5 5<br>6 6<br>7 7<br>8 8<br>9 9<br>10 10-extremely important<br><br>Custom alignment: LH
	463	hv9	9. Are you currently taking ART medication? U, K:hv9ru, hv9ruf1	yesno<br>1 Yes<br>0 No

	464	hv10	10 On a scale of 1-10, how confident are you that you can get a doctor to prescribe you HIV medications now? U, K:hv10ru, hv10ruf1	radio<br>1 1 - not confident at all<br>2 2<br>3 3<br>4 4<br>5 5<br>6 6<br>7 7<br>8 8<br>9 9<br>10 10 - extremely confident<br>Custom alignment: LH
	465	hv11	11. It is often difficult to remember to take all of your medications exactly as prescribed by your doctor. Please, use the following sliding scale and place the mark approximately with how you believe that you have been able to take the medications over the past 30 days. (0% means you missed all prescribed doses, 100% means you NEVER missed a dose). (movable bar) U, K: hv11ru, hv11ruf1	slider<br>Slider labels: 0%-None of the time, 50%-About half the time, 100% All of the time<br>Custom alignment: RV
	466	hiv_comorbidities_complete	Section Header: *Form Status*<br>Complete?	dropdown<br>0 Incomplete<br>1 Unverified<br>2 Complete
Instrument: **HIV Comorbidities Part 2** (hiv_comorbidities_part_2) Collapse				
	467	cd6label	The next series of questions are going to ask you about different aspects of your health. This information will be used only by the research team. Please be as honest as possible	descriptive
	468	cd6a<br>Show the field ONLY if:<br>[event] = '1' or [event] = '2'	6a. Have you ever been told by a medical professional that you have tuberculosis? U, K:cd6aru	yesno<br>1 Yes<br>0 No
	469	cd6a_2<br>Show the field ONLY if:<br>[event] = '3' or [event] = '4' or [event] = '5'	6a. In the last 30 days have you been told by a medical professional that you have tuberculosis? U, K:cd6aruf1	yesno<br>1 Yes<br>0 No
	470	cd6bm<br>Show the field ONLY if:<br>[cd6a] = '1' or [cd6a_2] = '1'	6b. When were you told you had tuberculosis? Month U, K:cd6bmru, cd6bmruf1<br>*MM*	text (integer, Min: 1, Max: 12)
	471	cd6by<br>Show the field ONLY if:<br>[cd6a] = '1' or [cd6a_2] = '1'	6b. Year U, K:cd6byru, cd6byruf1<br>*YYYY*	text (integer, Min: 1917, Max: 2020)
	472	cd6c<br>Show the field ONLY if:<br>[cd6a] = '1' or [cd6a_2] = '1'	CD6c. Did you receive treatment? U, K: cd6cru, cd6cruf1	yesno<br>1 Yes<br>0 No
	473	cd10a<br>Show the field ONLY if:<br>[event] = '1' or [event] = '2'	10a. Have you ever been told by a medical professional that you have syphilis? U, K: cd10aru	yesno<br>1 Yes<br>0 No
	474	cd10a_2<br>Show the field ONLY if:<br>[event] = '3' or [event] = '4' or [event] = '5'	10a.In the last 30 days have you been told by a medical professional that you have syphilis? U, K: cd10aruf1	yesno<br>1 Yes<br>0 No

	475	cd10bm<br><br>Show the field ONLY if:<br>[cd10a] = '1' or [cd10a_2] = '1'	10b. When were you told you had syphilis? Month U, K: cd10bmru<br>*MM*	text (integer, Min: 1, Max: 12)
	476	cd10by<br><br>Show the field ONLY if:<br>[cd10a] = '1' or [cd10a_2] = '1'	10b. Year U, K:cd10byru<br>*YYYY*	text (integer, Min: 1917, Max: 2020)
	477	cd10c<br><br>Show the field ONLY if:<br>[cd10a] = '1' or [cd10a_2] = '1'	10c. Did you receive treatment? U, K:cd10cru	yesno<br>1 Yes<br>0 No
	478	cd11a<br><br>Show the field ONLY if:<br>[event] = '1' or [event] = '2'	11a. Have you ever been told by a medical professional that you have other sexually transmitted infections? U, K: cd11aru	yesno<br>1 Yes<br>0 No
	479	cd11a_2	11a. In the last 30 days have you been told by a medical professional that you have other sexually transmitted infections?U, K: cd11aruf1	yesno<br>1 Yes<br>0 No
	480	cd11b<br><br>Show the field ONLY if:<br>[cd11a] = '1' or [cd11a_2] = '1'	11b. Please, specify the other sexually transmitted infections U, K: cd11bru, cd11bruf1	notes
	481	cd11cm<br><br>Show the field ONLY if:<br>[cd11a] = '1' or [cd11a_2] = '1'	11c. When was the last time you had this STI? Month U, K: cd11cmru, cd11cmruf1<br>*MM*	text (integer, Min: 1, Max: 12)
	482	cd11cy<br><br>Show the field ONLY if:<br>[cd11a] = '1' or [cd11a_2] = '1'	11c. Year U, K: cd11cyru, cd11cyruf1 M: cd11cyru, cd11com<br>*YYYY*	text (number, Min: 1917, Max: 2020)
	483	cd11d<br><br>Show the field ONLY if:<br>[cd11a] = '1' or [cd11a_2] = '1'	CD11d. Did you get treatment for this STI? U, K: cd11dru, cd11druf1	yesno<br>1 Yes<br>0 No
	484	hiv_comorbidities_part_2_complete	Section Header: *Form Status*<br>Complete?	dropdown<br>0 Incomplete<br>1 Unverified<br>2 Complete

Instrument: **Detention History** (detention_history) Collapse

	485	dh11	DH11. In the last year, approximately how many times had you been UNOFFICIALLY detained by the police? Enter "0" times if you had not been officially detained.(Note: an official detention is any detention where formal charges are brought against you). U, K:dh11ru	text (number, Min: 0, Max: 20)
	486	dh11b<br><br>Show the field ONLY if:<br>[dh11] >= 1	DH11b. During the [dh11] UNOFFICIAL detentions by the police in the last year, how many times did you experience any symptoms of abstinence syndrome?U , K:dh11bru1<br>*time(s)*	text (number, Min: 0, Max: 20)
	487	dh11d<br><br>Show the field ONLY if:<br>[dh11] >= 1	DH11d. During the [Response to DH11] UNOFFICIAL detentions by the police in the last year, how many times were HIV medications interrupted for more than 24 hours? If you were not taking HIV medication in the year before incarceration, select "Not Applicable". U:dh11dru1	text (number, Min: 0, Max: 20)
	488	dh11f<br><br>Show the field ONLY if:<br>[dh11] >= 1	DH11f. During any of the [dh11] UNOFFICIAL detentions by the police in the last year, how many times was methadone interrupted for more than 24 hours? U, K:dh11fru1	text (number, Min: 0, Max: 20)
	489	dh11h<br><br>Show the field ONLY if:<br>[dh11] >= 1	DH11h. During any of the [dh11] UNOFFICIAL detentions by the police in the last year, how many times was your drug use, access to HIV or TB medications or access to substitution therapy used to extract a confession? U, K:dh11hru	text (number, Min: 0, Max: 20)

	490	dh11k1<br><br>Show the field ONLY if:<br>[dh11] >= 1	DH11k1. During the [Response to DH11] UNOFFICIAL detention(s) in the last year, were you able to see a medical provider if you needed to?If you did not need to, select "Not Applicable". U, K:dh11k1ru1	radio<br>1 Yes<br>0 No<br>8 Refuse to Answer<br>9 Not Applicable
	491	dh11a	DH11a. In the last year, approximately how many times had you been OFFICIALLY detained by the police? Enter "0" times if you had not been officially detained.(Note: an official detention is any detention where formal charges are brought against you). U, K:dh11aru	text (number, Min: 0, Max: 20)
	492	dh11c<br><br>Show the field ONLY if:<br>[dh11a] >= 1	DH11c. During any of the [dh11a] OFFICIAL detentions by the police in the last year, how many times did you experience any symptoms of abstinence syndrome? U:dh11cru1<br>*time(s)*	text (number, Min: 0, Max: 20)
	493	dh11e<br><br>Show the field ONLY if:<br>[dh11a] >= 1	DH11e. During the [Response to DH11a] OFFICIAL detentions by the police in the last year, how many times were HIV medications interrupted for more than 24 hours? If you were not taking HIV medication in the year before incarceration, select "Not Applicable". U:dh11eru1	text (number, Min: 0, Max: 20)
	494	dh11g<br><br>Show the field ONLY if:<br>[dh11a] >= 1	DH11g. During any of the [Response to DH11a] OFFICIAL detentions by the police in the last year, how many times was methadone interrupted for more than 24 hours? If you were not taking methadone in the year before incarceration, select "Not Applicable". U, K:dh11gru	text (number, Min: 0, Max: 20)
	495	dh11i<br><br>Show the field ONLY if:<br>[dh11a] >= 1	DH11i. During any of the [dh11a] OFFICIAL detentions by the police in the last year, how many times was your drug use, access to HIV or TB medications or access to substitution therapy used to extract a confession? U, K:dh11iru	text (number, Min: 0, Max: 20)
	496	dh11k2<br><br>Show the field ONLY if:<br>[dh11a] >= 1	DH11k2. During the [dh11a] OFFICIAL detention(s) in the last year, were you able to see a medical provider if you needed to?<br>U, K: DH11k2ru	radio<br>1 Yes<br>0 No<br>8 Refuse to Answer<br>9 Not Applicable<br><br>Field Annotation: @HIDDEN
	497	dh11j	DH11j. How many times were you stopped by the police and searched, held, or arrested as you traveled to or from a needle exchange site in the last year? K: dh11jru	text (number, Min: 0, Max: 20)<br>Field Annotation: @HIDDEN
	498	detention_history_complete	Section Header: *Form Status*<br>Complete?	dropdown<br>0 Incomplete<br>1 Unverified<br>2 Complete

Instrument: **Social Support** (social_support) Collapse

	499	sslabel	For the next set of statements, please give information on your social support. People sometimes look to others for companionship, assistance, or other types of support. Please answer using the following scale: [None of the time, A little of the time, Some of the time, Most of the time, All of the time].<br><br>How often is each of the following kinds of support available to you if you need it?<br>*Interviewer: If the person says they didn't need it, ask "So you would say so none of the time or a little of the time?" and mark appropriate response.*	descriptive

	500	ss1	Someone to help if you were confined to bed U, K:ss1ru	radio (Matrix)<br>1 None of the time<br>2 A little of the time<br>3 Some of the time<br>4 Most of the time<br>5 All of the time
	501	ss2	Someone you can count on to listen to you when you talk U, K:ss2ru	radio (Matrix)<br>1 None of the time<br>2 A little of the time<br>3 Some of the time<br>4 Most of the time<br>5 All of the time
	502	ss3	Someone to give you advice about a crisis U, K:ss3ru	radio (Matrix)<br>1 None of the time<br>2 A little of the time<br>3 Some of the time<br>4 Most of the time<br>5 All of the time
	503	ss4	Someone to take you to the doctor if you needed it U, K:ss4ru	radio (Matrix)<br>1 None of the time<br>2 A little of the time<br>3 Some of the time<br>4 Most of the time<br>5 All of the time
	504	ss5	Someone who shows you love and affection U, K:ss5ru	radio (Matrix)<br>1 None of the time<br>2 A little of the time<br>3 Some of the time<br>4 Most of the time<br>5 All of the time
	505	ss6	Someone to have a good time with U, K:ss6ru	radio (Matrix)<br>1 None of the time<br>2 A little of the time<br>3 Some of the time<br>4 Most of the time<br>5 All of the time
	506	ss7	Someone to give you information to help you understand a situation U, K:ss7ru	radio (Matrix)<br>1 None of the time<br>2 A little of the time<br>3 Some of the time<br>4 Most of the time<br>5 All of the time
	507	ss8	Someone to confide in or talk to about yourself or your problems U, K:ss8ru	radio (Matrix)<br>1 None of the time<br>2 A little of the time<br>3 Some of the time<br>4 Most of the time<br>5 All of the time

	508	ss9	Someone who hugs you U, K:ss9ru	radio (Matrix)<br>1 None of the time<br>2 A little of the time<br>3 Some of the time<br>4 Most of the time<br>5 All of the time
	509	ss10	Someone to get together with for relaxation U, K:ss10ru	radio (Matrix)<br>1 None of the time<br>2 A little of the time<br>3 Some of the time<br>4 Most of the time<br>5 All of the time
	510	ss11	Someone to prepare your meals if you were unable to do it yourself U, K:ss11ru	radio (Matrix)<br>1 None of the time<br>2 A little of the time<br>3 Some of the time<br>4 Most of the time<br>5 All of the time
	511	ss12	Someone whose advice you really want U, K:ss12ru	radio (Matrix)<br>1 None of the time<br>2 A little of the time<br>3 Some of the time<br>4 Most of the time<br>5 All of the time
	512	ss13	Someone to do things with to get your mind off things U, K:ss13ru	radio (Matrix)<br>1 None of the time<br>2 A little of the time<br>3 Some of the time<br>4 Most of the time<br>5 All of the time
	513	ss14	Someone to help with daily chores if you were sick U, K:ss14ru	radio (Matrix)<br>1 None of the time<br>2 A little of the time<br>3 Some of the time<br>4 Most of the time<br>5 All of the time
	514	ss15	Someone to share your most private worries and fears with U, K:ss15ru	radio (Matrix)<br>1 None of the time<br>2 A little of the time<br>3 Some of the time<br>4 Most of the time<br>5 All of the time
	515	ss16	Someone to turn to for suggestions about how to deal with personal problems U, K:ss16ru	radio (Matrix)<br>1 None of the time<br>2 A little of the time<br>3 Some of the time<br>4 Most of the time<br>5 All of the time

	516	ss17	Someone to do something enjoyable with U, K:ss17ru	radio (Matrix)<br>1 None of the time<br>2 A little of the time<br>3 Some of the time<br>4 Most of the time<br>5 All of the time
	517	ss18	Someone to understand your problems U, K:ss18ru	radio (Matrix)<br>1 None of the time<br>2 A little of the time<br>3 Some of the time<br>4 Most of the time<br>5 All of the time
	518	ss19	Someone to love and make you feel wanted U, K:ss19ru	radio (Matrix)<br>1 None of the time<br>2 A little of the time<br>3 Some of the time<br>4 Most of the time<br>5 All of the time
	519	ss20	20. About how many close friends and close relatives do you have (i.e. people you feel at ease with and can talk to about what is on your mind)? U, K:ss20ru	text (number, Min: 0, Max: 20)
	520	ss21a	21a. On a scale of 1 to 10 where 1 is not at all and 10 is VERY supportive, how would you describe the level of support the person who provides you support has toward you being/entering OST? U, K:ss21aru	radio<br>1 1-not at all<br>2 2<br>3 3<br>4 4<br>5 5<br>6 6<br>7 7<br>8 8<br>9 9<br>10 10 - VERY supportive<br>0 Not Applicable(Kyr)
	521	ss21b<br>Show the field ONLY if:<br>[screen_arm_1][country] = '3'	21b. Did you get a suspended sentence before? U, K:ss21bru	yesno<br>1 Yes<br>0 No
	522	ss22<br>Show the field ONLY if:<br>[screen_arm_1][country] = '3'	22. Do you receive any social support from probation officers:ss22ru, K: N/A	yesno<br>1 Yes<br>0 No
	523	ss22a<br>Show the field ONLY if:<br>[screen_arm_1][country] = '3'	22a. How much social support do you get?U:ss22aru, K: N/A	radio, Required<br>1 Very little<br>2 a bit<br>3 A moderate amount<br>4 More than fair amount<br>5 A lot

	524	ss23<br><br>Show the field ONLY if:<br>[screen_arm_1][country] = '3'	23. What kinds of support you would like to get from probation officers?U:ss23ru, K: N/A	radio<br>1 employment assistance<br>2 assistance in obtaining health care<br>3 legal advice<br>4 psychological/social support for the problems of family relationships<br>5 other (specify) ________
	525	ss23oth<br><br>Show the field ONLY if:<br>[ss23] = '5'	23. Other (specify)	text
	526	ss24<br><br>Show the field ONLY if:<br>[screen_arm_1][country] = '3'	24. Would you like a probation officer to help you in obtaining treatment for opioid dependence? U:ss24ru	radio<br>1 Yes<br>0 No
	527	ss24a<br><br>Show the field ONLY if:<br>[screen_arm_1][country] = '3'	24a. What category of probation officers would you prefer to get help from?U:ss24aru	radio<br>1 Lawyer<br>2 Social worker<br>3 Other (specify) ______
	528	ss24aoth<br><br>Show the field ONLY if:<br>[screen_arm_1][country] = '3'	24. Other(specify) U:ss24aothru	text
	529	social_support_complete	Section Header: *Form Status*<br>Complete?	dropdown<br>0 Incomplete<br>1 Unverified<br>2 Complete

Instrument: **IMB** (imb) Collapse

	530	imb1<br><br>Show the field ONLY if:<br>[ma6]>0	Section Header: *INFORMATION*<br>I know how methadone is supposed to be taken (for example whether or not it can be taken with food, herbal supplements, or other prescription medications). U, K:imb1ru	radio (Matrix)<br>1 strongly disagree<br>2 somewhat disagree<br>3 neither agree nor disagree<br>4 somewhat agree<br>5 strongly agree<br>9 Not Applicable(Kyr only)
	531	imb2<br><br>Show the field ONLY if:<br>[ma6]>0	I know what to do if I miss a dose of methadone U, K:imb2ru	radio (Matrix)<br>1 strongly disagree<br>2 somewhat disagree<br>3 neither agree nor disagree<br>4 somewhat agree<br>5 strongly agree<br>9 Not Applicable(Kyr only)
	532	imb3<br><br>Show the field ONLY if:<br>[ma6]>0	Skipping a few of my methadone doses from time to time would not really hurt my health. U, K:imb3ru	radio (Matrix)<br>1 strongly disagree<br>2 somewhat disagree<br>3 neither agree nor disagree<br>4 somewhat agree<br>5 strongly agree<br>9 Not Applicable(Kyr only)

	533	imb4<br><br>Show the field ONLY if:<br>[ma6]>0	I know what the possible side effects of methadone are. U, K:imb4ru	radio (Matrix)<br>1 strongly disagree<br>2 somewhat disagree<br>3 neither agree nor disagree<br>4 somewhat agree<br>5 strongly agree<br>9 Not Applicable(Kyr only)
	534	imb5<br><br>Show the field ONLY if:<br>[ma6]>0	As long as I am feeling healthy, missing my methadone from time to time is OK. U, K:imb5ru	radio (Matrix)<br>1 strongly disagree<br>2 somewhat disagree<br>3 neither agree nor disagree<br>4 somewhat agree<br>5 strongly agree<br>9 Not Applicable(Kyr only)
	535	imb6<br><br>Show the field ONLY if:<br>[ma6]>0	I understand how methadone works in my body to fight drug addiction. U, K:imb6ru	radio (Matrix)<br>1 strongly disagree<br>2 somewhat disagree<br>3 neither agree nor disagree<br>4 somewhat agree<br>5 strongly agree<br>9 Not Applicable(Kyr only)
	536	imb7<br><br>Show the field ONLY if:<br>[ma6]>0	I believe that if I take methadone as prescribed, I will live longer. U, K:imb7ru	radio (Matrix)<br>1 strongly disagree<br>2 somewhat disagree<br>3 neither agree nor disagree<br>4 somewhat agree<br>5 strongly agree<br>9 Not Applicable(Kyr only)
	537	imb8<br><br>Show the field ONLY if:<br>[ma6]>0	I know how methadone interacts with alcohol and street drugs (6-I don't drink/use drugs) U, K:imb8ru	radio (Matrix)<br>1 strongly disagree<br>2 somewhat disagree<br>3 neither agree nor disagree<br>4 somewhat agree<br>5 strongly agree<br>9 Not Applicable(Kyr only)
	538	imb9<br><br>Show the field ONLY if:<br>[ma6]>0	Section Header: *MOTIVATION*<br>I am worried that other people might realize that I am a drug addict if they see me taking methadone. U, K:imb9ru	radio (Matrix)<br>1 strongly disagree<br>2 somewhat disagree<br>3 neither agree nor disagree<br>4 somewhat agree<br>5 strongly agree<br>9 Not Applicable(Kyr Only)

	539	imb10<br>Show the field ONLY if:<br>[ma6]>0	I get frustrated taking methadone because I have to plan my life around it. U, K:imb10ru	radio (Matrix)<br>1 strongly disagree<br>2 somewhat disagree<br>3 neither agree nor disagree<br>4 somewhat agree<br>5 strongly agree<br>9 Not Applicable(Kyr Only)
	540	imb11<br>Show the field ONLY if:<br>[ma6]>0	I don't like taking methadone because it reminds me that I am an addict. U, K:imb11ru	radio (Matrix)<br>1 strongly disagree<br>2 somewhat disagree<br>3 neither agree nor disagree<br>4 somewhat agree<br>5 strongly agree<br>9 Not Applicable(Kyr Only)
	541	imb12<br>Show the field ONLY if:<br>[ma6]>0	I feel that my healthcare provider takes my needs into account when making recommendations about treatment with methadone. U, K:imb12ru	radio (Matrix)<br>1 strongly disagree<br>2 somewhat disagree<br>3 neither agree nor disagree<br>4 somewhat agree<br>5 strongly agree<br>9 Not Applicable(Kyr Only)
	542	imb13<br>Show the field ONLY if:<br>[ma6]>0	Most people who are important to me who know I'm an addict support me in taking methadone. U, K:imb13ru	radio (Matrix)<br>1 strongly disagree<br>2 somewhat disagree<br>3 neither agree nor disagree<br>4 somewhat agree<br>5 strongly agree<br>9 Not Applicable(Kyr Only)
	543	imb14<br>Show the field ONLY if:<br>[ma6]>0	Most people who are important to me who know I'm an addict support me in taking methadone. My healthcare provider doesn't give me enough support when it comes to taking methadone. U, K:imb14ru	radio (Matrix)<br>1 strongly disagree<br>2 somewhat disagree<br>3 neither agree nor disagree<br>4 somewhat agree<br>5 strongly agree<br>9 Not Applicable(Kyr Only)
	544	imb15<br>Show the field ONLY if:<br>[ma6]>0	I am worried that methadone will hurt my health. U, K:imb15ru	radio (Matrix)<br>1 strongly disagree<br>2 somewhat disagree<br>3 neither agree nor disagree<br>4 somewhat agree<br>5 strongly agree<br>9 Not Applicable(Kyr Only)

	545	imb16<br>Show the field ONLY if:<br>[ma6]>0	It upsets me that methadone can affect the way I look. U, K:imb16ru	radio (Matrix)<br>1 strongly disagree<br>2 somewhat disagree<br>3 neither agree nor disagree<br>4 somewhat agree<br>5 strongly agree<br>9 Not Applicable(Kyr Only)
	546	imb17<br>Show the field ONLY if:<br>[ma6]>0	It upsets me that methadone can cause side effects. U, K:imb17ru	radio (Matrix)<br>1 strongly disagree<br>2 somewhat disagree<br>3 neither agree nor disagree<br>4 somewhat agree<br>5 strongly agree<br>9 Not Applicable(Kyr Only)
	547	imb18<br>Show the field ONLY if:<br>[ma6]>0	Section Header: *BEHAVIOR SKILLS*<br>How hard or easy is it for you to stay informed about OST treatment? U, K:imb18ru	radio (Matrix)<br>1 very hard<br>2 hard<br>3 sometimes hard, sometimes easy<br>4 easy<br>5 very easy<br>9 not applicable(Kyr Only)
	548	imb19<br>Show the field ONLY if:<br>[ma6]>0	How hard or easy is it for you to get the support you need from others for taking your methadone (for example, from friends, family, doctor, or pharmacist)? U, K:imb19ru	radio (Matrix)<br>1 very hard<br>2 hard<br>3 sometimes hard, sometimes easy<br>4 easy<br>5 very easy<br>9 not applicable(Kyr Only)
	549	imb20<br>Show the field ONLY if:<br>[ma6]>0	How hard or easy is it for you to get your OST treatment on time? U, K:imb20ru	radio (Matrix)<br>1 very hard<br>2 hard<br>3 sometimes hard, sometimes easy<br>4 easy<br>5 very easy<br>9 not applicable(Kyr Only)
	550	imb21<br>Show the field ONLY if:<br>[ma6]>0	How hard or easy is it for you to take your methadone when you are wrapped up in what you are doing? U, K:imb21ru	radio (Matrix)<br>1 very hard<br>2 hard<br>3 sometimes hard, sometimes easy<br>4 easy<br>5 very easy<br>9 not applicable(Kyr Only)

	551	imb22<br>Show the field ONLY if:<br>[ma6]>0	How hard or easy is it for you to manage the side effects of your methadone treatment? U:imb22ru	radio (Matrix)<br>1 very hard<br>2 hard<br>3 sometimes hard, sometimes easy<br>4 easy<br>5 very easy<br>9 not applicable(Kyr Only)
	552	imb23<br>Show the field ONLY if:<br>[ma6]>0	How hard or easy is it for you to remember to take your methadone? U, K:imb23ru	radio (Matrix)<br>1 very hard<br>2 hard<br>3 sometimes hard, sometimes easy<br>4 easy<br>5 very easy<br>9 not applicable(Kyr Only)
	553	imb24<br>Show the field ONLY if:<br>[ma6]>0	How hard or easy is it for you to make your methadone treatment part of your daily life? U, K:imb24ru	radio (Matrix)<br>1 very hard<br>2 hard<br>3 sometimes hard, sometimes easy<br>4 easy<br>5 very easy<br>9 not applicable(Kyr Only)
	554	imb25<br>Show the field ONLY if:<br>[ma6]>0	How hard or easy is it for you to take your methadone when your usual routine changes (for example, when you traveling)? U, K:imb25ru	radio (Matrix)<br>1 very hard<br>2 hard<br>3 sometimes hard, sometimes easy<br>4 easy<br>5 very easy<br>9 not applicable(Kyr Only)
	555	imb26<br>Show the field ONLY if:<br>[ma6]>0	How hard or easy is it for you to take your methadone when you do not feel good emotionally (for example, when you are depressed, sad, angry, or stressed out)? U, K:imb26ru	radio (Matrix)<br>1 very hard<br>2 hard<br>3 sometimes hard, sometimes easy<br>4 easy<br>5 very easy<br>9 not applicable(Kyr Only)
	556	imb27<br>Show the field ONLY if:<br>[ma6]>0	How hard or easy is it for you to take your methadone when you feel good physically and don't have any symptoms of your addiction? U, K:imb27ru	radio (Matrix)<br>1 very hard<br>2 hard<br>3 sometimes hard, sometimes easy<br>4 easy<br>5 very easy<br>9 not applicable(Kyr Only)

	557	imb28<br><br>Show the field ONLY if:<br>[ma6]>0	How hard or easy is it for you to take your methadone when you do NOT feel good physically? U, K:imb28ru	radio (Matrix)<br>1 very hard<br>2 hard<br>3 sometimes hard, sometimes easy<br>4 easy<br>5 very easy<br>9 not applicable(Kyr Only)
	558	imb29<br><br>Show the field ONLY if:<br>[ma6]>0	How hard or easy is it for you to talk to your health care provider about your methadone treatment? U, , K:imb29ru	radio (Matrix)<br>1 very hard<br>2 hard<br>3 sometimes hard, sometimes easy<br>4 easy<br>5 very easy<br>9 not applicable(Kyr Only)
	559	imb_complete	Section Header: *Form Status*<br><br>Complete?	dropdown<br>0 Incomplete<br>1 Unverified<br>2 Complete

Instrument: **Form 10 And 11 After** (form_10_and_11_after) Collapse

	560	current_on_oat_post	Is the participant currently on OAT? U:onoat_ruf, K: onoat_ru_v2	radio<br>1 Yes<br>0 No
	561	satisfied_treat_length_post<br><br>Show the field ONLY if:<br>[current_on_oat_post] = '1'	1. Are you satisfied with the length of your treatment? (0 = not satisfied, 10 = very satisfied) U:f101ruf, K: f101ru_v2	radio (Matrix)<br>0 0 Not Satisfied<br>1 1<br>2 2<br>3 3<br>4 4<br>5 5<br>6 6<br>7 7<br>8 8<br>9 9<br>10 10 Very Satisfied
	562	satisfied_dose_post<br><br>Show the field ONLY if:<br>[current_on_oat_post] = '1'	2. Are you satisfied with the dose you are receiving? (0 = not satisfied, 10 = very satisfied) U:f102ruf,K: f102ru_v2	radio (Matrix)<br>0 0 Not Satisfied<br>1 1<br>2 2<br>3 3<br>4 4<br>5 5<br>6 6<br>7 7<br>8 8<br>9 9<br>10 10 Very Satisfied

	563	oat_side_effects_post<br><br>Show the field ONLY if:<br>[current_on_oat_post] = '1'	3. How often did you experience side effects from the dose you were taking? (0 = very seldom, 10 = very often) U:f103ruf, K: f103ru_v2	radio (Matrix)<br>0 0 Not Satisfied<br>1 1<br>2 2<br>3 3<br>4 4<br>5 5<br>6 6<br>7 7<br>8 8<br>9 9<br>10 10 Very Satisfied
	564	oat_effectiveness_post<br><br>Show the field ONLY if:<br>[current_on_oat_post] = '1'	4. How effectively does taking methadone or buprenorphine help you reduce other opioid use? (0 = not effective at all, 10 = extremely effective) U:f104ruf , K: f104ru_v2	radio (Matrix)<br>0 0 Not Satisfied<br>1 1<br>2 2<br>3 3<br>4 4<br>5 5<br>6 6<br>7 7<br>8 8<br>9 9<br>10 10 Very Satisfied
	565	oat_qol_increase_post<br><br>Show the field ONLY if:<br>[current_on_oat_post] = '1'	5. How much does treatment with methadone/buprenorphine increase your quality of life? (0 = doesn't help at all, 10 = helps a lot) U:f105ruf, K: f105ru_v2	radio (Matrix)<br>0 0 Not Satisfied<br>1 1<br>2 2<br>3 3<br>4 4<br>5 5<br>6 6<br>7 7<br>8 8<br>9 9<br>10 10 Very Satisfied
	566	oat_relations_improve_post<br><br>Show the field ONLY if:<br>[current_on_oat_post] = '1'	6. How much does treatment with methadone or buprenorphine affect your relationships with friends and family? (0 = doesn't help at all, 10 = helps a lot) U:f106ruf, K: f106ru_v2	radio (Matrix)<br>0 0 Not Satisfied<br>1 1<br>2 2<br>3 3<br>4 4<br>5 5<br>6 6<br>7 7<br>8 8<br>9 9<br>10 10 Very Satisfied

	567	interest_in_oat_post<br><br>Show the field ONLY if:<br>[current_on_oat_post] = '0'	1. How interested are you in receiving OAT? (0 = not interested at all, 10 = very interested) U: f11_1_ruf, K:f11_1_ky_v2	radio (Matrix)<br>0 0<br>1 1<br>2 2<br>3 3<br>4 4<br>5 5<br>6 6<br>7 7<br>8 8<br>9 9<br>10 10
	568	difficulty_start_oat_post<br><br>Show the field ONLY if:<br>[current_on_oat_post] = '0'	2. You said you were interested in receiving OAT. How do difficult do you think it would be for you to start treatment? (0 = not difficult at all, 10 = very difficult) U: f11_2_ruf K:f11_2_ky_v2	radio (Matrix)<br>0 0<br>1 1<br>2 2<br>3 3<br>4 4<br>5 5<br>6 6<br>7 7<br>8 8<br>9 9<br>10 10
	569	importance_receive_oat_post<br><br>Show the field ONLY if:<br>[current_on_oat_post] = '0'	3. How important is it for you to receive OAT? (0 = not important at all, 10 = very important) U: f11_3_ruf K:f11_3_ky_v2	radio (Matrix)<br>0 0<br>1 1<br>2 2<br>3 3<br>4 4<br>5 5<br>6 6<br>7 7<br>8 8<br>9 9<br>10 10
	570	so_instructionsf<br><br>Show the field ONLY if:<br>[current_on_oat_post] = '0'	Please read the following statements carefully. Each one describes a way that<br>you might (or might not) feel about your drug use. For each statement, select one number from 1 to 5, to indicate how much you agree or disagree with it right now. P	descriptive
	571	so1_post<br><br>Show the field ONLY if:<br>[current_on_oat_post] = '0'	Section Header: *Motivation Socrates 8D*<br><br>1. I really want to make changes in my use of drugs. U: So1ruf, K: so1_v2	radio (Matrix)<br>1 1 Absolutely do not agree<br>2 2<br>3 3<br>4 4<br>5 5 Absolutely agree

	572	so2_post<br><br>Show the field ONLY if:<br>[current_on_oat_post] = '0'	2. Sometimes I wonder if I am an addict. U:so2ruf, , K: so2_v2	radio (Matrix)<br>1 1 Absolutely do not agree<br>2 2<br>3 3<br>4 4<br>5 5 Absolutely agree
	573	so3_post<br><br>Show the field ONLY if:<br>[current_on_oat_post] = '0'	3. If I don't change my drug use soon, my problems are going to get worse. U:so3ruf , K: so3_v2	radio (Matrix)<br>1 1 Absolutely do not agree<br>2 2<br>3 3<br>4 4<br>5 5 Absolutely agree
	574	so4_post<br><br>Show the field ONLY if:<br>[current_on_oat_post] = '0'	4. I have already started making some changes in my use of drugs. U:so4ruf, , K: so4_v2	radio (Matrix)<br>1 1 Absolutely do not agree<br>2 2<br>3 3<br>4 4<br>5 5 Absolutely agree
	575	so5_post<br><br>Show the field ONLY if:<br>[current_on_oat_post] = '0'	5. I was using drugs too much at one time, but I've managed to change that. U:so5ruf, , K: so5_v2	radio (Matrix)<br>1 1 Absolutely do not agree<br>2 2<br>3 3<br>4 4<br>5 5 Absolutely agree
	576	so6_post<br><br>Show the field ONLY if:<br>[current_on_oat_post] = '0'	6. Sometimes I wonder if my drug use is hurting other people. U:so6ruf, K: so6_v2	radio (Matrix)<br>1 1 Absolutely do not agree<br>2 2<br>3 3<br>4 4<br>5 5 Absolutely agree
	577	so7_post<br><br>Show the field ONLY if:<br>[current_on_oat_post] = '0'	7. I have a drug problem. U:so7ruf, K: so7_v2	radio (Matrix)<br>1 1 Absolutely do not agree<br>2 2<br>3 3<br>4 4<br>5 5 Absolutely agree
	578	so8_post<br><br>Show the field ONLY if:<br>[current_on_oat_post] = '0'	8. I'm not just thinking about changing my drug use, I'm already doing something about it. U:so8ruf, K: so8_v2	radio (Matrix)<br>1 1 Absolutely do not agree<br>2 2<br>3 3<br>4 4<br>5 5 Absolutely agree
	579	so9_post<br><br>Show the field ONLY if:<br>[current_on_oat_post] = '0'	9. I have already changed my drug use, and I am looking for ways to keep from slipping back to my old pattern. U:so9ruf, K: so9_v2	radio (Matrix)<br>1 1 Absolutely do not agree<br>2 2<br>3 3<br>4 4<br>5 5 Absolutely agree

	580	so10_post<br><br>Show the field ONLY if:<br>[current_on_oat_post] = '0'	10. I have serious problems with drugs. U:so10ruf, K: so10_v2	radio (Matrix)<br>1 1 Absolutely do not agree<br>2 2<br>3 3<br>4 4<br>5 5 Absolutely agree
	581	so11_post<br><br>Show the field ONLY if:<br>[current_on_oat_post] = '0'	11. Sometimes I wonder if I am in control of my drug use. U:so11ruf, , K: so11_v2	radio (Matrix)<br>1 1 Absolutely do not agree<br>2 2<br>3 3<br>4 4<br>5 5 Absolutely agree
	582	so12_post<br><br>Show the field ONLY if:<br>[current_on_oat_post] = '0'	12. My drug use is causing a lot of harm. U:so12ruf, K: so12_v2	radio (Matrix)<br>1 1 Absolutely do not agree<br>2 2<br>3 3<br>4 4<br>5 5 Absolutely agree
	583	so13_post<br><br>Show the field ONLY if:<br>[current_on_oat_post] = '0'	13. I am actively doing things now to cut down or stop my use of drugs. U:so13ruf, K: so13_v2	radio (Matrix)<br>1 1 Absolutely do not agree<br>2 2<br>3 3<br>4 4<br>5 5 Absolutely agree
	584	so14_post<br><br>Show the field ONLY if:<br>[current_on_oat_post] = '0'	14. I want help to keep from going back to the drug problems that I had before. U:so14ruf, K: so14_v2	radio (Matrix)<br>1 1 Absolutely do not agree<br>2 2<br>3 3<br>4 4<br>5 5 Absolutely agree
	585	so15_post<br><br>Show the field ONLY if:<br>[current_on_oat_post] = '0'	15. I know that I have a drug problem. U:so15ruf, K: so15_v2	radio (Matrix)<br>1 1 Absolutely do not agree<br>2 2<br>3 3<br>4 4<br>5 5 Absolutely agree
	586	so16_post<br><br>Show the field ONLY if:<br>[current_on_oat_post] = '0'	16. There are times when I wonder if I use drugs too much. U:so16ruf, K: so16_v2	radio (Matrix)<br>1 1 Absolutely do not agree<br>2 2<br>3 3<br>4 4<br>5 5 Absolutely agree
	587	so17_post<br><br>Show the field ONLY if:<br>[current_on_oat_post] = '0'	17. I am a drug addict. U:so17ruf, K: so17_v2	radio (Matrix)<br>1 1 Absolutely do not agree<br>2 2<br>3 3<br>4 4<br>5 5 Absolutely agree

	588	so18_post<br><br>Show the field ONLY if:<br>[current_on_oat_post] = '0'	18. I am working hard to change my drug use. U:so18ruf, K: so18_v2	radio (Matrix)<br>1 | 1 Absolutely do not agree<br>2 | 2<br>3 | 3<br>4 | 4<br>5 | 5 Absolutely agree
	589	so19_post<br><br>Show the field ONLY if:<br>[current_on_oat_post] = '0'	19. I have made some changes in my drug use, and I want some help to keep from going back to the way I used before. U:so19ruf, K: so19_v2	radio (Matrix)<br>1 | 1 Absolutely do not agree<br>2 | 2<br>3 | 3<br>4 | 4<br>5 | 5 Absolutely agree
	590	form_10_and_11_after_complete	Section Header: *Form Status*<br>Complete?	dropdown<br>0 | Incomplete<br>1 | Unverified<br>2 | Complete

Instrument: **Opioid Test Results** (opioid_test_results) Collapse

	591	toxtestdate<br><br>Show the field ONLY if:<br>[screen_arm_1][country] = '3'	Date of Test U, K:toxtestdate_ru	text (date_mdy)
	592	toxtest_intid<br><br>Show the field ONLY if:<br>[screen_arm_1][country] = '3'	Interviewer code U, K:toxtest_intid_ru	text (number)
	593	toxtestdone<br><br>Show the field ONLY if:<br>[screen_arm_1][country] = '3'	Was the test for opioids performed? U, K:toxtestdone_ru	radio<br>1 | Yes<br>0 | No
	594	toxtest_notdone<br><br>Show the field ONLY if:<br>[screen_arm_1][country] = '3'	If not performed, then why? (write reason for non-consent)U , K: toxtest_notdone_ru	text
	595	toxttest_result<br><br>Show the field ONLY if:<br>[screen_arm_1][country] = '3'	Result: U, K: toxttest_result_ru	radio<br>1 | Positive<br>0 | Negative
	596	coc	Cocaine	radio (Matrix)<br>1 | Positive<br>0 | Negative
	597	tca	tca (?????????????????????????????)	radio (Matrix)<br>1 | Positive<br>0 | Negative
	598	amp	amphetamines	radio (Matrix)<br>1 | Positive<br>0 | Negative
	599	mamp	methamphetamines	radio (Matrix)<br>1 | Positive<br>0 | Negative
	600	thc	Marijuana	radio (Matrix)<br>1 | Positive<br>0 | Negative

	601	mtd	methadone	radio (Matrix)<br>1 Positive<br>0 Negative
	602	opi	opiates	radio (Matrix)<br>1 Positive<br>0 Negative
	603	pcp	phencyclides (PCP)	radio (Matrix)<br>1 Positive<br>0 Negative
	604	bar	barbituates	radio (Matrix)<br>1 Positive<br>0 Negative
	605	bzo	benzodiazepines	radio (Matrix)<br>1 Positive<br>0 Negative
	606	opioid_test_results_complete	Section Header: *Form Status*<br>Complete?	dropdown<br>0 Incomplete<br>1 Unverified<br>2 Complete

Instrument: **Survey Completion Method** (survey_completion_method) Collapse

	607	completion_mthd	How was the survey completed? U, K:compl_ru	radio<br>1 Participant did the survey by themselves without RA assistance<br>2 Occasional assistance from the RA<br>3 RA had to administer most of the interview<br>4 RA had to administer the entire interview
	608	survey_completion_method_complete	Section Header: *Form Status*<br>Complete?	dropdown<br>0 Incomplete<br>1 Unverified<br>2 Complete

Instrument: **Intervention Completion** (intervention_completion) Collapse

	609	intervention	Did you perform the intervention with this participant?	radio<br>1 Yes, I performed and completed the full intervention<br>2 Yes, I performed a partial intervention, it was not complete<br>0 No, I did not get to perform the intervention<br><br>Field Annotation: @HIDDEN
	610	no_interview	Why was the intervention not completed?	notes<br>Field Annotation: @HIDDEN
	611	intervention_completion_complete	Section Header: *Form Status*<br>Complete?	dropdown<br>0 Incomplete<br>1 Unverified<br>2 Complete

Instrument: **Ost Use** (ost_use) Collapse

	612	enrolled	This participant was enrolled on [daya_arm_1][oat_intdt].	descriptive

	613	start_ost	Did this person start OST after the date above?	yesno, Required<br>1 Yes<br>0 No
	614	start_ost_date	OST start date	text (date_ymd)
	615	end_ost_date	OST end date	text (date_mdy)
	616	end_ost_reason	Why did the participant discontinue OST?	radio<br>1 Planned completion of treatment<br>2 Violation of OST patient-provider agreement conditions<br>3 Voluntary refusal<br>4 Other reasons<br>5 Death
	617	end_ost_reason_other<br>Show the field ONLY if: [end_ost_reason] = '4'	Other:	text
	618	ost_use_comments	Comments	notes
	619	start_ost_date_2	Section Header:<br>OST start date 2	text (date_ymd)
	620	end_ost_date_2	OST end date 2	text (date_mdy)
	621	end_ost_reason_2	Why did the participant discontinue OST this second time?	radio<br>1 Planned completion of treatment<br>2 Violation of OST patient-provider agreement conditions<br>3 Voluntary refusal<br>4 Other reasons<br>5 Death
	622	ost_use_comments_2	Comments	notes
	623	ost_use_complete	Section Header: *Form Status*<br>Complete?	dropdown<br>0 Incomplete<br>1 Unverified<br>2 Complete

Instrument: **Form 5** (form_5) Collapse

	624	f5_intvdt	Interview Date<br>U: f5_intvdt_ru	text (date_mdy)
	625	f5_intid	Interviewer ID	text
	626	f5_1	Section Header: *Overall opinion on research*<br>Overall opinion on research	radio (Matrix)<br>1 Yes<br>0 No
	627	f5_2	Views on the organization carrying out the research	radio (Matrix)<br>1 Yes<br>0 No
	628	f5_3	The topic of the study	radio (Matrix)<br>1 Yes<br>0 No
	629	f5_4	Social isolation	radio (Matrix)<br>1 Yes<br>0 No

	630	f5_5	Section Header: *Research Procedures Quality of study instruments:*<br>Content of the interviews	radio (Matrix)<br>1 Yes<br>0 No
	631	f5_6	The way the questions were asked	radio (Matrix)<br>1 Yes<br>0 No
	632	f5_7	The length of the interview	radio (Matrix)<br>1 Yes<br>0 No
	633	f5_8	Work with computers	radio (Matrix)<br>1 Yes<br>0 No
	634	f5_9	The way I was treated	radio (Matrix)<br>1 Yes<br>0 No
	635	f5_10	Section Header: *Research procedures: Interview procedures:*<br>Study methodology	radio (Matrix)<br>1 Yes<br>0 No
	636	f5_11	Follow-up procedures	radio (Matrix)<br>1 Yes<br>0 No
	637	f5_12	Repeated attempts to convince me to continue the study	radio (Matrix)<br>1 Yes<br>0 No
	638	f5_13	Professional characteristics of the interviewer	radio (Matrix)<br>1 Yes<br>0 No
	639	f5_14	The reward for study participation	radio (Matrix)<br>1 Yes<br>0 No
	640	f5_15	Ситуация опроса/интервью на уровне межличностного общения (интервьюер - респондент)	yesno<br>1 Yes<br>0 No
	641	f5_16	Section Header: *Individual, Psychological*<br>A feeling of insecurity when talking to the interviewer	radio (Matrix)<br>1 Yes<br>0 No
	642	f5_17	Fear	radio (Matrix)<br>1 Yes<br>0 No
	643	f5_18	Unsure that information will be kept confidential	radio (Matrix)<br>1 Yes<br>0 No
	644	f5_19	Other social and personal problems	radio (Matrix)<br>1 Yes<br>0 No
	645	f5_20	Doesn't trust interviewer	radio (Matrix)<br>1 Yes<br>0 No

	646	f5_21	Section Header: *Individual Social/demographic characteristics of the interviewer/participant:*<br>Gender	radio (Matrix)<br>1 Yes<br>0 No
	647	f5_22	Age	radio (Matrix)<br>1 Yes<br>0 No
	648	f5_23	Language barriers	radio (Matrix)<br>1 Yes<br>0 No
	649	f5_24	Section Header: *Individual: Other*<br>Didn't feel well	radio (Matrix)<br>1 Yes<br>0 No
	650	f5_25	Transfer to another facility for treatment	radio (Matrix)<br>1 Yes<br>0 No
	651	f5_comments	Comments/Reason for Leaving Study	notes
	652	form_5_complete	Section Header: *Form Status*<br>Complete?	dropdown<br>0 Incomplete<br>1 Unverified<br>2 Complete

Instrument: **Excluded From Study** (excluded_from_study) Collapse

	653	event_excluded	Event Excluded(Ky Only)	radio<br>1 Day A<br>2 Day B<br>3 Day C<br>4 Follow-up 1<br>5 Follow-up 2<br>6 Follow-up 3
	654	excluded_from_study	Excluded from study:	radio<br>1 Arrest and detention for more than 6 months<br>2 Intentional misrepresentation of the results of the study (consisted in the program of OAT at the time of screening)<br>3 Death<br>4 Withdrawal of consent to participate in the study<br>6 Not released from prison(K and M only)<br>7 Died from overdose (K M only)<br>8 Returned to prison for another crime(K M only)<br>5 Other
	655	excluded_other	Other	text
	656	excluded_from_study_complete	Section Header: *Form Status*<br>Complete?	dropdown<br>0 Incomplete<br>1 Unverified<br>2 Complete

Instrument: **Index And Partner Linkage** (index_and_partner_linkage) Collapse

	657	refer_index_id	Идентификационный МАТЛИНК код Индекса, который направил партнера:	text

	658	partner_number<br><br>Show the field ONLY if:<br>[index_partner] = '2'	Это партнер 1 или 2?	radio<br>1 | 1<br>2 | 2
	659	partner1_id<br><br>Show the field ONLY if:<br>[index_partner] = '1'	Идентификационный МАТЛИНК код Партнера 1:	text
	660	partner2_id<br><br>Show the field ONLY if:<br>[index_partner] = '1'	Идентификационный МАТЛИНК код Партнера 2:	text
	661	index_and_partner_linkage_complete	Section Header: *Form Status*<br>Complete?	dropdown<br>0 | Incomplete<br>1 | Unverified<br>2 | Complete
