# Supplementary material for: Interest without uptake: A mixed-methods analysis of methadone utilization in Kyrgyz prisons
Source: PLoS One. 2022 Oct 25;17(10):e0276723. doi: 10.1371/journal.pone.0276723 (PMC9595522; doi:10.1371/journal.pone.0276723)
Supplement: S2 File — English translation and original Russian-language interview guides used to interview study participants pre- and post-release. (DOCX) [file pone.0276723.s002.docx]

*Purpose: Establishment of confidential contact at the initial stage*

{Meet the participant, greet him, find out how he is doing, make sure that the participant understands the interview process and unobtrusively let him know that the information he will give will be very important to the interviewer and his stories will be taken seriously and with respect.}

"We would like to know more about what people who have been imprisoned in Kyrgyzstan think about drug dependence, methadone substitution maintenance therapy, as well as the risks associated with HIV, and ask you to share your experience on these issues. We would also like to hear your views on how and why some people start injecting drugs or methadone while in prison, and to hear from you about how prison staff and other inmates treat such prisoners. It's important to hear what you know about the support provided to people suffering from substance use disorders in prison and in civil society. Your input will be very helpful in developing a programme for PWID to support the provision of harm reduction services, improve their quality of life, reduce stigma and social exclusion, and reduce the risk of HIV infection.

I will ask questions and our conversation, with your permission, will be recorded on a voice recorder. We promise that your name will not be used and all responses will remain anonymous. I really want to listen to you and learn more about you and your life experiences. I am not here to assess what is right or wrong, for me the main thing is to hear your opinion. We will ask questions, some of which may be sensitive (uncomfortable) to you. In situations where this may seem uncomfortable, there are a number of ways in which you can respond. By answering questions like these, you can talk abstractly about events or activities that may be related to others, and may not be related to you, and also not name specific people who can fully identify another prisoner and/or prison employee.

If you don't want to answer a question, please tell me about it, but I'd like to ask you to be as open as possible and say whatever you think. Everything you share today will be kept secret and will only be used in anonymous form for research purposes; this information will not be shared with prison staff or other inmates. If you provide any identifying personal information, it will be removed from the interview record.

Are you comfortable with these rules? Do you have any questions before we start? {The interviewer explains any questions that may arise}. I'm going to start recording now. {Recording is started. The date of recording, the participant code, and the interviewer code are announced. This process is repeated for both pre- and post-release interviews}.

Pre-Release Interview

I. **Life in the prison**

**1. What is your role and main responsibilities in the prison?**

*Clarifying questions:*

-Are there any difficulties you face in fulfilling your duties? If so, which ones?

**II. Treatment of drug addiction**

**1. How does the *obshchak* feel about the methadone program? Why?**

*Clarifying questions:*

- Why do they live separately?

What were the main difficulties when the methadone program began in this prison? How did the attitude towards the methadone program change?

-What needs to change for the majority of drug users to go to methadone? What if a thief [gang member, leader of informal prison government] or a defendant [one of thief’s enforcers] told everyone to start methadone? And the head of the prison?

- Do methadone patients work in the dormitory? What do you mean when they say that methadone patients are "not allowed to take serious cases"? What does it mean that they are "not paid attention" from the community? Can methadone patients receive cigarettes, tea, food, etc. from the dormitory? And the [lower hierarchy levels] can?

**2. What percentage of methadone customers are combining substances?**

*Clarifying questions:*

What can be done to stop them from taking/combining methadone with other drugs?

**III. Drug use inside the prison**

**1. What percentage of all prisoners are injecting drug users in this prison?**

*Clarifying questions:*

- Is there ever "overdose" in the prison? What do they do in such cases?

- What were the reasons for the deaths that occurred this year? (About 5).

**2. Why is heroin dispersed? Since what year has it been done? How was it done when the in-prison market was still operating? Just as it is now or was it different in some way?**

*Clarifying questions:*

- Is it possible to get infected during the "dispersal"? How?

Are there drug users who cannot get heroin from the *obshchak*? How are they different?

- Why is the dispersal in the pre-trial detention center done more often than in the prison?

**3. What does it mean to live by the *ponyatiya* (prison code)? Which groups in the prison do this and which do not? Do methadone patients live according to the *ponyatiya*?**

*Clarifying questions:*

- What would have happened without an unwritten law? What would be the difference for life in prison?

- What percentage of men use heroin? And of the [lower hierarchy levels]?

- How are conflicts between prisoners resolved? And between the administration and the community?

- Does participation in the methadone program affect belonging to a particular group?

**4. What rules exist in the prison?**

*Clarifying questions:*

- Why is there a ban on diphenhydramine? And on the sale of drugs?

- Why is there no ban on the methadone program?

**IV.**  **Other issues**

**1. What is the attitude to such an interview in prisons?**

*Clarifying questions:*

- Do inmates have the right to discuss drug use with us? And what are the consequences? What could be the consequences?

What is the difference between other prisons and this one in the sense of methadone, medical services, drugs, quality of life, etc.?

Thank you very much for your time.

If you have any questions for me, please ask.

Post-Release Interview

**Life in Freedom**

**1.**  **Tell us how your life is going after your release.**

*Clarifying questions:*

- What do you do? How does a typical day go?
- What are some of the challenges you face?
- What would you recommend to a former convict who has just been released?

**II.**   **Drug Dependence** **Treatment**

**1. What should heroin users** do **to cope in prison? And at large?**

*Clarifying questions:*

-Do they need to undergo treatment? If so, which one?

-What is methadone?

-In what cases is the methadone program suitable for a drug user?

- How does the *obshchak* feel about the methadone program? Why?

-What would have to change for those who go to [the heroin distribution network] to go to methadone? On the contrary?

- Imagine the administration of the prison tells all drug users to go to methadone, and the informal prison administration tells everyone to use heroin, where would most of the prisoners go?

What is the difference between the housing of prisoners on methadone and the housing of others? What kind of work can methadone program participants do? Which one is not?

**2.**  **For what reasons can a person on methadone continue to use drugs?**

*Clarifying questions:*

*-* What percentage of methadone customers are using heroin or other substances?

What is the difference between methadone patients who are using other substances with those who are not?

What needs to change for them to stop taking/combining methadone with other drugs?

**III.**  **Drug use inside the prison**

**1. What are the options for a prisoner so that there is no withdrawal (so as not to "get sick")? And what are the options to get high?**

*Clarifying questions:*

- Is there ever "overdose" in the prison? What do they do in such cases? Where is overdose more common—in prison or out in the community?
- Why do prisoners have to carry only as much as they consume?
- What about those who start injecting for the first time in their lives in prison? How does the *obshchak* look at this? Can they get heroin from the heroin distribution?
- Would you like to have fewer, more, or as many consumers in the prison?

**2. Since what year is the heroin dispersal done in places of deprivation of liberty? How many people receive it here? How many doses is one gram divided?** **Is it possible to buy a drug from a commoner?**

*Clarifying questions:*

- Why is heroin given only every tenth day and why is its concentration lower than in the community?
- Do you think that heroin distribution is done quite often? Or should it be less often or more often? Why? Should the concentration of heroin be higher, lower, or what is it?
- Is it possible to get infected during the heroin dispensing? How? What about when heroin is not obtained from the heroin distribution network? Which is safer for health - heroin from the distribution network or heroin from another source (for example, from the administration)?
- What is the difference between heroin distribution in pre-trial detention centres and distribution in penal colonies? Why?

**3.**  **Is there any division between prisoners on any grounds?**

*Clarifying questions:*

- How would you describe [each level of the hierarchy]? Do all of them have the same access to heroin distribution?
- Does participation in the needle exchange/methadone or taking diphenhydramine/heroin affect group membership?
- Who has more control over prisoners? The [informal prison government] or the administration?

**4. What rules exist in the prisons?**

*Clarifying questions:*

- Why is there a ban on diphenhydramine? And for the sale of drugs?
- What drugs does the administration sell? Why are they doing this? What are the consequences for a prisoner who buys from the administration?
- Why is there no ban on the methadone program? Was it there before?

**IV. Ideal Prison**

**1. Imagine that you could arrange everything in the prison as you would like. Can you describe this prison?**

*Clarifying questions:*

- Who would run the prison there? Under what law would prisoners live?
- How is drug addiction treated there? Is there a needle exchange program? Methadone? Hierarchy? Why?

Thank you very much for your time.

If you have any questions for me, please ask.
