# Supplementary material for: Interest without uptake: A mixed-methods analysis of methadone utilization in Kyrgyz prisons
Source: PLoS One. 2022 Oct 25;17(10):e0276723. doi: 10.1371/journal.pone.0276723 (PMC9595522; doi:10.1371/journal.pone.0276723)
Supplement: Attachment — Submitted filename: Response to PLOS.docx [file pone.0276723.s006.docx]

135 College Street, Suite 323

New Haven, CT 06510, USA

[amanda.liberman@yale.edu](mailto:frederick.altice@yale.edu)

Michael Esguerra Alba

*PLOS One*

August 8, 2021

Dear Dr. Esguerra Alba,

Thank you for your consideration of our manuscript. Attached is a revised submission including a modified CONSORT flowchart as Figure 1 and a TREND checklist as a supplementary file. Please note that this publication is part of the MATLINK Study, Clinical Trial No. NCT04947475, currently underway in several countries in Eastern Europe and Central Asia.

We have no conflicts of interest to disclose.

Please address all correspondence concerning this manuscript to me at [amanda.liberman@yale.edu](mailto:amanda.liberman@yale.edu).

Thank you again for your consideration of this manuscript.

Sincerely,

Amanda R. Liberman

Medical Student

Yale University School of Medicine

[amanda.liberman@yale.edu](mailto:amanda.liberman@yale.edu)

Co-authors

Daniel J. Bromberg, M.Sc.
Doctoral Candidate
Yale University School of Public Health
[dan.bromberg@yale.edu](mailto:dan.bromberg@yale.edu)

Taylor Litz, MPH
Epidemiologist
Douglas County Health Department
[litzataylor@gmail.com](mailto:litzataylor@gmail.com)

Ainura Kurmanalieva, MSc
Head of the Research Unit
AIDS Foundation East-West (AFEW) in the Kyrgyz Republic
[ainura.kurmanalieva@afew.kg](mailto:ainura.kurmanalieva@afew.kg)

Samy Galvez, MPH
Associate Director of International Research, AIDS Program
Yale University School of Medicine
[samy.galvez@yale.edu](mailto:samy.galvez@yale.edu)

Julia Rozanova, PhD
Associate Research Scientist
Yale University School of Medicine
[julia.rozanova@yale.edu](mailto:julia.rozanova@yale.edu)

Lyuba Azbel, MSc, PhD
Postdoctoral Fellow
Yale University School of Medicine
[lyuba.azbel@yale.edu](mailto:lyuba.azbel@yale.edu)

Jaimie P. Meyer, MD, MS, FACP
Associate Professor
Yale University School of Medicine
[jaimie.meyer@yale.edu](mailto:jaimie.meyer@yale.edu)

Frederick L. Altice, MD

Professor of Medicine and of Epidemiology

Yale University School of Medicine
[frederick.altice@yale.edu](mailto:frederick.altice@yale.edu)
