# Supplementary material for: Interest without uptake: A mixed-methods analysis of methadone utilization in Kyrgyz prisons
Source: PLoS One. 2022 Oct 25;17(10):e0276723. doi: 10.1371/journal.pone.0276723 (PMC9595522; doi:10.1371/journal.pone.0276723)
Supplement: Attachment — Submitted filename: Reviewer Response Letter PLOS.docx [file pone.0276723.s007.docx]

June 28, 2022

Sebastian Shepherd, PhD
Associate Editor, PLOS One

Dear Dr. Shepherd,

Thank you for your careful consideration of our manuscript entitled, “Interest without Uptake: A Mixed-Methods Analysis of Methadone Utilization in Kyrgyz Prisons.” Please find below a summary of our edits, as well as a response to the comments.

Please let us know if we can provide additional clarification or revisions. Thank you again for your consideration of our manuscript.

Sincerely,

Amanda R. Liberman, BA
Medical Student
Yale University
[amanda.liberman@yale.edu](mailto:amanda.liberman@yale.edu)

Co-authors

Daniel J. Bromberg, M.Sc.
Doctoral Candidate
Yale University School of Public Health
[dan.bromberg@yale.edu](mailto:dan.bromberg@yale.edu)

Taylor Litz, MPH
Epidemiologist
Douglas County Health Department
[litzataylor@gmail.com](mailto:litzataylor@gmail.com)

Ainura Kurmanalieva, MSc
Head of the Research Unit
AIDS Foundation East-West (AFEW) in the Kyrgyz Republic
[ainura.kurmanalieva@afew.kg](mailto:ainura.kurmanalieva@afew.kg)

Samy Galvez, MPH
Associate Director of International Research, AIDS Program
Yale University School of Medicine
[samy.galvez@yale.edu](mailto:samy.galvez@yale.edu)

Julia Rozanova, PhD
Associate Research Scientist
Yale University School of Medicine
[julia.rozanova@yale.edu](mailto:julia.rozanova@yale.edu)

Lyuba Azbel, MSc, PhD
Postdoctoral Fellow
Yale University School of Medicine
[lyuba.azbel@yale.edu](mailto:lyuba.azbel@yale.edu)

Jaimie P. Meyer, MD, MS, FACP
Associate Professor
Yale University School of Medicine
[jaimie.meyer@yale.edu](mailto:jaimie.meyer@yale.edu)

Frederick L. Altice, MD

Professor of Medicine and of Epidemiology

Yale University School of Medicine
[frederick.altice@yale.edu](mailto:frederick.altice@yale.edu)

**Point-by-point Response to Reviewer Feedback**

1. *Please ensure that your manuscript meets PLOS ONE's style requirements, including those for file naming. The PLOS ONE style templates can be found at*
   [*https://journals.plos.org/plosone/s/file?id=wjVg/PLOSOne_formatting_sample_main_body.pdf*](https://nam12.safelinks.protection.outlook.com/?url=https%3A%2F%2Fjournals.plos.org%2Fplosone%2Fs%2Ffile%3Fid%3DwjVg%2FPLOSOne_formatting_sample_main_body.pdf&data=05%7C01%7Camanda.liberman%40yale.edu%7Ca7db87e41d6b476f09da08da439e8eee%7Cdd8cbebb21394df8b4114e3e87abeb5c%7C0%7C0%7C637896645681429988%7CUnknown%7CTWFpbGZsb3d8eyJWIjoiMC4wLjAwMDAiLCJQIjoiV2luMzIiLCJBTiI6Ik1haWwiLCJXVCI6Mn0%3D%7C3000%7C%7C%7C&sdata=y1NpSV1RLfUAfVw1bWCdS7IYCBntv8CnZ%2BcDII68mq8%3D&reserved=0) *and* 
   [*https://journals.plos.org/plosone/s/file?id=ba62/PLOSOne_formatting_sample_title_authors_affiliations.pdf*](https://nam12.safelinks.protection.outlook.com/?url=https%3A%2F%2Fjournals.plos.org%2Fplosone%2Fs%2Ffile%3Fid%3Dba62%2FPLOSOne_formatting_sample_title_authors_affiliations.pdf&data=05%7C01%7Camanda.liberman%40yale.edu%7Ca7db87e41d6b476f09da08da439e8eee%7Cdd8cbebb21394df8b4114e3e87abeb5c%7C0%7C0%7C637896645681429988%7CUnknown%7CTWFpbGZsb3d8eyJWIjoiMC4wLjAwMDAiLCJQIjoiV2luMzIiLCJBTiI6Ik1haWwiLCJXVCI6Mn0%3D%7C3000%7C%7C%7C&sdata=UWAIcR26wAVxWG5Qp2%2Fd4Ik9g1mvWEyx791xuwinr3I%3D&reserved=0)

   **Response:** Thank you for these clarifying documents. We have updated our manuscript to reflect these formatting changes.
2. *Please provide additional details regarding participant consent. In the ethics statement in the Methods and online submission information, please ensure that you have specified what type you obtained (for instance, written or verbal, and if verbal, how it was documented and witnessed). If your study included minors, state whether you obtained consent from parents or guardians. If the need for consent was waived by the ethics committee, please include this information.*

**Response:** Previously, our manuscript had included the following: “If OUD was confirmed, potential participants completed informed consent procedures in which research assistants made clear that this study was not affiliated with the prison administration, that surveys and interviews would remain anonymous, and that participants could withdraw from the study at any point.” We have updated our text to add: “Each participant was given a copy of the informed consent document to read and provided with ample time to read the form and to ask questions. To minimize potential coercion, every participant was read a statement that included the following: “Participation in the study is voluntary. Refusal or consent to participate will not affect the change of conditions of registration in prison and the information will be kept confidential. Personal information about your medical condition or any other information will be available only to you and for research purposes.” Participants were able to opt out of the study at any time and were assured that participation, refusal to participate, or discontinuation of participation were not linked with any rewards or punishments.”

3. *In the Methods section of the manuscript, please provide additional information regarding how participants were recruited for the qualitative study, please specify whether an interview guide was used to interview the participants in your study. If yes, please describe and/or include a copy as a Supporting Information file, and finally, please consider including more information on the number of interviewers, their training and characteristics.*

   **Response:** Thank you for this feedback. We have modified our information about recruitment: “Briefly, the Department of Penitentiary Institutions in the Kyrgyz Republic provided the study coordinator with a list of all incarcerated persons within 180 days of scheduled release or possible early release. (Those with less than eight days until their release date were excluded.) Research personnel screened all incarcerated persons who met these criteria using a single-item screener for opioid use disorder (OUD) followed by a pre-incarceration assessment of opioid use in the 30 days before incarceration. Those who screened positive then underwent additional screening for opioid dependence using the validated Rapid Opioid Dependence Scale.”

   For interview guides, English translations of pre- and post-interview guides have been included as File S2.

   Additional information about interviewers was provided as follows: “LA and JR conducted the interviews in Russian, although participants were given the option to have their interviews in Kyrgyz with a trained research associate if they preferred. All participants chose to have their interviews conducted in Russian.”
4. *Please provide additional information regarding the considerations made for the prisoners included in this study. For instance, please discuss whether participants were able to opt out of the study and whether individuals who did not participate receive the same treatment offered to participants.*

   **Response:** Please see response to Point 2 above.
5. *Registration done retrospectively (after enrollment of participants) (TC2/PRTC Note)
   Thank you for submitting your clinical trial to PLOS ONE and for providing the name of the registry and the registration number. The information in the registry entry suggests that your trial was registered after patient recruitment began. PLOS ONE strongly encourages authors to register all trials before recruiting the first participant in a study.
   As per the journal’s editorial policy, please include in the Methods section of your paper:*
   1) *your reasons for your delay in registering this study (after enrolment of participants started);*
   2) *confirmation that all related trials are registered by stating: “The authors confirm that all ongoing and related trials for this drug/intervention are registered.*

**Response:** The authors confirm that all ongoing and related trials for this drug/intervention are registered. This trial was registered in clinicaltrials.gov on July 1, 2021 which was after enrollment began. While the original intention was to register the study prior to enrollment, this delay was due to a change in staff at the beginning of the study. The timeline of enrollment and data collection for this paper’s self-reported preliminary data (2017-2021) is outlined in the results section (first sentence).

6. *During the internal evaluation of the manuscript, we have noted some discrepancies between the study protocol and the manuscript text. In particular please could you provide some clarification on the following:*
   1) *The protocol indicated that the study will be conducted within 7 prisons, however the manuscript text implies that 9 prisons were included. Please could you clarify whether the IRB approved this deviation.*
   2) *A sample size of 120 participants was calculated in the study protocol, however 125 participants were included in the study as reported in the text. As such please could you clarify whether the IRB approved for the inclusion of additional participants in the study.*

*Furthermore, please could you provide a description of the intervention of the clinical trial and please also report the expected primary and secondary outcomes of the study within the Methods section.*

**Response:** We have updated our information about the intervention to include the following: “Next, [study subjects] participated in a brief intervention (BI) guided by motivational interviewing principles in which a trained research assistant explained benefits and dispelled myths relating to methadone treatment both during and after incarceration. This BI had two aims. First, the BI was designed to inform potential participants on the risks of substance misuse, abuse, and dependency by illustrating the potential hazards and adverse health consequences. Second, the BI aimed to motivate potential participants to reduce risky behavior (e.g., continued drug use) and seek treatment for their substance dependence disorder. After the BI, participants’ interest in methadone was re-assessed as above, and if they were interested, they were referred to a treating physician in the prison to initiate methadone. All participants, irrespective of methadone enrollment, underwent a second BI one week before release to encourage study participants on methadone to link to care or those not on methadone to begin methadone after release.

During each BI, research team members provided evidence-based information on methadone. This information was available to study participants in the community upon release. Study participants were informed of the risks and benefits of methadone, and during the second BI, they learned how to access methadone in their communities. Each BI lasted approximately 20 minutes, and afterwards, participants were provided time for questions. The BIs were audio recorded; audio files are available upon request (files only available in the original languages of the BI—Russian & Kyrgyz).”

We have also added study outcomes: “Primary study outcomes included initiation of methadone, retention in methadone treatment, relapse to heroin, and recidivism.”

As for sample size, the IRB states that we will aim to recruit 120 people into the intervention, as a minimum target. Because the first step of the SBIRT procedure is S-Screening of all eligible participants, there is no way to know how many will screen positive for OUD, with 120 being a general benchmark. This sample size is allowed by the IRB.

*Finally, please provide additional information regarding the considerations  made for the prisoners included in this study. For instance, please discuss whether participants were able to opt out of the study and whether individuals who did not participate receive the same treatment offered to participants.*

**Response:** Please see response to Point 2 above.

7. *In your Data Availability statement, you have not specified where the minimal data set underlying the results described in your manuscript can be found. PLOS defines a study's minimal data set as the underlying data used to reach the conclusions drawn in the manuscript and any additional data required to replicate the reported study findings in their entirety. All PLOS journals require that the minimal data set be made fully available. For more information about our data policy, please see* [*http://journals.plos.org/plosone/s/data-availability*](https://nam12.safelinks.protection.outlook.com/?url=http%3A%2F%2Fjournals.plos.org%2Fplosone%2Fs%2Fdata-availability&data=05%7C01%7Camanda.liberman%40yale.edu%7Ca7db87e41d6b476f09da08da439e8eee%7Cdd8cbebb21394df8b4114e3e87abeb5c%7C0%7C0%7C637896645681429988%7CUnknown%7CTWFpbGZsb3d8eyJWIjoiMC4wLjAwMDAiLCJQIjoiV2luMzIiLCJBTiI6Ik1haWwiLCJXVCI6Mn0%3D%7C3000%7C%7C%7C&sdata=tMjYbogWYcwayAVcSPXCwfAFjh8E%2FpbihfdQKv6Hdb4%3D&reserved=0)*.*
   *Upon re-submitting your revised manuscript, please upload your study’s minimal underlying data set as either Supporting Information files or to a stable, public repository and include the relevant URLs, DOIs, or accession numbers within your revised cover letter. For a list of acceptable repositories, please see* [*http://journals.plos.org/plosone/s/data-availability#loc-recommended-repositories*](https://nam12.safelinks.protection.outlook.com/?url=http%3A%2F%2Fjournals.plos.org%2Fplosone%2Fs%2Fdata-availability%23loc-recommended-repositories&data=05%7C01%7Camanda.liberman%40yale.edu%7Ca7db87e41d6b476f09da08da439e8eee%7Cdd8cbebb21394df8b4114e3e87abeb5c%7C0%7C0%7C637896645681429988%7CUnknown%7CTWFpbGZsb3d8eyJWIjoiMC4wLjAwMDAiLCJQIjoiV2luMzIiLCJBTiI6Ik1haWwiLCJXVCI6Mn0%3D%7C3000%7C%7C%7C&sdata=x1Ov%2BpGD38BOsG5BZAskHdlJZw%2FIWBfLA6jJ0gDrNw8%3D&reserved=0)*. Any potentially identifying patient information must be fully anonymized.*
   *Important: If there are ethical or legal restrictions to sharing your data publicly, please explain these restrictions in detail. Please see our guidelines for more information on what we consider unacceptable restrictions to publicly sharing data:* [*http://journals.plos.org/plosone/s/data-availability#loc-unacceptable-data-access-restrictions*](https://nam12.safelinks.protection.outlook.com/?url=http%3A%2F%2Fjournals.plos.org%2Fplosone%2Fs%2Fdata-availability%23loc-unacceptable-data-access-restrictions&data=05%7C01%7Camanda.liberman%40yale.edu%7Ca7db87e41d6b476f09da08da439e8eee%7Cdd8cbebb21394df8b4114e3e87abeb5c%7C0%7C0%7C637896645681429988%7CUnknown%7CTWFpbGZsb3d8eyJWIjoiMC4wLjAwMDAiLCJQIjoiV2luMzIiLCJBTiI6Ik1haWwiLCJXVCI6Mn0%3D%7C3000%7C%7C%7C&sdata=5U1NmGZ3%2B78yWIdLOyGYiCvRgfmG49t4D2lf13gwIOg%3D&reserved=0)*. Note that it is not acceptable for the authors to be the sole named individuals responsible for ensuring data access.*
   *We will update your Data Availability statement to reflect the information you provide in your cover letter.*

   **Response:** We have uploaded the underlying quantitative data as a Supporting Information file.
8. *Please note that in order to use the direct billing option the corresponding author must be affiliated with the chosen institute. Please either amend your manuscript to change the affiliation or corresponding author, or email us at* [*plosone@plos.org*](mailto:plosone@plos.org) *with a request to remove this option.*

   **Response:** The corresponding author is affiliated with Yale University, the chosen institute. These authors were unclear of the need for clarification on this point.
9. *Your abstract cannot contain citations. Please only include citations in the body text of the manuscript, and ensure that they remain in ascending numerical order on first mention.*

   **Response:** Thank you for this feedback. The abstract no longer contains citations.
10. *We note you have included a table to which you do not refer in the text of your manuscript. Please ensure that you refer to Table 1a and 1b in your text; if accepted, production will need this reference to link the reader to the Table.*

    **Response:** Thank you for this feedback. The original manuscript referenced Table 1, without specifying Tables 1a and 1b in particular. We have updated the reference to Table 1 to specify the individual sub-tables.
11. *Please include captions for your Supporting Information files at the end of your manuscript, and update any in-text citations to match accordingly. Please see our Supporting Information guidelines for more information:* [*http://journals.plos.org/plosone/s/supporting-information.*](http://journals.plos.org/plosone/s/supporting-information. )

    **Response:** The authors appreciate this helpful list of guidelines. Our initial manuscript did not contain Supporting Information files, but we have added these in response to reviewer/editor comments. We have added the captions at the end of the manuscript as requested.

Reviewer #1: My comments are as follows:

1. *The manuscript has been submitted as a Clinical Trial, but a thorough reading appears that it is an observational study. However, it turns out that the study is registered in clinicaltrials.gov, with a valid NCT number. More details are needed on why the authors think that this is indeed a clinical trial. It appears there is no randomization, if I am not mistaken. If the study is claimed to be a Clinical Trial, CONSORT guidelines should be followed in reporting the results, or arguments needed on why it maybe ignored.*

   **Response:** The authors were requested to disregard this point, per editorial staff.
2. *The statistical analysis plan appears mixed up with other information in the Methods section. A separate subsection is desired, which should clearly mention the tests to be used, and what would be the alternatives when standard Gaussian assumptions fail (and where paired t-tests are invalid). Relevant nonparametric method should be stated here.*

   **Response:** The authors appreciate this correction, and have created a separate sub-section for statistical analysis. Relevant nonparametric methods have been used, as suggested.
3. *It was really strange to see that the authors didn't provide a sample size/power statement, based on a target effect size they wanted to achieve. This would allow efficient planning to similar future studies. The sample size/power should be computed based on the primary outcome, say at 5% level, and likely one that achieves 80-90% power.*

   **Response:** This study tests the SBIRT method of recruiting people into OAT. During the S-Screening step, we screen all potentially eligible participants (people to be released from Kyrgyz prisons that have MMT) for opioid use disorder. Only those that screen positive are then included in the sample. In our study protocol, we present the target sample as 120 people as approximately 1000 people are released from the Kyrgyz prisons that have MMT every year, as we expected approximately 120 of them to be eligible for inclusion. Indeed, this number was fairly close as we recruited 125 people into the sample.

   For the analysis of whether the B-brief intervention component was effective, we cannot recruit more people than there are eligible participants in the country; therefore, a formal sample size calculation was not included.
4. *The study is longitudinal; it is not clear why a formal longitudinal analysis was not constructed, using mixed-effects models.*

   **Response:** In the analysis we compare OAT interest, knowledge, and attitude scores before and after the brief intervention component of SBIRT, as the goal of this analysis is to determine whether the brief intervention is successful. If we had indication that the intervention might have been successful, we would have been interested in doing a more thorough analysis to make sure that we conservative in our claims. However, the simple analysis presented in the paper did not show a significant difference in scores before/after the intervention. Therefore, we can be confident in the claims we make to the reader.
5. *The analysis is a bit compromised, given that the collected data is "clustered" in nature since there are 9 prisons in total, and subjects recruited in a specific prison appear to be clustered. If one doesn't want to utilize clustered paired tests, or the alternatives, sufficient justification is necessary.*

   **Response:** Clustered analyses e.g. clustered t-tests, mixed/multilevel models are more conservative as they account for potential additional effects from clustering. As the simple paired t-test was not found to be significant in our analysis, we can be confident that it will be even less significant if we account for additional factors like clustering.

*Reviewer #2: This paper addresses the important issue regarding the availability of agonist maintenance therapy to opioid addicted prisoners. Its focus on the Kyrgyz Republic is unique in view of the absence of similar information in other former Soviet Republics. It could be improved by attention to the following points:*

a) *describe methadone as an evidence-based treatment that reduced opioid use, risk for HIV and opioid overdose, improves overall functioning, and increases the chances for engagement in addiction and other relevant medical treatments. Describing it as the “gold standard” introduces a value judgment that is not necessary.*

   **Response:** The authors appreciate this feedback, and have revised their original statement about methadone as follows: “Medication-assisted therapy with opioid agonists is an evidence-based HIV-prevention strategy that decreases the risk of opioid overdose, improves overall functioning, and increases engagement with other medical treatments [1–3].”
b) *the paper focuses on prisoners who do not want methadone treatment, but it looks like about 20% of the prisoners were on it at the time of incarceration and continued it in prison. This finding needs more emphasis.*

   **Response:** The authors appreciate this astute observation. We have added the following: “Twenty-three percent of study participants were already participating in the methadone program at the time of the study. It is possible that individuals who were planning on initiating methadone therapy may have already done so.”
c) *Can more detail be provided about the “prison hierarchy”? How do prisoners get slotted into the three categories?*

   **Response:** Thank you for this feedback. The authors have aimed to provide clarification by adding the following: “Briefly, when a person arrives into prison, he encounters a tribunal of his peers, which assesses whether he is guilty of the crime for which he has been incarcerated, the severity of his crime, and any mitigating factors (for example, positive character references from community members).”
d) *Were the 11.2% who screened positive addicted to opioids?*

   **Response:** Yes; these individuals screened positive for opioid use disorder (OUD). The manuscript has been updated to clarify this point.
e) *Findings in Tables 1b and 1c can likely be summarized in the text.*

   **Response:** While the authors understand the reviewer’s suggestion, we believe that these tables allow for a more in-depth portrayal of participant characteristics and provide visual comparison between those who completed and those who chose not to complete the study.
f) *Any thoughts about how to increase acceptability of methadone among prisoners who are not receiving it?* **Response:** Thank you for this point of clarification. As we specify in the original manuscript, “Given these findings, future methadone program implementation would likely be more successful within-prison if paired with continued access to NSP, if different hierarchy statuses received methadone from different locations, and if methadone were explicitly dissociated from connections to the formal prison administration.”
