# Supplementary material for: Interest without uptake: A mixed-methods analysis of methadone utilization in Kyrgyz prisons
Source: PLoS One. 2022 Oct 25;17(10):e0276723. doi: 10.1371/journal.pone.0276723 (PMC9595522; doi:10.1371/journal.pone.0276723)
Supplement: Attachment — Submitted filename: Reviewer Response Letter 9.8.docx [file pone.0276723.s008.docx]

September 8, 2022

Hugh Cowley, MSci
Staff Editor, PLOS One

Dear Mr. Cowley,

Thank you for your careful consideration of our manuscript entitled, “Interest without Uptake: A Mixed-Methods Analysis of Methadone Utilization in Kyrgyz Prisons.” Please find below a summary of our edits, as well as a response to the comments.

Please let us know if we can provide additional clarification or revisions. Thank you again for your consideration of our manuscript.

Sincerely,

Amanda R. Liberman, BA
Medical Student
Yale University
[amanda.liberman@yale.edu](mailto:amanda.liberman@yale.edu)

Co-authors

Daniel J. Bromberg, M.Sc.
Doctoral Candidate
Yale University School of Public Health
[dan.bromberg@yale.edu](mailto:dan.bromberg@yale.edu)

Taylor Litz, MPH
Epidemiologist
Douglas County Health Department
[litzataylor@gmail.com](mailto:litzataylor@gmail.com)

Ainura Kurmanalieva, MSc
Head of the Research Unit
AIDS Foundation East-West (AFEW) in the Kyrgyz Republic
[ainura.kurmanalieva@afew.kg](mailto:ainura.kurmanalieva@afew.kg)

Samy Galvez, MPH
Associate Director of International Research, AIDS Program
Yale University School of Medicine
[samy.galvez@yale.edu](mailto:samy.galvez@yale.edu)

Julia Rozanova, PhD
Associate Research Scientist
Yale University School of Medicine
[julia.rozanova@yale.edu](mailto:julia.rozanova@yale.edu)

Lyuba Azbel, MSc, PhD
Postdoctoral Fellow
Yale University School of Medicine
[lyuba.azbel@yale.edu](mailto:lyuba.azbel@yale.edu)

Jaimie P. Meyer, MD, MS, FACP
Associate Professor
Yale University School of Medicine
[jaimie.meyer@yale.edu](mailto:jaimie.meyer@yale.edu)

Frederick L. Altice, MD

Professor of Medicine and of Epidemiology

Yale University School of Medicine
[frederick.altice@yale.edu](mailto:frederick.altice@yale.edu)

**Point-by-point Response to Reviewer Feedback**

1. *Outcome results are clear but the text is hard to read and could be shortened by 40-50%.*

The authors thank the reviewer for this suggestion. This version of the text has been revised and shortened from 8742 words to 7098 words.

2. *The authors mix stigma with negative attitudes about methadone maintenance (MM). Stigma applies to addiction in general; MM has its own stigma within the overall stigma of addiction.*

The authors have applied the reviewer’s suggestion and have edited the section about stigma to clarify.

3. *The negative attitudes about MM may represent what can happen when MM is started in a country where there have been laws against it for many years. I presume this is true in the Kyrgyz Republic since it likely had laws prohibiting use of opioids for treating opioid addiction when it was part of the Soviet Union. The authors might consider adding information about similar attitudes in the U.S. Examples are that MM “eats up your insides”; it “gets into your bones”; it is “just another drug”, not a rx; that people on mm are “weak”.*

The authors thank the author for this suggestion and have added the following sentence (lines 366-369): “Many of these beliefs regarding methadone and methadone program participants exist in North America as well, including that methadone program participation indications a lack of will power, untrustworthiness, or ongoing addiction (i.e., to methadone instead of heroin) [42,43].”

4. *The BNDD tried to arrest Dr. Dole, and it might be interesting to document it if one or more references can be found. It’s an excellent example of the very negative police response in the early day of methadone. Wyoming still may not allow MM and if so, could be an example of this lingering negativity about MM.*

While the authors were not able to find a reference regarding the attempted arrest of Dr. Dole, they have added a source regarding police harassment of MM patients in the United States (lines 378-380): “Similar findings have also been reported… in the United States, where interviewees for a newspaper article described being followed from their methadone clinics by police and arrested for minor traffic infractions [47].”

5. *Consider mentioning DSM-5 and ICD-11 where a patient can be in remission if on MM, buprenorphine, or naltrexone.*

The authors thank the reviewer for this suggestion. However, they have chosen not to include these given the differing definitions of remission in the DSM-5-TR vs the ICD-11. The ICD-11 defines “sustained full remission” as “After a diagnosis of Opioid dependence, and often following a treatment episode or other intervention (including self-intervention), the person has been abstinent from opioids for 12 months or longer ([ICD-11 for Mortality and Morbidity Statistics (who.int)](https://icd.who.int/browse11/l-m/en#/http%3a%2f%2fid.who.int%2ficd%2fentity%2f36859167)). Meanwhile, the DSM-5-TR allows methadone patients to receive a diagnosis of early or sustained remission, as long as the specifier “on maintenance therapy” is added ([Psychiatry Online | DSM Library](https://dsm.psychiatryonline.org/doi/full/10.1176/appi.books.9780890425787.x16_Substance_Related_Disorders)). Given these differing definitions, we have chosen not to mention either definition of remission in this text.

6. *Consider mentioning that MM is available in prisons in most EU countries. Modify the statement that the Kyrgyz Republic is one of “few countries”.*

We thank the reviewer for this suggestion, and we have modified the statement in question (line 29).

7. *Add a comment on how SBIRT has shown weak to modest effectiveness in the U.S. and not studies on former Soviet States.*

We thank the reviewer for this suggestion, and we have modified lines 67-70 to reflect this point.

8. *can more details be added about how new arrivals are classified within the prison system. The system the authors describe is unique.*

As described in lines 276-280, “Briefly, when a person arrives into prison, he encounters a tribunal of his peers, which assesses whether he is guilty of the crime for which he has been incarcerated, the severity of his crime, and any mitigating factors (for example, positive character references from community members). He is then assigned a hierarchy status based on this assessment (Table 1b).”

9. *translations of Russian names relevant here.*

The authors have attempted to remove as many of the Russian transliterations as possible to encourage ease of reading.

10. *Is the prison infrastructure re methadone use common to all prisons? If so, could its description be shortened?*

As described in the text (line 274), this infrastructure is common to men’s prisons in the Kyrgyz Republic. Its description has been shortened as requested.

11. *what is the relationship between prison management and the way prisoners are classified? It appears as if prisoners are put into the groupings by other prisoners.*

To answer the reviewer’s question, incarcerated people are put into the groupings by their peers, as described in lines 276-280. This is an informal prison governmental system, not affiliated with the official prison administration (more details about this relationship have been described in a previous paper, Liberman et al. 2021).

12. *does the MOH put out info on MM?*

The authors thank the reviewer for this question, and have added a note about MOH guidelines for MM (lines 57-59).

13. *consider limiting the number of English translations of Russian words; repeating the same words makes the paper more difficult to read*

The authors thank the reviewer for this suggestion, and have attempted to eliminate as many English transliterations of Russian words as possible to promote ease of reading throughout the text.

14. *It’s not surprising that few started MM in view of the negative social pressure about it. Comments along those lined could be added since this negativity may explain why so few who were not on methadone started it.*

The authors thank the reviewer for this suggestion, highlighted in lines 365-367: “These ideas of sobriety and personal/community beliefs about methadone and methadone users interacted to discourage methadone uptake.”
