# Supplementary material for: Interest without uptake: A mixed-methods analysis of methadone utilization in Kyrgyz prisons
Source: PLoS One. 2022 Oct 25;17(10):e0276723. doi: 10.1371/journal.pone.0276723 (PMC9595522; doi:10.1371/journal.pone.0276723)
Supplement: Attachment — Submitted filename: Reviewer Response Letter 9.30.docx [file pone.0276723.s009.docx]

September 30, 2022

Judith I. Tsui
Academic Editor, PLOS One

Dear Dr. Tsui,

Thank you for your careful consideration of our manuscript entitled, “Interest without Uptake: A Mixed-Methods Analysis of Methadone Utilization in Kyrgyz Prisons.” Please find below a summary of our edits, as well as a response to the comments.

Please let us know if we can provide additional clarification or revisions. Thank you again for your consideration of our manuscript.

Sincerely,

Amanda R. Liberman, B.A.
Medical Student
Yale University
[amanda.liberman@yale.edu](mailto:amanda.liberman@yale.edu)

Co-authors

Daniel J. Bromberg, M.Sc.
Doctoral Candidate
Yale University School of Public Health
[dan.bromberg@yale.edu](mailto:dan.bromberg@yale.edu)

Taylor Litz, MPH
Epidemiologist
Douglas County Health Department
[litzataylor@gmail.com](mailto:litzataylor@gmail.com)

Ainura Kurmanalieva, MSc
Head of the Research Unit
AIDS Foundation East-West (AFEW) in the Kyrgyz Republic
[ainura.kurmanalieva@afew.kg](mailto:ainura.kurmanalieva@afew.kg)

Samy Galvez, MPH
Associate Director of International Research, AIDS Program
Yale University School of Medicine
[samy.galvez@yale.edu](mailto:samy.galvez@yale.edu)

Julia Rozanova, PhD
Associate Research Scientist
Yale University School of Medicine
[julia.rozanova@yale.edu](mailto:julia.rozanova@yale.edu)

Lyu Azbel, MSc, PhD
Postdoctoral Fellow
Yale University School of Medicine
[lyuba.azbel@yale.edu](mailto:lyuba.azbel@yale.edu)

Jaimie P. Meyer, MD, MS, FACP
Associate Professor
Yale University School of Medicine
[jaimie.meyer@yale.edu](mailto:jaimie.meyer@yale.edu)

Frederick L. Altice, MD

Professor of Medicine and of Epidemiology

Yale University School of Medicine
[frederick.altice@yale.edu](mailto:frederick.altice@yale.edu)

**Point-by-point Response to Reviewer Feedback**

1. *The manuscript has been submitted as a Clinical Trial! I was surprized to see that there is no mention of a formal sample size/power calculation, and the study data has been analyzed, without any perspective on that. That needs to be added, or justification provided on why that is missing!*

The authors thank the reviewer for this suggestion. This was addressed in a previous round of reviews (7.27.22), and the original comment and response are provided below. Additionally, the text has been updated to incorporate the answer provided below:

“Comment: *It was really strange to see that the authors didn't provide a sample size/power statement, based on a target effect size they wanted to achieve. This would allow efficient planning to similar future studies. The sample size/power should be computed based on the primary outcome, say at 5% level, and likely one that achieves 80-90% power.*

**Response:** This study tests the SBIRT method of recruiting people into OAT. During the S-Screening step, we screen all potentially eligible participants (people to be released from Kyrgyz prisons that have MMT) for opioid use disorder. Only those that screen positive are then included in the sample. In our study protocol, we present the target sample as 120 people as approximately 1000 people are released from the Kyrgyz prisons that have MMT every year, as we expected approximately 120 of them to be eligible for inclusion. Indeed, this number was fairly close as we recruited 125 people into the sample.
For the analysis of whether the B-brief intervention component was effective, we cannot recruit more people than there are eligible participants in the country; therefore, a formal sample size calculation was not included.”

2. *Can the authors provide details about heroin availability in the prisons? In the U.S. heroin distribution would not be tolerate. How is this situation managed in the prisons? It appears to be an informal distribution network that is tolerate by prison administrators. Do all the guards participate or only a few? More details would be interesting if possible.*

The authors thank the reviewer for this suggestion. They have added the following text: “The obshchak was highly motivated to dissuade people from using methadone, because the *obshchak* was the major distributer of in-prison heroin. This process was facilitated by the *obshchak*'s extensive connections outside of the prison. It acted both as a mutual aid fund, collecting and redistributing goods to incarcerated people, and also as a marketplace for various goods and services. This marketplace was facilitated by corruption of official prison staff, who allowed these goods, including heroin, to enter the prison [11]. Heroin served both as a commodity and as a form of currency which could be used to purchase other items within the prison.”

It is difficult to estimate a percentage of official prison staff who participate in the informal heroin distribution system, so this specific detail was not included.

3. *How is methadone treatment administered in the prisons? Do prisoners come to medical units for daily observed dosing? Is methadone brought to them by medical staff every day?*

The authors thank the reviewer for this suggestion and have added the following: “Prison medical staff administered methadone daily at a specific, designated location for all methadone program participants irrespective of hierarchy level. Therefore, joining the methadone program meant potential physical interaction with people of lower hierarchy status or using the same items, like pens or cups. These interactions could lead to demotion within the hierarchy [29].”

4. *Where is the NSP located and how to prisoners access it?*

The authors thank the reviewer for this clarification point. The specific location of the NSP depends on the prison; some are located in the medical area, whereas other are in the barracks. Participants register confidentially in the program, and there is a low threshold for entry. Nurses distribute the clean equipment. Although the NSP is not a central focus of this paper, we have clarified this in the text with the following: “Ordinarily, to access the NSP, individuals registered confidentially and were then able to receive injection equipment from nurses located either in the medical area or the barracks, depending on the specific prison. However, those that joined the methadone program were no longer permitted to participate in the NSP.”
